# Supplementary material for: Do female sex workers have lower uptake of HIV treatment services than non-sex workers? A cross-sectional study from east Zimbabwe
Source: BMJ Open. 2018 Feb 28;8(2):e018751. doi: 10.1136/bmjopen-2017-018751 (PMC5855339; doi:10.1136/bmjopen-2017-018751)
Supplement: Supplementary file 1 [file bmjopen-2017-018751supp001.pdf]

Supplementary Material

A. Theoretical framework for factors mediating uptake of HIV services among female sex workers vs non-sex workers

Several theoretical models of the mechanisms for behaviour change have been successfully applied to reduce risky sexual behaviour by individuals [1]. However, recognition of the limitations of individual-level approaches to HIV prevention (such as to what extent condom use is solely related to self-efficacy without consideration of gendered power dynamics) has led to a growth in structural models for HIV risk [2]. Multilevel theoretical models draw on the strengths of both individual-level focused models and structural models, but in delineating the links between these levels have great potential power for theory-driven approaches to combination prevention [3]. The Network-Individual-Resource Model (NIRM) for HIV Prevention posits that membership of distinct social networks can attenuate or enhance individual-level factors driving HIV risk [4]. For example, intense stigma and discrimination frequently marginalises female sex workers (FSW) from wider society such that FSW may occupy distinct social networks from non-sex workers (NSW). Therefore, various factors relating these distinct social networks may mean that FSWs' use of healthcare services may be dissimilar to that of NSW. These factors comprise individual-level preferences and behaviours as well as relations with peers, relatives and the community and the legal and socio-cultural context. Drawing on a rich literature of multi-level theoretical approaches to behaviour change and HIV transmission prevention [3–5], we describe a new framework (Figure S1) to explain how intermediate factors at different levels may be associated with HIV service uptake in

testable relations in a Zimbabwean context, a subset of which are explored in this paper (Figure 1).

Supplementary Figure 1: Generalised theoretical framework for intermediate factors mediating differential uptake of HIV services by female sex workers (FSW) compared to non-sex workers (NSW).

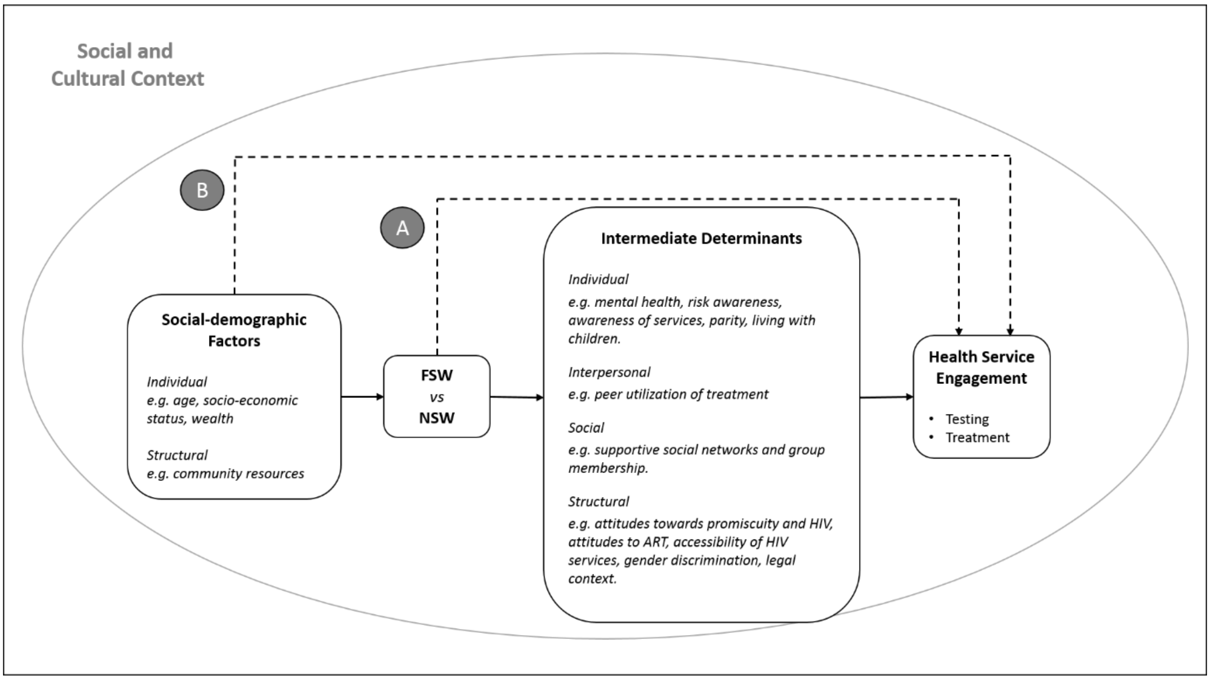

Influence of structural factors on uptake of health services

Structural factors include social, cultural, economic, legal and political contexts which shape and frame behaviours, actions and norms of communities and agents [2]. In meta-analyses evaluating barriers to retention and linkage to care, distance from testing facilities and costs of transport have been identified as the most important obstacles [6], yet to what extent this is true in FSW as well as NSW is unclear. Since FSW tend to most commonly live in more urban areas than NSW [17] where facilities are most concentrated and more closely available, we might expect distances and costs of transport to be different between FSW and NSW. However, travel also incurs an opportunity cost for FSW who experience loss of earnings during the time taken to travel [7] so this may attenuate their uptake relative to NSW. In addition, FSW often migrate both internally and across national borders [8,9], often away from families even including children. Mobility and migration affect uptake of services in complex ways that are dependent on a variety of contextual factors (e.g. relative availability of services in source and sink destinations, whether migration is internal, circular or international). Migration from high to low prevalence settings is associated with lower HIV risk [10] but migrants are more likely to be unaware of local services which can reduce access. In addition, circular migration can interrupt treatment or cause delays in treatment [11].

Fear of social rejection and discrimination from positive HIV diagnosis often deters individuals from seeking testing [7,12,13]. For FSW, this fear may be more intense because of higher rates of HIV among FSW than NSW (see individual-factors below) and because they already experience intense stigma and discrimination as a result of selling sex. Laws criminalising sex work mean that sex workers are often subject to arrest and violence perpetrated by police [2,14,15]. Such laws often intersect with gendered attitudes towards acceptable behaviour for women, often compounding long-lived taboos around female promiscuity [16,17]. Such stigma

frequently results in harassment [16] and can cause unnecessary delays to treatment from healthcare workers [18] or deter FSW from accessing care altogether [19].

Influence of social factors on uptake of health services

Evidence for social factors influencing uptake is based on trials of various interventions to encourage treatment initiation and adherence. In the wider community, mobilisation, group membership and empowerment (either informal or formal) have been successful in encouraging HIV testing and treatment through enhancing social capital (networks of inter-group relationships that are socially enhancing) and self-efficacy [20,21]. Similar approaches have been targeted towards mobilising sex worker communities (e.g. by uniting sex workers in a common cause for health improvement, creating spaces for debate of new health information and tackling powerful actors that actively disenfranchise sex workers through violence, stigma or discrimination [22] have demonstrated substantial effectiveness in reducing HIV infection and other STIs and increasing condom use [23]. An important component of community mobilisation is the development and strengthening of social capital and facilitating “transformative social spaces”. One approach to this is encouraging participation in community groups. Such groups can have powerful positive impacts on risk behaviours and healthcare seeking, by providing a critical dialogue of harmful social norms, providing emotional and material support and by forming positive action plans and solidarity to mobilise them [21]. Conversely, they can also entrench negative norms and facilitate dissemination of false information. It is unclear how community membership may have differential impact on FSW and NSW in enhancing/attenuating service uptake.

Influence of individual and interpersonal level factors on uptake of health services

A complex interplay of biological and behavioural factors drive differences in HIV risk in FSW compared to NSW which in turn will influence their respective need for and exposure to HIV services. Unsurprisingly, awareness and knowledge of HIV services have been identified as a critical component to encouraging service uptake. Batona et al found FSW who had previously undergone HIV counselling and testing (HTC) were more likely to become engaged with services a second time and displayed less resistance to testing and initiation in the treatment cascade [24]. A synergistic and reciprocal relationship exists between STIs (such as HSV-2 and bacterial vaginosis) and HIV such that acquisition of one can facilitate acquisition and transmission of the other [25–27]. Unprotected sex with multiple sexual partners puts FSWs at greater risk than NSW of symptomatic STIs and HIV. Consequently, FSW may be more likely to access services than NSW to resolve these health concerns, not least because ill-health may cause loss of earnings. Relatedly, greater perceived risk among FSWs may drive higher rates of health service uptake [1,8].

For many women worldwide, initial exposure to HIV testing is via antenatal care services (ANC). We might expect lower exposure to HIV testing through ANC for FSW for a couple of reasons. First, since FSW have higher prevalence of HIV than NSW and HIV reduces fertility [12] , we might expect incidence of pregnancy among FSW to be lower. Second, pregnancy represents an opportunity cost for FSW (loss of earnings) and so they may be more likely to take steps to avoid it (e.g. hormonal contraception).

A systematic reivew of barriers and faciliators to accessing ART care globally found a number of individual-level barriers were influential including younger age, lower education level, longer distances from clinics, higher transport costs, as well as inability to take time off work and other time constraints [6]. In a previous study of sex workers in Zimbabwe, we found FSW

were significantly higher educated, older and were more likely to live in urban areas where facilities are more closely available. Differences in wealth of FSW compared to NSW may mean they have different capacities to pay for healthcare-related costs [28]. Conversely, if sex workers are less likely to live with children (either because they have no children or have travelled to work), FSW may have lower childcare related expenditures than NSW which may mean greater disposable income for healthcare-related expenditures [29].

High rates of mental health disorders in FSW have been attributed to discrimination and social rejection as a result of their work, higher rates of violence (physical, sexual and emotional) from clients, non-paying intimate partners, police and economic pressures to support dependents [5]. Data from Zimbabwe suggest FSW have higher levels of mental ill-health than NSW and that mental ill-health is linked to poorer adherence to ART [30]. In addition to the fear of HIV positive diagnosis, disclosure as HIV positive connotes additional negative consequences for FSW, it being undesirable for potential clients and potentially resulting in a loss of earnings.

In frameworks for HIV risk, interpersonal factors include frequency and type of sexual relationships and the negotiation of condom use therein [2,5]. Intimate male partners can effectively control their female spouse’s access to HIV treatment, causing substantial treatment delays [6,13] intimate partner violence has been linked to lower ART use and viral load suppression [31] and for FSW having an intimate partnership can present a significant obstacle to achieving viral suppression [32]. If NSW are more likely to have an intimate male partners than FSW, we might therefore expect uptake among NSW to be more affected by the influence of partners.

The impact of interpersonal factors on health-service uptake need not relate solely to sexual relationships but may also be driven through social relationships. In HIV prevention, use of peers has had important beneficial impact in enhancing knowledge of HIV risks, encouraging condom use and reducing HIV/STI infections [33,34]. Use of peers to encourage uptake of HIV care is less well studied. In India, a requirement to take a “buddy” or family member before treatment was issued prevented FSW and MSM from accessing services [7] and peer-led interventions may be limited if the social environment is not health-enabling [35]. Nevertheless, peers have been used with some success in preventing mother-to-child transmission of HIV [36] and near-peers (health workers with shared cultural background with clients) have been used in the US to significantly increase viral load suppression by helping patients navigate non-integrated HIV care systems [37]. We hypothesis peer use of HIV care as a potential factor to encourage service access.

B. Shona Symptom Questionnaire

Mental health was assessed using the Shona Symptom Questionnaire (SSQ), a 14-item questionnaire of ‘yes or ‘no’ questions, developed and validated in Zimbabwe in 1997 with the aid of mental healthcare providers [38]. The SSQ quantifies psychological distress as a function of somatic and psychological experiences over the week prior to interview. Using validated cut-points indicating levels of psychological distress [38], a dichotomous variable (0/1) was created with individuals with an SSQ score ≥7 (coded 1) as currently experiencing psychological distress [30].

C. HIV testing amongst HIV+ women

Table S1: Factors contributing to the difference in uptake of HIV testing between HIV-positive FSW and NSW, Manicaland Zimbabwe, 2009-2011

				Bivariate		Socio-demographic		Socio-demographic Sexwork		Intermediate Determinants		Intermediate Determinants Sexwork		Full Model	
				**AOR**	**95% CI**	**AOR**	**95% CI**	**AOR**	**95% CI**	**AOR**	**95% CI**	**AOR**	**95% CI**	**AOR**	**95% CI**
**Female Sex Work**															
Sex Work	NSW	379	(83.48%)	1	-	-	-	1	-	-	-	1	-	1	-
	FSW	75	(16.52%)	1.51	0.85-2.70	-	-	1.83	1.00-3.37	-	-	1.02	0.51,2.05	1.14	0.56,2.35
**Socio-demographic**															
Age-group															
	19-29	96	(21.15%)	1	-	-	-	-	-	-	-	-	-	-	-
	30-39	178	(39.21%)	1.06	0.64-1.75	-	-	-	-	-	-	-	-	-	-
	40-49	126	(27.75%)	1.31	0.75-2.29	-	-	-	-	-	-	-	-	-	-
	50-58	54	(11.89%)	0.82	0.43-1.55	-	-	-	-	-	-	-	-	-	-
Marital status															
	Never married	139	(30.62%)	0.52	0.18-1.52	-	-	-	-	-	-	-	-	-	-
	Married	9	(1.98%)	1	-	-	-	-	-	-	-	-	-	-	-
	Divorced or separated	237	(52.2%)	0.90	0.53-1.54	-	-	-	-	-	-	-	-	-	-
	Widowed	69	(15.2%)	1.45	0.89-2.38	-	-	-	-	-	-	-	-	-	-
Religion															
	Christian	238	(52.42%)	1	-	1	-	1	-	-	-	-	-	1	-
	Spiritual	156	(34.36%)	0.93	0.60-1.42	1.00	0.65-1.55	1.02	0.66-1.58	-	-	-	-	1.09	0.64,1.85
	Other	48	(10.57%)	0.69	0.38-1.24	0.77	0.42-1.42	0.76	0.41-1.39	-	-	-	-	0.79	0.38,1.63
	None	12	(2.64%)	0.42	0.16-1.08	0.50	0.19-1.33	0.41	0.15-1.12	-	-	-	-	0.33+	0.10,1.07

Socio-economic status															
	First (poorest) tercile	284	(62.56%)	1	-	-	-	-	-	-	-	-	-	-	-
	Second tercile	114	(25.11%)	1.31	0.82-2.10	-	-	-	-	-	-	-	-	-	-
	Third tercile	48	(10.57%)	1.08	0.58-2.02	-	-	-	-	-	-	-	-	-	-
Residential area															
	Town	143	(31.5%)	1	-	1	-	1	-	-	-	-	-	1	-
	Agricultural estate	125	(27.53%)	1.02	0.64-1.64	1.02	0.63-1.65	1.06	0.65-1.73	-	-	-	-	1.53	0.84,2.79
	Roadside settlement	79	(17.4%)	1.61	0.93-2.79	1.46	0.83-2.54	1.52	0.87-2.67	-	-	-	-	2.25*	1.14,4.42
	Subsistence farming village	107	(23.57%)	1.20	0.69-2.10	1.09	0.61-1.93	1.13	0.64-2.01	-	-	-	-	1.47	0.70,3.10
Education															
	Primary or none	178	(39.21%)	1	-	1	-	1	-	-	-	-	-	1	-
	Secondary or higher	276	(60.79%)	1.75	1.14-2.71	1.63	1.04-2.53	1.66	1.06-2.58	-	-	-	-	1.47	0.85,2.55
Children alive															
	None	102	(22.47%)	1	-	-	-	-	-	-	-	-	-	-	-
	1	140	(30.84%)	1.15	0.68-1.96	-	-	-	-	-	-	-	-	-	-
	2	106	(23.35%)	0.91	0.53-1.57	-	-	-	-	-	-	-	-	-	-
	3	60	(13.22%)	0.95	0.50-1.82	-	-	-	-	-	-	-	-	-	-
	4+	46	(10.13%)	1.65	0.73-3.73	-	-	-	-	-	-	-	-	-	-
**Intermediate Determinants**															
Knowledge about HIV risks															
	Good	395	(87.%)	1.28	0.75,2.18	-	-	-	-	-	-	-	-	-	-
	Poor	59	(13.%)	1	-	-	-	-	-	-	-	-	-	-	-
Risk perception for HIV infection															
	Own high-risk behaviour	55	(12.11%)	4.53***	2.06,9.93	-	-	-	-	2.49*	1.06,5.84	2.47*	1.01,6.02	3.13*	1.22,8.08
	Partner(s)' high-risk behaviour	136	(29.96%)	22.46***	8.05,62.68	-	-	-	-	16.57***	5.28,51.93	16.58***	5.29,51.98	18.51***	5.77,59.35
	Other reasons	66	(14.54%)	4.78***	2.29,10.01	-	-	-	-	3.22**	1.47,7.07	3.22**	1.46,7.07	3.57**	1.59,7.99

	None	197	(43.39%)	1	-	-	-	-	-	1	-	1	-	1	-
Knowing PLHIV / died from HIV															
	0	53	(11.67%)	1	-	-	-	-	-	1	-	1	-	1	-
	1 - 2	85	(18.72%)	2.23**	1.21,4.09	-	-	-	-	1.70	0.82,3.52	1.7	0.82,3.52	1.78	0.84,3.75
	3 - 4	74	(16.3%)	2.69**	1.39,5.22	-	-	-	-	1.79	0.82,3.88	1.79	0.82,3.88	1.73	0.79,3.82
	5 - 6	85	(18.72%)	3.15***	1.63,6.08	-	-	-	-	2.01+	0.93,4.34	2.01+	0.93,4.34	2.17+	0.98,4.80
	7	157	(34.58%)	2.72***	1.56,4.73	-	-	-	-	1.18	0.60,2.33	1.18	0.60,2.33	1.18	0.59,2.37
STD symptoms in last 12 months															
	Yes	86	(18.94%)	1.80*	1.02,3.20	-	-	-	-	0.63	0.31,1.25	0.63	0.31,1.26	0.67	0.33,1.36
	No	368	(81.06%)	1	-	-	-	-	-	1	-	1	-	1	-
Sickness in last 12 months															
	HIV-related illness	95	(20.93%)	0.06***	0.02,0.21	-	-	-	-	0.14**	0.04,0.49	0.14**	0.04,0.49	0.12**	0.03,0.45
	Other illness	165	(36.34%)	0.10***	0.03,0.33	-	-	-	-	0.22*	0.06,0.78	0.22*	0.06,0.79	0.20*	0.06,0.74
	None	193	(42.51%)	1	-	-	-	-	-	1	-	1	-	1	-
Psychological distress															
	Yes	366	(80.62%)	1.23	0.74,2.05	-	-	-	-	-	-	-	-	-	-
	No	88	(19.38%)	1	-	-	-	-	-	-	-	-	-	-	-
Pregnancies in last 3 years															
	One or more	335	(73.79%)	1.29	0.78,2.13	-	-	-	-	-	-	-	-	-	-
	None	119	(26.21%)	1	-	-	-	-	-	-	-	-	-	-	-
Travel time to HIV testing facility															
	<30 mins	106	(23.35%)	1	1.00,1.00	-	-	-	-	1	-	1	-	1	-
	30 - 59 mins	113	(24.89%)	0.64	0.35,1.16	-	-	-	-	0.58	0.29,1.12	0.58	0.29,1.13	0.50+	0.25,1.01
	60 - 89 mins	91	(20.04%)	0.57	0.30,1.05	-	-	-	-	0.53+	0.26,1.06	0.53+	0.26,1.06	0.39*	0.18,0.84
	90 mins	142	(31.28%)	0.9	0.49,1.66	-	-	-	-	0.81	0.41,1.61	0.82	0.41,1.62	0.65	0.31,1.33
	Uncertain	2	(.44%)	0.02***	0.00,0.08	-	-	-	-	0.03***	0.01,0.13	0.03***	0.01,0.13	0.02***	0.00,0.10
Knowledge of ART															
	Yes	345	(75.99%)	3.47***	2.33,5.16	-	-	-	-	1.21	0.74,1.98	1.21	0.74,1.98	1.07	0.63,1.82

	No	108	(23.79%)	1	-	-	-	-	-	1	-	1	-	1	-
Stigma and discrimination (in the community)															
	Yes	90	(19.82%)	1.27	0.77,2.11	-	-	-	-	-	-	-	-	-	-
	No	363	(79.96%)	1	-	-	-	-	-	-	-	-	-	-	-

Supplementary References

1. Albarracín D, Gillette JC, Earl AN, Glasman LR, Durantini MR, Ho M-H. A Test of Major Assumptions About Behavior Change: A Comprehensive Look at the Effects of Passive and Active HIV-Prevention Interventions Since the Beginning of the Epidemic. Psychol Bull. 2005;131: 856–897. doi:10.1037/0033-2909.131.6.856

2. Shannon K, Goldenberg SM, Deering KN, Strathdee SA. HIV infection among female sex workers in concentrated and high prevalence epidemics: why a structural determinants framework is needed. Curr Opin HIV AIDS. 2014;9: 174–182. doi:10.1097/COH.0000000000000042

3. Kaufman MR, Cornish F, Zimmerman RS, Johnson BT. Health Behavior Change Models for HIV Prevention and AIDS Care: Practical Recommendations for a Multi-Level Approach. J Acquir Immune Defic Syndr 1999. 2014;66: S250–S258. doi:10.1097/QAI.0000000000000236

4. Johnson BT, Redding CA, DiClemente RJ, Mustanski BS, Dodge BM, Sheeran P, et al. A Network-Individual-Resource Model for HIV Prevention. AIDS Behav. 2010;14: 204–221. doi:10.1007/s10461-010-9803-z

5. Shannon K, Strathdee SA, Goldenberg SM, Duff P, Mwangi P, Rusakova M, et al. Global epidemiology of HIV among female sex workers: influence of structural determinants. The Lancet. 2015;385: 55–71. doi:10.1016/S0140-6736(14)60931-4

6. Govindasamy D, Ford N, Kranzer K. Risk factors, barriers and facilitators for linkage to antiretroviral therapy care: a systematic review. AIDS Lond Engl. 2012;26: 2059–2067. doi:10.1097/QAD.0b013e3283578b9b

7. Beattie TSH, Bhattacharjee P, Suresh M, Isac S, Ramesh BM, Moses S. Personal, interpersonal and structural challenges to accessing HIV testing, treatment and care services among female sex workers, men who have sex with men and transgenders in Karnataka state, South India. J Epidemiol Community Health. 2012;66 Suppl 2: ii42-48. doi:10.1136/jech-2011-200475

8. Desmond N, Allen CF, Clift S, Justine B, Mzugu J, Plummer ML, et al. A typology of groups at risk of HIV/STI in a gold mining town in north-western Tanzania. Soc Sci Med 1982. 2005;60: 1739–1749. doi:10.1016/j.socscimed.2004.08.027

9. Camlin CS, Kwena ZA, Dworkin SL, Cohen CR, Bukusi EA. “She mixes her business”: HIV transmission and acquisition risks among female migrants in western Kenya. Soc Sci Med 1982. 2014;102: 146–156. doi:10.1016/j.socscimed.2013.11.004

10. McGrath N, Eaton JW, Newell M-L, Hosegood V. Migration, sexual behaviour, and HIV risk: a general population cohort in rural South Africa. Lancet HIV. 2015;2: e252–e259. doi:10.1016/S2352-3018(15)00045-4

11. Rai T, Lambert HS, Ward H. Complex routes into HIV care for migrant workers: a qualitative study from north India. AIDS Care. 2015;27: 1418–1423. doi:10.1080/09540121.2015.1114988

12. Baral S, Beyrer C, Muessig K, Poteat T, Wirtz AL, Decker MR, et al. Burden of HIV among female sex workers in low-income and middle-income countries: a systematic review and meta-analysis. Lancet Infect Dis. 2012;12: 538–549. doi:10.1016/S1473-3099(12)70066-X

13. NSWP. Stigma and Discrimination Experienced by Sex Workers Living with HIV [Internet]. 2015 Dec. Available: http://www.nswp.org/resource/stigma-and-discrimination-experienced-sex-workers-living-hiv

14. Lim S, Peitzmeier S, Cange C, Papworth E, LeBreton M, Tamoufe U, et al. Violence against female sex workers in Cameroon: accounts of violence, harm reduction, and potential solutions. J Acquir Immune Defic Syndr 1999. 2015;68 Suppl 2: S241-247. doi:10.1097/QAI.0000000000000440

15. Tounkara FK, Diabaté S, Guédou FA, Ahoussinou C, Kintin F, Zannou DM, et al. Violence, condom breakage and HIV infection among female sex workers in Benin, West Africa. Sex Transm Dis. 2014;41: 312–318. doi:10.1097/OLQ.0000000000000114

16. O'Brien S, Broom A. Gender, culture and changing attitudes: experiences of HIV in Zimbabwe. Cult Health Sex. 2013;15: 583–597. doi:10.1080/13691058.2013.776111

17. Elmes J, Skovdal M. A reconfiguration of the sex trade: How social and structural changes in eastern Zimbabwe left women involved in sex work and transactional sex more vulnerable. PLOS ONE. (accepted).

18. Mtetwa S, Busza J, Chidiya S, Mungofa S, Cowan F. "You are wasting our drugs": health service barriers to HIV treatment for sex workers in Zimbabwe. BMC Public Health. 2013;13: 698. doi:10.1186/1471-2458-13-698

19. Scorgie F, Nakato D, Harper E, Richter M, Maseko S, Nare P, et al. "We are despised in the hospitals": sex workers' experiences of accessing health care in four African countries. Cult Health Sex. 2013;15: 450–465. doi:10.1080/13691058.2012.763187

20. Govindasamy D, Meghij J, Negussi EK, Baggaley RC, Ford N, Kranzer K. Interventions to improve or facilitate linkage to or retention in pre-ART (HIV) care and initiation of ART in low- and middle-income settings – a systematic review. J Int AIDS Soc. 2014;17. doi:10.7448/IAS.17.1.19032

21. Campbell C, Scott K, Nhamo M, Nyamukapa C, Madanhire C, Skovdal M, et al. Social capital and HIV Competent Communities: The role of community groups in managing HIV/AIDS in rural Zimbabwe. AIDS Care. 2013;25: S114–S122. doi:10.1080/09540121.2012.748170

22. Campbell C, Cornish F. How Can Community Health Programmes Build Enabling Environments for Transformative Communication? Experiences from India and South Africa. AIDS Behav. 2012;16: 847–857. doi:10.1007/s10461-011-9966-2

23. Kerrigan DL, Fonner VA, Stromdahl S, Kennedy CE. Community empowerment among female sex workers is an effective HIV prevention intervention: a systematic review of the peer-reviewed evidence from low- and middle-income countries. AIDS Behav. 2013;17: 1926–1940. doi:10.1007/s10461-013-0458-4

24. Batona G, Gagnon M-P, Simonyan DA, Guedou FA, Alary M. Understanding the intention to undergo regular HIV testing among female sex workers in Benin: a key issue for entry into HIV care. J Acquir Immune Defic Syndr 1999. 2015;68 Suppl 2: S206-212. doi:10.1097/QAI.0000000000000452

25. Siziya S, Marowa E, Mbengeranwa L, Latiff A. Sexually transmitted diseases in Zimbabwe: A qualitative analysis of factors associated with choice of a health care facility. Afr Health Sci. 2005;5: 114–118.

26. Freeman EE, Weiss HA, Glynn JR, Cross PL, Whitworth JA, Hayes RJ. Herpes simplex virus 2 infection increases HIV acquisition in men and women: systematic review and meta-analysis of longitudinal studies. AIDS Lond Engl. 2006;20: 73–83.

27. Atashili J, Poole C, Ndumbe PM, Adimora AA, Smith JS. Bacterial vaginosis and HIV acquisition: A meta-analysis of published studies. AIDS Lond Engl. 2008;22: 1493–1501. doi:10.1097/QAD.0b013e3283021a37

28. Elmes J, Nhongo K, Ward H, Hallett T, Nyamukapa C, White PJ, et al. The Price of Sex: Condom Use and the Determinants of the Price of Sex Among Female Sex Workers in Eastern Zimbabwe. J Infect Dis. 2014;210: S569–S578. doi:10.1093/infdis/jiu493

29. Goh CC, Kang SJ, Sawada Y. How did Korean households cope with negative shocks from the financial crisis? J Asian Econ. 2005;16: 239–254. doi:10.1016/j.asieco.2005.01.006

30. Tlhajoane M, Eaton JW, Takaruza A, Rhead R, Maswera R, Schur N, et al. Prevalence and Associations of Psychological Distress, HIV Infection and HIV Care Service Utilization in East Zimbabwe. AIDS Behav. 2017; 1–11. doi:10.1007/s10461-017-1705-x

31. Hatcher A, Smout E, Turan J, Christofides N, Stockl H. Intimate partner violence and engagement in HIV care and treatment among women: a systematic review and meta-analysis. AIDS. 2015;

32. Duff P, Goldenberg S, Deering K, Montaner J, Nguyen P, Dobrer S, et al. Barriers to Viral Suppression Among Female Sex Workers: Role of Structural and Intimate Partner Dynamics. JAIDS J Acquir Immune Defic Syndr. 2016;73. Available: http://journals.lww.com/jaids/Fulltext/2016/09010/Barriers_to_Viral_Suppression_Among_Female_Sex.12.aspx

33. Shahmanesh M, Patel V, Mabey D, Cowan F. Effectiveness of interventions for the prevention of HIV and other sexually transmitted infections in female sex workers in resource poor setting: a systematic review. Trop Med Int Health. 2008;13: 659–679. doi:10.1111/j.1365-3156.2008.02040.x

34. Medley A, Kennedy C, O’Reilly K, Sweat M. Effectiveness of Peer Education Interventions for HIV Prevention in Developing Countries: A Systematic Review and Meta-Analysis. AIDS Educ Prev. 2009;21: 181–206. doi:10.1521/aeap.2009.21.3.181

35. Campbell C, Cornish F. Towards a “fourth generation” of approaches to HIV/AIDS management: creating contexts for effective community mobilisation. AIDS Care. 2010;22: 1569–1579. doi:10.1080/09540121.2010.525812

36. Busza J, Walker D, Hairston A, Gable A, Pitter C, Lee S, et al. Community-based approaches for prevention of mother to child transmission in resource-poor settings: a social ecological review. J Int AIDS Soc Vol 15 Suppl 2 2012. 2012; Available: http://www.jiasociety.org/jias/index.php/jias/article/view/17373/688

37. Mugavero MJ, Amico KR, Horn T, Thompson MA. The State of Engagement in HIV Care in the United States: From Cascade to Continuum to Control. Clin Infect Dis. 2013;57: 1164–1171. doi:10.1093/cid/cit420

38. Patel V, Simunyu E, Gwanzura F, Lewis G, Mann A. The Shona Symptom Questionnaire: the development of an indigenous measure of common mental disorders in Harare. Acta Psychiatr Scand. 1997;95: 469–475.
