# Supplementary material for: Do female sex workers have lower uptake of HIV treatment services than non-sex workers? A cross-sectional study from east Zimbabwe
Source: BMJ Open. 2018 Feb 28;8(2):e018751. doi: 10.1136/bmjopen-2017-018751 (PMC5855339; doi:10.1136/bmjopen-2017-018751)
Supplement: Author's manuscript [file bmjopen-2017-018751.draft_revisions.pdf]

BMJ Open

BMJ Open is committed to open peer review. As part of this commitment we make the peer review history of every article we publish publicly available.

When an article is published we post the peer reviewers' comments and the authors' responses online. We also post the versions of the paper that were used during peer review. These are the versions that the peer review comments apply to.

The versions of the paper that follow are the versions that were submitted during the peer review process. They are not the versions of record or the final published versions. They should not be cited or distributed as the published version of this manuscript.

BMJ Open is an open access journal and the full, final, typeset and author-corrected version of record of the manuscript is available on our site with no access controls, subscription charges or pay-per-view fees (http://bmjopen.bmj.com).

If you have any questions on BMJ Open's open peer review process please email editorial.bmjopen@bmj.com

BMJ Open

Do Female Sex Workers have Lower Uptake of HIV Treatment Services than Non-Sex-Workers? A Case Study from East Zimbabwe

Journal:	*BMJ Open*
Manuscript ID	bmjopen-2017-018751
Article Type:	Research
Date Submitted by the Author:	21-Jul-2017
Complete List of Authors:	Rhead, Rebecca; Imperial College London School of Public Health,<br>Elmes, Jocelyn; Imperial College London Department of Infectious Disease Epidemiology<br>Otobo, Eloghene<br>Nhongo, Kundai<br>Takaruza, Albert<br>White, Peter; Imperial College London, MRC Centre for Outbreak Analysis and Modelling and NIHR Health Protection Research Unit in Modelling Methodology, Department of Infectious Disease Epidemiology; Public Health England, Modelling & Economics Unit, National Infection Service, Public Health England<br>Nyamukapa, Constance; Biomedical Research and Training Institute, Manicaland Centre for Public Health Research; Imperial College London<br>Gregson, Simon; Imperial College London, Infectious Disease Epidemiology
**Primary Subject Heading**:	HIV/AIDS
Secondary Subject Heading:	Research methods, Public health, Infectious diseases, Epidemiology
Keywords:	HIV & AIDS < INFECTIOUS DISEASES, Epidemiology < INFECTIOUS DISEASES, Public health < INFECTIOUS DISEASES

SCHOLARONE™
Manuscripts

Do Female Sex Workers have Lower Uptake of HIV Treatment Services than Non-Sex-Workers? A Case Study from East Zimbabwe

Rebecca RHEAD[a*] and Jocelyn ELMES[a*], Eloghene OTOBO[a], Kundai NHONGO[b], Albert TAKARUZA[b], Peter J WHITE[a,c,d], Constance NYAMUKAPA[a,b], Simon GREGSON[a,b]

[a]Department of Infectious Disease Epidemiology, Imperial College London School of Public Health, London, United Kingdom,
[b]Biomedical Research and Training Institute, Harare, Zimbabwe.
[c]MRC Centre for Outbreak Analysis and Modelling and NIHR Health Protection Research Unit in Modelling Methodology, Imperial College London School of Public Health, London, United Kingdom
[d]Modelling and Economics Unit, National Infection Service, Public Health England, London, United Kingdom

*These authors have contributed equally to the work.

Author for correspondence and reprint requests: Rebecca Rhead, Department of Infectious Disease Epidemiology, Imperial College London School of Public Health, London, United Kingdom. E-mail: r.rhead@imperial.ac.uk

Source of financial support: Wellcome Trust, MRC, NIHR

Short title: HTC and ART uptake in Zimbabwean sex workers

Key words: Female sex workers, HIV prevalence, HIV treatment cascade, determinants, Zimbabwe.

Word Count: 3512

For peer review only - http://bmjopen.bmj.com/site/about/guidelines.xhtml

Abstract

Objective: High antiretroviral treatment (ART) coverage in female sex workers (FSW) is vital for equity and to reduce HIV transmission in the general population. We compare and investigate HIV treatment cascades for FSW and non-sex-workers (NSW) in Manicaland province, Zimbabwe.

Methods: Data from a household survey conducted in 2009-2011 and a parallel snowball sample survey of FSW were matched using probability methods to reduce under-reporting of FSWs. HIV treatment cascades – HIV diagnosis, ART initiation, and ART adherence as a proxy for viral load suppression – were constructed and compared for FSW (n=174) and NSW (n=2,555). Socio-demographic characteristics and intermediate determinants that might explain differences in service uptake between FSW and NSW were identified *a priori* in a theoretical framework and tested using logistic regression.

Results: HIV prevalence was higher in FSW than in NSW (52.6% *versus* 19.8%; age-adjusted odds ratio [AOR] 4.0; 95% CI 2.9-5.5). In HIV-positive women, FSW were more likely to have been diagnosed (58.2% *versus* 42.6%; AOR=1.62; 1.02-2.59) and to have initiated ART (84.9% *versus* 64.0%; AOR=2.33; 1.03-5.28). No difference was found for ART adherence (91.1% *versus* 90.5%; p=0.9). FSW's greater uptake of HIV treatment services became non-significant after adjusting for intermediate factors including HIV knowledge and risk perception, travel time to services, physical and mental health, and recent pregnancy.

Conclusion: FSW do not have lower uptake of HIV treatment services than NSW in east Zimbabwe. However, ART coverage was low in all women at the time of the survey.

For peer review only - http://bmjopen.bmj.com/site/about/guidelines.xhtml

Strengths and Limitations of this Study

1. We provide novel insight into differential uptake of HIV treatment services for FSW and NSW in Manicaland province, Zimbabwe, and the personal, social and structural factors associated with these inequalities.
2. We use data taken from a Manicaland household survey and a parallel snowball sample survey of FSW, thus drawing on the strengths of population surveys and targeted approaches for hard-to-reach populations.
3. Our study is unique in that it compares uptake of HIV testing and ART in representative samples of FSW and NSW from the same population - we are unaware of previous studies which have done this.
4. A limitation of our study is that our data was gathered between 2009-2011.

For peer review only

Introduction

Achieving high antiretroviral treatment (ART) uptake for PLHIV is key to ending the HIV epidemic worldwide [1–3]. Though UNAIDS has set ambitious "90-90-90" targets for the HIV care cascade (i.e. HIV diagnosis, ART initiation and ART adherence – as a proxy for viral load suppression) [4], these are national-level targets, and it is necessary to consider how they can be implemented for key populations such as female sex workers (FSW) – women who engage in commercial sex work or who exchange sex for goods or services[5]. HIV prevalence among FSW in sub-Saharan Africa is often 10–20 times higher than in women in the general population [6]. With high rates of sexual partner change and inconsistent condom use in commercial sex, sex work may contribute substantially to population-level HIV incidence even in high prevalence epidemics in the region [7]. Therefore reaching and exceeding UNAIDS targets amongst FSW should be a primary objective for all national HIV control programmes [8].

Whilst stigma, marginalization, and abuse of human rights have all been highlighted as significant barriers that can prevent FSW from accessing HIV testing and treatment services [9], relatively few studies exist on HIV treatment cascades amongst representative samples of FSW. These include a study by Cowan and colleagues (2013) in three urban sites in Zimbabwe (Victoria Falls, Hwange and Mutare) where 50-70% were seropositive, of whom only 50% had been diagnosed. Of those diagnosed, 50-70% had been initiated onto treatment, but due to the low rate of diagnosis, only 25-35% of *all* seropositive FSW in the study had received ART [10]. Still, very little is known about FSW in more rural settings, or about how FSW's use of HIV services compares with that of non-sex-workers (NSW) living in the same areas. A further unknown is the extent to which differences in

For peer review only - http://bmjopen.bmj.com/site/about/guidelines.xhtml

heath service uptake between FSW and NSW reflect largely psychosocial factors resulting from involvement in sex work (e.g. personal risk perception) as distinct from background socio-demographic factors associated with being involved in sex work in the first place.

This study has the following aims: 1) to construct and compare HIV treatment cascades for FSW and NSW in a common, rural population; 2) to identify the background socio-demographic characteristics associated with involvement in commercial sex work in this population; and 3) to identify the intermediate factors that might explain differences in health service uptake (testing and treatment) between FSW and NSW. To achieve these aims, we develop a new theoretical framework and test hypothesised determinants based on this framework using a unique data set which combines data from a general population household survey in four locations in Manicaland province, east Zimbabwe, with data from a parallel study of local FSW conducted in the same locations using snowball sampling.

For peer review only

Methods

Theoretical framework

Influenced by Boerma and Weir's proximate determinants model of HIV infection and mortality [11,12] and structural determinants frameworks of HIV among sex workers [13], we developed a theoretical framework to explain the roles that involvement in sex work and its consequences can play in mediating associations between underlying socio-demographic characteristics and use of HIV testing and treatment services (Figure 1 and supplementary material). It is hypothesized that, within any given socio-cultural context, underlying socio-demographic characteristics contribute to whether or not a woman engages in sex work which may, in turn, alter her pattern of use of HIV services. In the framework, sex work is hypothesized to influence use of HIV services primarily through its effects on intermediate determinants that exist in four domains: personal, interpersonal, social and structural.

Figure 1: Theoretical framework

Data

Data for this study were taken from the Manicaland HIV/STD Prevention Project (Manicaland study) [14] and the Manicaland Women at Risk Study (WR study) [15]. The Manicaland study is an open-cohort general-population survey which examines the dynamics of HIV transmission and its impact in 12 sites in Manicaland province in eastern Zimbabwe (*http://www.manicalandhivproject.org/*). These sites represent four of the main socio-economic strata in Manicaland: small towns, agricultural estates, roadside trading centres, and subsistence farming villages. Topics covered in individual interviews included

For peer review only - http://bmjopen.bmj.com/site/about/guidelines.xhtml

socio-economic characteristics, sexual behaviour, psychosocial characteristics, and use of HIV testing and treatment services. Participants were also requested to provide a dried blood sample (DBS) for HIV sero-testing. The data used in this analysis were taken from the 5th round of the Manicaland survey (October 2009 - July 2011) and were restricted to the four sites (one in each socio-economic stratum) also covered by the WR study.

The data from the Manicaland study were linked with data from the WR study, a parallel targeted cohort study conducted to identify women at heightened risk of HIV infection through exchange of sex (including sex work), to enhance detection of FSW and to permit comparison of HIV treatment cascades between FSW and NSW from with a common population. The WR study is a research project, conducted in four of the same sites covered in the Manicaland study, which aimed to explore the sexual behaviours of women at heightened risk of HIV infection (*http://www.manicalandhivproject.org/women-at-risk.html*). Data for the WR study were collected between March 2010 and July 2011 using a combination of PLACE (Priorities for Local AIDS Control Effort, a form of location based sampling) [16] and snowball sampling [17] methods. Data collection procedures have been described in detail elsewhere [18] but are summarised here. PLACE involves sampling locations of known sex work activity. An inventory of locations was created based on discussions with community members. Since only a small number of venues were identified, all venues were sampled. To capture exchange sex outside of specific venues, the population was sampled using a modified respondent-driven sampling approach [19]. Seeds were selected to represent the diversity of those involved in exchange sex. These seeds then recruited up to three peers that met broad eligibility criteria (women aged 18+ who had ever exchanged sex for money, goods, or favours) and were

For peer review only - http://bmjopen.bmj.com/site/about/guidelines.xhtml

compensated with one bar of laundry soap per respondent referred and invited to interview.

FSW who participated in both the Manicaland and WR study were requested to provide permission to link their data across both projects. Data for consenting participants were linked via probabilistic matching based on participant name, date of birth, and village name. Prior ethical approval for the Manicaland study (with the WR study included as a sub-study) was obtained from the Medical Research Council of Zimbabwe (MRCZ/A/681) and the Imperial College Research Ethics Committee (ICREC_9_3_13).

Study variables

Female sex worker: The Manicaland and WR studies contained identical indicators of sex work. Informed by prior qualitative work within study communities [20] and in line with UNAIDS definitions [21], participants in each study were considered to be FSW if they: a) self-identified as a sex worker or prostitute; b) had ever gone to bars/beer halls to meet clients; or c) had exchanged sex for money/goods. To improve comparability between FSW and NSW other women in the WR sites within the Manicaland study were matched to the same age-range as the WR study participants and treated as NSWs. Women who had never engaged in sexual intercourse were excluded from the study.

HIV treatment cascade: HIV diagnosis was defined as the percentage of all HIV-positive participants (based on HIV tests done in the Manicaland study) who reported ever having been tested and having collected their results and received a positive result at their most recent HIV test. ART initiation was defined as the percentage of HIV-positive participants who knew their status (denominator) and also reported taking drugs “that stop HIV from

For peer review only - http://bmjopen.bmj.com/site/about/guidelines.xhtml

causing AIDS" (numerator). ART adherence was used as a further indicator of HIV service use and as a proxy for viral load suppression. HIV-positive participants who reported ever having started ART were included in the denominator; those who reported never having stopped or forgotten to take their medication and who reported taking ARVs regularly were included in the numerator.

Health service uptake: Two measures of health service engagement were considered as dependent variables in our regression analyses: 1) uptake of HIV testing; and 2) uptake of ART. Uptake of HIV testing was defined as ever having had an HIV test and collected the result. Uptake of treatment was measured in seropositive participants and based on reports of having taking drugs "that stop HIV from causing AIDS".

Socio-demographic characteristics: Age, marital status, socio-economic status, religion, area of residence, education level, and number of living children were considered as potential underlying determinants of involvement in sex work and use of HIV services (Figure 1). For socio-economic status (SES), we used a continuous combined measure of sellable and non-sellable assets [22], divided into terciles (1=poorest → 3=richest). For religious denomination, we used Manzou's four category grouping of Manicaland churches [23].

Intermediate determinants of HIV service uptake: Personal factors potentially mediating HIV service uptake included: recent ill-health (self-reported experience of recent ill-health and whether or not this was believed to be HIV-related), self-reported symptoms of STDs, self-reported recent pregnancies (that could translate to HIV testing through uptake of PMTCT services), HIV knowledge (number of correct responses to four questions: 0-2

For peer review only - http://bmjopen.bmj.com/site/about/guidelines.xhtml

correct answers=poor knowledge, 3-4 correct answers=good knowledge), HIV risk perception (whether participants perceived they had ever been at risk of becoming infected with HIV, and if so, was it through their own risky behaviour, their partner's risky behaviour or for other reasons), awareness of treatment for HIV, and an objective mental health assessment using a locally-validated questionnaire (Shona Symptom Questionnaire, SSQ) [24] [25]. Interpersonal factors included HIV salience (number of people known by the participant who are living with HIV or who had died from AIDS) and awareness of other people using ART (individuals who were unaware of ART were combined with those unaware of anyone using ART because of small numbers). Potential social and structural influences included accessibility of HTC (or ART) services; participants aware of a health facility offering HTC (or ART) estimated the travel time to the nearest such health facility. Stigma was measured using two dichotomous variables: whether the participant was ever deterred from getting a test due to stigma or discrimination, and whether the participant felt that PLHIV faced stigma and discrimination within the community.

Travel time and stigma relating to HTC and awareness of ART were used only in the analysis of uptake of testing (i.e. not for ART uptake). Travel time to ART services was used only in the analysis of ART uptake (i.e. not for HIV testing).

Statistical analyses

The analysis consisted of several stages. First, HIV prevalence and HIV treatment cascade outcomes were calculated and compared between FSW and NSW. Second, bivariate (age-adjusted) regression models were used to explore associations between socio-demographic characteristics and involvement in sex work, and associations between sex work and

For peer review only - http://bmjopen.bmj.com/site/about/guidelines.xhtml

hypothesized intermediate determinants of uptake. Third, age-adjusted bivariate examination of associations between both socio-demographic characteristics and intermediate determinants and HIV testing and treatment was conducted to detect significant associations at $p<0.1$. Fourth, age-adjusted multivariable regression models were used to assess the association between sex work and health service uptake – before and after inclusion of socio-demographic factors and intermediate determinants of uptake (significant at $p<0.1$). All analyses were done using Stata 14.

For peer review only

Results

Identification of FSW

A total of 3402 women aged 15-59 years participated in the Manicaland study in round five in the four sites also covered by the WR study. Of these, 174 were identified as FSW; 111 were identified based on their responses to the WR study questionnaire alone, 31 were identified in both questionnaires, and 32 were identified based on their answers to the Manicaland study questionnaire alone. We excluded 135 NSW who were outside the age-range of the FSW in the study (19-58 years) and a further 538 who had not started sex. This produced a total sample of 2729 (FSW=174, 6.4%; NSW=2555, 93.6%).

HIV prevalence

HIV prevalence was significantly higher in FSW (52.6%, 95%CI 45.1%-60.0%; n/N=91/173) compared to NSW (19.8%, 18.3%-21.4%; 502/2535) (age-adjusted odds ratio [AOR] 4.0; 2.9-5.5). Study HIV laboratory test results were inconclusive for 1 FSW and 2 NSWs.

HIV treatment cascades in FSW and NSW

In HIV-positive women, diagnosis (i.e. women who were aware of their HIV-positive status) was higher in FSW (58.2%, 95%CI 47.7%-68.1%; 53/91) than in NSW (42.6%, 38.3%-47.9%; 214/502) (AOR, 1.62; 1.02-2.59; p=0.042. In HIV-negative women, FSW were not significantly more likely to have had an HIV test (81.7% in FSW; 75.3% in NSW; AOR, 1.40; 0.78-2.51; p=0.259).

For peer review only - http://bmjopen.bmj.com/site/about/guidelines.xhtml

In women diagnosed with HIV infection, initiation onto ART was higher in FSW (84.9%, 72.1%-92.4%; 45/53) than in NSW (64.2%, 57.5%-70.4%; 138/214) (AOR, 2.33; 1.03-5.28; p=0.043). No significant difference was found between FSW (91.1%, 77.9%-96.7%; 41/45) and NSW (90.5%, 84.2%-94.4%; 124/137) in self-reported adherence to ART (AOR, 1.08; 0.33-3.52; p=0.901). Overall, 49.4% (45/91) of ***all*** HIV-positive FSW reported being on and adhering to ART compared to 27.5% (139/505) of infected NSW (as shown in Figure 2b)

Figure 2a & 2b: Comparisons of HIV treatment cascades

Socio-demographic characteristics and intermediate determinants of HIV service use associated with sex work

Table 1 shows the age-adjusted bivariate associations between sociodemographic factors and sex work, and between sex work and intermediate determinants (i.e. the first two pathways in Figure 1). Sex work was most common in women aged 30-49; single, divorced and widowed women; women with no religious affiliation; women in the two poorest terciles; women living in small towns; and women with no living children. For the intermediate determinants, sex work was associated with risk perception for HIV infection (particularly through personal risky behaviours); knowing at least three people with HIV; experiencing recent HIV-related illness and STD symptoms; poor mental health; no pregnancies in the past three years; short travel times to HTC and ART facilities; having heard of ART; and reporting that HIV stigma and discrimination exist in the community.

Table 1: Associations between FSW and socio-demographic and intermediate determinants

For peer review only - http://bmjopen.bmj.com/site/about/guidelines.xhtml

Socio-demographic characteristics and intermediate determinants of HIV testing

In HIV-positive and HIV-negative women combined, FSW were more likely than NSW to have ever been tested for HIV (FSW: 81.6%, NSW: 75.3%; AOR 1.50; 95%CI 1.00-2.24). Table 2 shows the bivariate and multivariable associations of socio-demographic factors and intermediate determinants on testing. In bivariate analysis, sex work was associated with ever having been tested, as are all socio-demographic factors. All intermediate determinants with the exception of psychological distress and stigma (after testing and in the community) are also associated with testing at $p<0.1$. In multivariable analysis, the association between sex work and testing is strengthened after adjusting for socio-demographic factors, with sex work being associated with 75% increased odds of testing (AOR 1.75, 1.14-1.69). However, this association between sex work and testing disappears after also accounting for intermediate determinants (AOR 1.11, 0.69-1.81).

When the analysis was restricted to HIV-positive women (Table S1), the association between FSW and diagnosis approached statistical significance after adjusting for socio-demographic factors (AOR 1.83, 1.00-3.37; $p=0.052$).

Table 2: Associations between FSW and HIV testing

Socio-demographic characteristics and intermediate determinants of HIV treatment

Table 3 shows the bivariate and multivariable associations between socio-demographic factors and intermediate determinants and ART initiation. In bivariate analysis, sex work is associated with 164% increased odds of ART initiation (AOR 2.64, 1.16-6.00). Older age, more urban site types, having no living children, knowing people who have / had HIV, not

For peer review only - http://bmjopen.bmj.com/site/about/guidelines.xhtml

having psychological distress, and shorter travel times to ART were also associated with treatment initiation at $p<0.1$. In multivariable analysis, FSW still tended to have higher odds of ART initiation, but this association was no longer statistically significant when socio-demographic factors were accounted for (AOR 2.28, 0.97-5.39).

Table 3: Associations between FSW and ART uptake

For peer review only
For peer review only - http://bmjopen.bmj.com/site/about/guidelines.xhtml

Discussion

FSW had higher uptake of HIV testing and ART services than other women in our study areas in east Zimbabwe. For HIV testing, this advantage strengthened after accounting for differences in background socio-demographic characteristics but disappeared after further adjustment for intermediate determinants. FSW's greater knowledge about HIV and greater personal perceived risk of being HIV-positive, their better knowledge of and proximity to testing services, and their greater likelihood of perceiving HIV-related symptoms may have contributed to their higher levels of HIV testing. Differences in ART uptake between FSW and NSW was associated not by intermediate factors relating to sex work status but by their older ages (i.e. fewer aged 19-29 years) and lower numbers of living children. The reason for the link with small numbers of living children is not clear but, in *Shona* culture [26], subfertility/infertility can lead to divorce which, in turn, is associated with greater likelihood of involvement in sex work. Widowhood at young ages may be associated with early HIV infection, reduced fertility, high early child mortality and involvement in sex work. Also, low fertility and early child mortality can be markers for more advanced HIV infection [27]; thereby increasing the likelihood of meeting the eligibility criteria for ART that pertained at the time of the study (CD4<350 or World Health Organisation phases III or IV in 2009-2011). ART adherence was similar in FSW and NSW.

HIV prevalence in FSW in east Zimbabwe (52.6%) was comparable with prevalence in FSW in other southern African countries (range: 59.6-70.7%) [6]. The proportion of infected FSW who had been diagnosed (58.2%) was slightly higher than estimates for FSW in urban Zimbabwean locations (50% in 2013) [10]; whilst the proportion of those

For peer review only - http://bmjopen.bmj.com/site/about/guidelines.xhtml

diagnosed who had been started on treatment (87% *versus* 0-73%) and the proportion of those on treatment who reported adhering to ART (91% *versus* 67-100% [28] [29] [30]) were also high. We are unaware of any previous studies that have compared uptake of HIV testing and ART in representative samples of FSW and NSW from the same population. However, similar levels of ART adherence have been found in Mozambique and Benin [31] [32].

Our results suggest that several structural, interpersonal and personal factors may contribute to differences in uptake of HIV testing and ART services between and amongst FSW and NSW. As noted previously by Paulin and colleagues in a rural setting in Mozambique [33] knowledge of HTC services can affect uptake amongst women (42%). In Manicaland, FSW had better knowledge of ART and, because they lived largely in towns, were structurally advantaged over NSW, who more often lived in areas more remote from testing and treatment facilities. In terms of HIV care, these factors appear to have offset the disadvantages that FSW face from poorer mental health and greater stigma and discrimination. Poor mental health, in the form of greater psychological distress, is associated with lower ART uptake in east Zimbabwe [25]. As in many previous studies [34], we found that psychological distress was more common in sex workers. FSW in this study also reported higher levels of stigma linked to HIV than NSW; however, unlike studies elsewhere in Zimbabwe, we did not find stigma to be a significant deterrent to accessing healthcare. One reason may be that our study questionnaire did not include a measure of stigma specifically related to sex work. An interpersonal factor – not included in this study but described in previous qualitative research in Manicaland [35] – that could have reduced ART use in NSW is the dominant role of male spouses in determining women's HIV care. The effect of such unmeasured influences could be reflected in the

For peer review only - http://bmjopen.bmj.com/site/about/guidelines.xhtml

residual effect of sexwork after adjusting for sociodemographic factors and intermediate determinants.

This study utilises a unique data source that draws from the combined strengths of population surveys and chain-referral methods and allowed us to analyse a representative sample with reduced under-reporting of locally-resident FSW, and provided a rare opportunity to compare the characteristics and determinants of HIV service use for FSW and NSW from the same study areas. However, the data used were cross-sectional so we have been unable to determine the causal nature of the relationships explored in the study. Also, in comparing the HIV care cascades for FSW and NSW, we have used self-reported ART adherence as a proxy for viral suppression as biomarkers for viral load were not available. Finally, our study sites were not covered by FSW intervention programmes in Zimbabwe such as 'Sisters with a Voice' or by the SAPPHIRE trial (http://www.ceshhar.org.zw/); so it would be valuable for researchers with data from those areas to perform a comparable analysis to ours.

In east Zimbabwe between 2009 and 2011, FSW were more likely than NSW to have been tested for HIV infection and to have taken up ART. The situation may have changed subsequently (e.g. due to more widespread uptake of HTC and knowledge about ART) and could change further with the introduction of universal eligibility for ART, a comparative analysis of the later time-period to assess the impact of universal eligibility. Recent data show that a third of infected women in Zimbabwe are not yet virally suppressed [36]. Therefore, continued and enhanced efforts are needed to ensure that diagnosis and treatment in FSW keeps pace with women as a whole to ensure equity of treatment access and slow transmission. Our results suggest that this may be achieved, in part, by enhanced

For peer review only - http://bmjopen.bmj.com/site/about/guidelines.xhtml

accessibility through, for example, task shifting and more robust mental health services to improve self-efficacy among FSW and other women diagnosed with HIV infection.

For peer review only

For peer review only - http://bmjopen.bmj.com/site/about/guidelines.xhtml

Acknowledgements

Authors' contribution

JE, PJW, SG, CN and KN were involved in study concept and design. CN, JE, KN and AT acquired and curated the data. JE, RR, SG and EO were involved in the design of the analysis. RR conducted the statistical analysis supervised by SG and JE. RR, SG, JE and EO interpreted the results and drafted the article.

Data Access

Data produced by the Manicaland Project can be obtained from the project website: http://www.manicalandhivproject.org/data-access.html. Here we provide a core dataset which contains a sample of socio-demographic, sexual behaviour and HIV testing variables from all 6 rounds of the main survey, as well as data used in the production of recent academic publications. If further data is required, a data request form must be completed (available to download from our website) and submitted to s.gregson@imperial.ac.uk. If the proposal is approved, we will send a data sharing agreement which must be agreed upon before we release the requested data.

Funding Sources

SG thanks the Wellcome Trust for funding (grants: 084401/Z/07/B and 090285MA). JE thanks the Medical Research Council for her PhD funding (grant number http://www.mrc.ac.uk/index.htm) and the Wellcome Trust for postdoctoral funding (grant number: 090285/Z/09/Z; http://www.wellcome.ac.uk/). PJW thanks the MRC for Centre funding (MR/K010174/1), and the UK NIHR Health Protection Research Unit in Modelling Methodology at Imperial College London in partnership with Public Health England for funding (HPRU-2012-10080). The funders had no role in study design, data

For peer review only - http://bmjopen.bmj.com/site/about/guidelines.xhtml

collection and analysis, decision to publish, or preparation of the manuscript. The views expressed are those of the authors and not necessarily those of the Department of Health, MRC, NHS, NIHR, Public Health England, or the Wellcome Trust.

For peer review only

For peer review only - http://bmjopen.bmj.com/site/about/guidelines.xhtml

References

1. Cohen MS, Smith MK, Muessig KE, Hallett TB, Powers KA, Kashuba AD. Antiretroviral treatment of HIV-1 prevents transmission of HIV-1: where do we go from here? The Lancet. 2013;382: 1515–1524. doi:10.1016/S0140-6736(13)61998-4
2. Hontelez JAC, Lurie MN, Bärnighausen T, Bakker R, Baltussen R, Tanser F, et al. Elimination of HIV in South Africa through Expanded Access to Antiretroviral Therapy: A Model Comparison Study. PLOS Med. 2013;10: e1001534. doi:10.1371/journal.pmed.1001534
3. Collaboration TAT (ART) C. Prognostic importance of initial response in HIV-1 infected patients starting potent antiretroviral therapy: analysis of prospective studies. The Lancet. 2003;362: 679–686. doi:10.1016/S0140-6736(03)14229-8
4. HIV/AIDS JUNPo. 90–90–90 - An ambitious treatment target to help end the AIDS epidemic | UNAIDS [Internet]. 2014. Available: http://www.unaids.org/en/resources/documents/2014/90-90-90
5. Joint United Nations Programme on HIV/AIDS. UNAIDS terminology guidelines. Geneva: Joint United Nations Programme on HIV/AIDS.; 2015.
6. Baral S, Beyrer C, Muessig K, Poteat T, Wirtz AL, Decker MR, et al. Burden of HIV among female sex workers in low-income and middle-income countries: a systematic review and meta-analysis. Lancet Infect Dis. 2012;12: 538–549. doi:10.1016/S1473-3099(12)70066-X
7. Delva W, Eaton JW, Meng F, Fraser C, White RG, Vickerman P, et al. HIV Treatment as Prevention: Optimising the Impact of Expanded HIV Treatment Programmes. PLOS Med. 2012;9: e1001258. doi:10.1371/journal.pmed.1001258
8. Alary M, Lowndes CM, Van de Perre P, Béhanzin L, Batona G, Guédou FA, et al. Scale-up of combination prevention and antiretroviral therapy for female sex workers in West Africa: time for action. AIDS Lond Engl. 2013;27: 1369–1374. doi:10.1097/QAD.0b013e32835fd7bd
9. Arnott J, Crago A. Rights Not Rescue: A Report On Female, Trans and Male Sex Workers' Human Rights in Botswana, Namibia and South Africa. [Internet]. New York City; 2009. Available: https://www.opensocietyfoundations.org/sites/default/files/summary_20081114.pdf
10. Cowan FM, Mtetwa S, Davey C, Fearon E, Dirawo J, Wong-Gruenwald R, et al. Engagement with HIV Prevention Treatment and Care among Female Sex Workers in Zimbabwe: a Respondent Driven Sampling Survey. PLOS ONE. 2013;8: e77080. doi:10.1371/journal.pone.0077080
11. Boerma JT, Weir SS. Integrating Demographic and Epidemiological Approaches to Research on HIV/AIDS: The Proximate-Determinants Framework. J Infect Dis. 2005;191: S61–S67. doi:10.1086/425282

For peer review only - http://bmjopen.bmj.com/site/about/guidelines.xhtml

12. Lopman B, Nyamukapa C, Mushati P, Mupambireyi Z, Mason P, Garnett GP, et al. HIV incidence in 3 years of follow-up of a Zimbabwe cohort—1998–2000 to 2001–03: contributions of proximate and underlying determinants to transmission. Int J Epidemiol. 2008;37: 88–105. doi:10.1093/ije/dym255

13. Shannon K, Strathdee SA, Goldenberg SM, Duff P, Mwangi P, Rusakova M, et al. Global epidemiology of HIV among female sex workers: influence of structural determinants. The Lancet. 2015;385: 55–71. doi:10.1016/S0140-6736(14)60931-4

14. Gregson S, Garnett GP, Nyamukapa CA, Hallett TB, Lewis JJC, Mason PR, et al. HIV decline associated with behavior change in eastern Zimbabwe. Science. 2006;311: 664–666. doi:10.1126/science.1121054

15. Elmes J, Nhongo K, Ward H, Hallett T, Nyamukapa C, White PJ, et al. The Price of Sex: Condom Use and the Determinants of the Price of Sex Among Female Sex Workers in Eastern Zimbabwe. J Infect Dis. 2014;210: S569–S578. doi:10.1093/infdis/jiu493

16. Weir SS, Merli MG, Li J, Gandhi AD, Neely WW, Edwards JK, et al. A comparison of respondent-driven and venue-based sampling of female sex workers in Liuzhou, China. Sex Transm Infect. 2012;88: i95–i101. doi:10.1136/sextrans-2012-050638

17. Kendall C, Kerr LRFS, Gondim RC, Werneck GL, Macena RHM, Pontes MK, et al. An empirical comparison of respondent-driven sampling, time location sampling, and snowball sampling for behavioral surveillance in men who have sex with men, Fortaleza, Brazil. AIDS Behav. 2008;12: S97-104. doi:10.1007/s10461-008-9390-4

18. Elmes J. Women at Risk. In: Women at Risk Study - Manicaland Centre for Public Health [Internet]. Available: http://www.manicalandhivproject.org/women-at-risk.html

19. Heckathorn DD. Respondent-Driven Sampling: A New Approach to the Study of Hidden Populations. Soc Probl. 1997;44: 174–199. doi:10.2307/3096941

20. Elmes J, Skovdal M, Nhongo K, Ward H, Campbell C, Hallett TB, et al. A reconfiguration of the sex trade: How social and structural changes in eastern Zimbabwe left women involved in sex work and transactional sex more vulnerable. PLOS ONE. 2017;12: e0171916. doi:10.1371/journal.pone.0171916

21. UNAIDS. Sex work and HIV/AIDS, technical update. [Internet]. Geneva; 2002. Available: http://data.unaids.org/publications/IRC-pub02/jc705-sexwork-tu_en.pdf

22. Schur N, Mylne A, Mushati P, Takaruza A, Ward H, Nyamukapa C, et al. The effects of household wealth on HIV prevalence in Manicaland, Zimbabwe – a prospective household census and population-based open cohort study. J Int AIDS Soc. 2015;18. doi:10.7448/IAS.18.1.20063

23. Manzou R, Schumacher C, Gregson S. Temporal Dynamics of Religion as a Determinant of HIV Infection in East Zimbabwe: A Serial Cross-Sectional Analysis. PLOS ONE. 2014;9: e86060. doi:10.1371/journal.pone.0086060

For peer review only - http://bmjopen.bmj.com/site/about/guidelines.xhtml

24. Patel V, Simunyu E, Gwanzura F, Lewis G, Mann A. The Shona Symptom Questionnaire: the development of an indigenous measure of common mental disorders in Harare. Acta Psychiatr Scand. 1997;95: 469–475.

25. Tlhajoane M, Eaton JW, Takaruza A, Rhead R, Maswera R, Schur N, et al. Prevalence and Associations of Psychological Distress, HIV Infection and HIV Care Service Utilization in East Zimbabwe. AIDS Behav. 2017; 1–11. doi:10.1007/s10461-017-1705-x

26. Bourdillon M. The Shona peoples: An ethnography of the contemporary Shona, with special reference to their religion. 3rd ed. Gweru: Mambo Press; 1987.

27. Ross A, Van der Paal L, Lubega R, Mayanja BN, Shafer LA, Whitworth J. HIV-1 disease progression and fertility: the incidence of recognized pregnancy and pregnancy outcome in Uganda. AIDS Lond Engl. 2004;18: 799–804.

28. Mountain E, Mishra S, Vickerman P, Pickles M, Gilks C, Boily M-C. Antiretroviral Therapy Uptake, Attrition, Adherence and Outcomes among HIV-Infected Female Sex Workers: A Systematic Review and Meta-Analysis. PLOS ONE. 2014;9: e105645. doi:10.1371/journal.pone.0105645

29. Braunstein SL, Umulisa M-M, Veldhuijzen NJ, Kestelyn E, Ingabire CM, Nyinawabega J, et al. HIV diagnosis, linkage to HIV care, and HIV risk behaviors among newly diagnosed HIV-positive female sex workers in Kigali, Rwanda. J Acquir Immune Defic Syndr 1999. 2011;57: e70-76. doi:10.1097/QAI.0b013e3182170fd3

30. Bassett IV, Regan S, Chetty S, Giddy J, Uhler LM, Holst H, et al. Who starts antiretroviral therapy in Durban, South Africa?… not everyone who should. AIDS Lond Engl. 2010;24: S37–S44. doi:10.1097/01.aids.0000366081.91192.1c

31. Huet C, Ouedraogo A, Konaté I, Traore I, Rouet F, Kaboré A, et al. Long term virological, immunological and mortality outcomes in a cohort of HIV-infected female sex workers treated with highly active antiretroviral therapy in Africa. BMC Public Health. 2011;11: 700. doi:10.1186/1471-2458-11-700

32. Diabaté S, Zannou DM, Geraldo N, Chamberland A, Akakpo J, Ahouada C, et al. Antiretroviral Therapy among HIV-1 Infected Female Sex Workers in Benin: A Comparative Study with Patients from the General Population. World J AIDS. 2011;01: 94. doi:10.4236/wja.2011.13014

33. Paulin HN, Blevins M, Koethe JR, Hinton N, Vaz LM, Vergara AE, et al. HIV testing service awareness and service uptake among female heads of household in rural Mozambique: results from a province-wide survey. BMC Public Health. 2015;15. doi:10.1186/s12889-015-1388-z

34. NSWP. Stigma and Discrimination Experienced by Sex Workers Living with HIV [Internet]. 2015 Dec. Available: http://www.nswp.org/resource/stigma-and-discrimination-experienced-sex-workers-living-hiv

For peer review only - http://bmjopen.bmj.com/site/about/guidelines.xhtml

35. Skovdal M, Campbell C, Nyamukapa C, Gregson S. When masculinity interferes with women's treatment of HIV infection: a qualitative study about adherence to antiretroviral therapy in Zimbabwe. J Int AIDS Soc. 2011;14: 29. doi:10.1186/1758-2652-14-29

36. Zimbabwe Ministry of Health and Child Care. Zimbabwe Population-Based HIV Impact Assessment Survey. Harare, Zimbabwe; 2016.

For peer review only

Tables

Table 1: Socio-demographic characteristics associated with female involvement in sex work, and associations between sex work and intermediate determinants of HIV testing and treatment, Manicaland Zimbabwe, 2009-2011

Socio-demographic characteristic	FSW n	FSW %	NSW n	NSW %	N	AOR	95% CI
Age-group							
19-29	36	(3.7%)	950	(96.3%)	986	1	-
30-39	74	(9.4%)	710	(90.6%)	784	2.75	1.83-4.14
40-49	46	(8.%)	527	(92.%)	573	2.3	1.47-3.61
50-58	18	(4.7%)	368	(95.3%)	386	1.29	0.72-2.30
Marital status							
Never married	8	(10.5%)	68	(89.5%)	76	3.18	1.46-6.92
Married	87	(4.4%)	1912	(95.6%)	1999	1	-
Divorced or separated	41	(15.4%)	225	(84.6%)	266	3.76	2.52-5.62
Widowed	37	(9.6%)	350	(90.4%)	387	2.25	1.47-3.45
Church Denomination							
Christian	89	(6.%)	1385	(94.%)	1474	1	-
Spiritual	53	(5.7%)	882	(94.3%)	935	0.91	0.64-1.30
Other	22	(8.%)	253	(92.%)	275	1.35	0.83-2.21
None	10	(23.3%)	33	(76.7%)	43	4.47	2.10-9.52
Socio-economic status							
First (poorest) tercile	121	(6.9%)	1635	(93.1%)	1756	1	-
Second tercile	42	(6.8%)	572	(93.2%)	614	0.97	0.67-1.40
Third tercile	7	(2.6%)	263	(97.4%)	270	0.37	0.17-0.80
Residential area							
Town	64	(9.7%)	597	(90.3%)	661	1	-
Agricultural estate	40	(6.2%)	610	(93.8%)	650	0.59	0.39-0.89
Roadside settlement	45	(6.%)	702	(94.%)	747	0.59	0.40-0.89
Subsistence farming village	25	(3.7%)	646	(96.3%)	671	0.36	0.22-0.58
Education							
Primary or none	74	(7.3%)	944	(92.7%)	1018	1	-
Secondary or higher	100	(5.8%)	1611	(94.2%)	1711	0.78	0.54-1.11
Children alive							
None	44	(10.8%)	365	(89.2%)	409	1	-
1	45	(5.7%)	750	(94.3%)	795	0.54	0.34-0.83
2	43	(5.8%)	701	(94.2%)	744	0.46	0.29-0.72
3	20	(4.4%)	436	(95.6%)	456	0.27	0.15-0.47
4	22	(6.8%)	303	(93.2%)	325	0.39	0.22-0.67
Intermediate determinants							
**HIV testing**							
HIV Result							
Positive	91	(15.3%)	505	(84.7%)	596	4.00	2.90-5.50
Negative	82	(3.8%)	2048	(96.2%)	2130	1	-
Knowledge about HIV risks							
Good	158	(6.8%)	2167	(93.2%)	2325	1.63	0.96-2.76
Poor	16	(4.%)	388	(96.%)	404	1	-
Knowing persons living with or who PLHIV / died from HIV							
0	14	(2.7%)	506	(97.3%)	520	1	-
1 - 2	22	(4.6%)	455	(95.4%)	477	1.69	0.85-3.36
3 - 4	29	(6.1%)	449	(93.9%)	478	2.13	1.11-4.09
5 - 6	33	(6.7%)	458	(93.3%)	491	2.43	1.28-4.61
7	76	(10.%)	687	(90.%)	763	3.56	1.98-6.38
Risk perception for HIV infection							
Own high-risk behaviour	39	(48.8%)	41	(51.3%)	80	18.82	11.49-30.81
Partner(s)' high-risk behaviour	18	(9.8%)	166	(90.2%)	184	2.18	1.28-3.73
Other reasons	21	(13.2%)	138	(86.8%)	159	3.39	2.05-5.63
None	96	(4.2%)	2210	(95.8%)	2306	1.00	-
STD symptoms in last 12 months							
Yes	29	(11.9%)	215	(88.1%)	244	2.05	1.34-3.13
No	145	(5.8%)	2340	(94.2%)	2485	1	-
Sickness in last 12 months							
HIV-related illness	23	(20.%)	92	(80.%)	115	4.09	2.41-6.93
Other illness	81	(6.7%)	1125	(93.3%)	1206	1.37	0.98-1.91
None	69	(4.9%)	1335	(95.1%)	1404	1	-
Psychological distress							
Yes	43	(12.6%)	298	(87.4%)	341	1.31	1.60-3.34

For peer review only

For peer review only - http://bmjopen.bmj.com/site/about/guidelines.xhtml

No	131	(5.5%)	2257	(94.5%)	2388	1	-
Pregnancies in last 3 years							
One or more	49	(4.4%)	1077	(95.6%)	1126	0.59	0.40-0.87
None	125	(7.8%)	1478	(92.2%)	1603	2	-
Stigma and discrimination (affecting testing)							
Yes	2	(7.1%)	26	(92.9%)	28	0.05	0.25-4.49
No	172	(6.4%)	2529	(93.6%)	2701	1	-
Travel time to HIV testing facility							
<30 mins	61	(13.5%)	390	(86.5%)	451	1	-
30-59 mins	39	(6.3%)	585	(93.8%)	624	0.42	0.27-0.64
60-89 mins	23	(3.8%)	587	(96.2%)	610	0.24	0.15-0.40
90 mins	48	(5.4%)	849	(94.6%)	897	0.35	0.23-0.52
Uncertain	3	(2.%)	144	(98.%)	147	-	-
**Antiretroviral treatment**							
Knowledge of ART							
Yes	126	(8.6%)	1341	(91.4%)	1467	1.35	1.67-3.31
No	48	(3.8%)	1203	(96.2%)	1251	1	-
Stigma and discrimination (in the community)							
Yes	43	(8.5%)	462	(91.5%)	505	0.46	1.01-2.08
No	131	(5.9%)	2090	(94.1%)	2221	1	-
Peer influence							
Relative(s) on ART	41	(8.%)	469	(92.%)	510	2.14	1.41-3.24
Friend(s) on ART	55	(14.2%)	333	(85.8%)	388	3.98	2.69-5.89
None	58	(3.7%)	1496	(96.3%)	1554	1	-
Travel time to ART service *							
<30 mins	34	(16.5%)	172	(83.5%)	206	1	-
30-59 mins	17	(6.1%)	261	(93.9%)	278	0.31	0.17-0.57
60-89 mins	17	(7.1%)	224	(92.9%)	241	0.38	0.20-0.70
90 mins	33	(8.8%)	341	(91.2%)	374	0.46	0.28-0.78
Uncertain	73	(4.5%)	1557	(95.5%)	1630	-	-

AOR- age-adjusted odds ratios; 95% CI- 95% confidence intervals
* Includes women not aware of HIV testing and ART services to prevent exclusion of these participants from the multi-variable analysis. Odds ratios were not interpreted for this group as they are not comparable with the reference category

For peer review only

Table 2: Factors contributing to differences in uptake of HIV testing between FSW and NSW, Manicaland, Zimbabwe, 2009-2011 (N=2729)

			Bivariate Analysis			Socio-demographic		Socio-demographic + Sexwork		Intermediate Determinants		Intermediate Determinants + Sexwork		Full Model	
		n	%	AOR	95% CI	AOR	95% CI	AOR	95% CI	AOR	95% CI	AOR	95% CI	AOR	95% CI
**Female Sex Work**															
Sex Work	NSW	1925	(75.3%)	1	-	-	-	1	-	-	-	1	-	1	-
	FSW	142	(81.6%)	1.5	1.00-2.24	-	-	1.75	1.14-2.69	-	-	0.99	0.63-1.57	1.11	0.69-1.81
**Socio-demographic**															
Age-group															
	19-29	822	(83.4%)	1	-	1	-	1	-	-	-	-	-	1	-
	30-39	622	(79.3%)	0.75	0.59-0.95	0.69	0.53-0.90	0.67	0.51-0.87	-	-	-	-	0.65	0.48-0.89
	40-49	402	(70.2%)	0.46	0.36-0.59	0.46	0.34-0.61	0.45	0.33-0.60	-	-	-	-	0.54	0.37-0.78
	50-58	221	(57.3%)	0.27	0.20-0.35	0.29	0.21-0.41	0.29	0.21-0.41	-	-	-	-	0.39	0.26-0.59
Marital status															
	Never Married	283	(73.1%)	0.28	0.17-0.45	0.31	0.18-0.50	0.3	0.18-0.49	-	-	-	-	0.43	0.24-0.78
	Married	42	(55.3%)	1	-	1	-	1	-	-	-	-	-	1	-
	Divorced or separated	1551	(77.6%)	0.78	0.58-1.05	0.8	0.59-1.09	0.77	0.56-1.05	-	-	-	-	0.83	0.58-1.17
	Widowed	191	(71.8%)	1.3	0.99-1.70	1.4	1.06-1.85	1.38	1.04-1.82	-	-	-	-	1.26	0.92-1.72
Church Denomination															
	Christian	1131	(76.7%)	1	-	1	-	1	-	-	-	-	-	1	-
	Spiritual	701	(75.%)	0.84	0.69-1.03	0.89	0.72-1.09	0.89	0.73-1.10	-	-	-	-	0.95	0.75-1.20
	Other	203	(73.8%)	0.78	0.58-1.06	0.87	0.63-1.20	0.87	0.63-1.20	-	-	-	-	0.94	0.65-1.34
	None	30	(69.8%)	0.56	0.28-1.09	0.56	0.28-1.13	0.52	0.26-1.06	-	-	-	-	0.43	0.20-0.93
Socio-economic status															
	First (poorest) tercile	1308	(74.5%)	1	-	1	-	1	-	-	-	-	-	1	-
	Second tercile	472	(76.9%)	1.13	0.90-1.41	1.04	0.83-1.30	1.04	0.83-1.31	-	-	-	-	0.94	0.73-1.22
	Third tercile	219	(81.1%)	1.35	0.97-1.87	1.06	0.74-1.53	1.1	0.77-1.59	-	-	-	-	0.9	0.60-1.34
Residential area															
	Town	542	(82.%)	1	-	1	-	1	-	-	-	-	-	1	-
	Agricultural estate	460	(70.8%)	0.57	0.44-0.74	0.59	0.44-0.79	0.61	0.46-0.82	-	-	-	-	0.63	0.45-0.88
	Roadside settlement	569	(76.2%)	0.81	0.62-1.06	0.79	0.59-1.05	0.81	0.60-1.08	-	-	-	-	0.94	0.68-1.30
	Subsistence farming village	496	(73.9%)	0.71	0.54-0.92	0.7	0.52-0.95	0.73	0.55-0.99	-	-	-	-	0.93	0.67-1.31
Education															
	Primary or less	675	(66.3%)	1	-	1	-	1	-	-	-	-	-	1	-
	Secondary or higher	1392	(81.4%)	1.47	1.18-1.83	1.45	1.15-1.82	1.46	1.16-1.83	-	-	-	-	1.06	0.81-1.37
Children alive															
	None	257	(62.8%)	1	-	1	-	1	-	-	-	-	-	1	-
	1	632	(79.5%)	1.9	1.45-2.50	1.63	1.23-2.16	1.67	1.26-2.22	-	-	-	-	1.52	1.10-2.09
	2	586	(78.8%)	1.79	1.35-2.36	1.53	1.14-2.05	1.57	1.17-2.11	-	-	-	-	1.48	1.06-2.06
	3	343	(75.2%)	1.6	1.18-2.18	1.43	1.04-1.98	1.5	1.08-2.07	-	-	-	-	1.45	1.01-2.08
	4	249	(76.6%)	1.88	1.33-2.65	1.73	1.21-2.48	1.79	1.24-2.57	-	-	-	-	1.72	1.14-2.59
**Intermediate Determinants**															
HIV Result															
	Positive	454	(76.2%)	1.09	0.88-1.36	-	-	-	-	-	-	-	-	-	-

For peer review only - http://bmjopen.bmj.com/site/about/guidelines.xhtml

Negative	1611	(75.6%)	1	-	-	-	-	-	-	-	-	-	-	-
Knowledge about HIV risks														
Good	1787	(76.9%)	1.5	1.18-1.90	-	-	-	-	1.42	1.08-1.86	1.42	1.08-1.86	1.35	1.02-1.80
Poor	280	(69.3%)	1	-	-	-	-	-	1	-	1	-	1	-
Risk perception for HIV infection														
Own high-risk behaviour	68	(85.%)	2.36	1.26-4.43	-	-	-	-	1.21	0.62-2.36	1.21	0.60-2.43	1.3	0.63-2.70
Partner(s)' high-risk behaviour	172	(93.5%)	6.21	3.41-11.29	-	-	-	-	4.53	2.30-8.94	4.53	2.30-8.94	4.61	2.29-9.29
Other reasons	133	(83.7%)	2.12	1.37-3.29	-	-	-	-	1.47	0.92-2.35	1.47	0.92-2.35	1.41	0.87-2.29
None	1694	(73.5%)	1	-	-	-	-	-	1	-	1	-	1	-
Knowing persons living with or who PLHIV / died from HIV														
0	344	(66.2%)	1	-	-	-	-	-	1	-	1	-	1	-
1 - 2	350	(73.4%)	1.51	1.14-2.00	-	-	-	-	1.14	0.83-1.57	1.14	0.83-1.57	1.12	0.80-1.57
3 - 4	392	(82.%)	2.56	1.89-3.47	-	-	-	-	2.09	1.48-2.95	2.09	1.48-2.95	2.04	1.43-2.91
5 - 6	389	(79.2%)	2.26	1.69-3.04	-	-	-	-	1.53	1.11-2.12	1.53	1.11-2.12	1.47	1.05-2.06
7	592	(77.6%)	2.07	1.60-2.69	-	-	-	-	1.35	1.01-1.82	1.35	1.01-1.82	1.27	0.93-1.72
STD symptoms in last 12 months														
Yes	195	(79.9%)	1.5	1.00-2.24	-	-	-	-	0.83	0.57-1.20	0.83	0.57-1.20	0.83	0.56-1.22
No	1872	(75.3%)	1	-	-	-	-	-	1	-	1	-	1	-
Sickness in last 12 months														
HIV-related illness	109	(94.8%)	8.08	3.50-18.67	-	-	-	-	3.78	1.55-9.18	3.78	1.55-9.18	4.35	1.76-10.71
Other illness	904	(75.%)	1.04	0.87-1.25	-	-	-	-	0.97	0.80-1.19	0.97	0.80-1.19	0.97	0.78-1.20
None	1052	(74.9%)	1	-	-	-	-	-	1	-	1	-	1	-
Psychological distress														
Yes	259	(76.%)	1.12	0.85-1.47	-	-	-	-	-	-	-	-	-	-
No	1808	(75.7%)	1	-	-	-	-	-	-	-	-	-	-	-
Pregnancies in last 3 years														
One or more	974	(86.5%)	2.32	1.82-2.96	-	-	-	-	2.51	1.91-3.28	2.5	1.91-3.28	2.42	1.82-3.22
None	1093	(68.2%)	1	-	-	-	-	-	1	-	1.00	-	1	-
Travel time to HIV testing facility*														
<30 mins	385	(85.4%)	1	-	-	-	-	-	1	-	1	-	1	-
30 - 59 mins	508	(81.4%)	0.78	0.56-1.09	-	-	-	-	0.73	0.51-1.03	0.73	0.51-1.03	0.73	0.51-1.06
60 - 89 mins	470	(77.1%)	0.63	0.45-0.87	-	-	-	-	0.58	0.41-0.82	0.58	0.41-0.82	0.5	0.35-0.73
90 mins	690	(76.9%)	0.63	0.46-0.86	-	-	-	-	0.58	0.42-0.80	0.58	0.42-0.80	0.47	0.33-0.67
Uncertain	14	(9.5%)	-	-	-	-	-	-	-	-	-	-	-	-
Knowledge of ART														
Yes	1224	(83.4%)	2.38	1.98-2.86	-	-	-	-	1.51	1.23-1.87	1.51	1.23-1.87	1.48	1.19-1.85
No	833	(66.6%)	1	-	-	-	-	-	1	-	1	-	1	-
Stigma and discrimination (affecting testing)														
Yes	19	(67.9%)	0.76	0.34-1.73	-	-	-	-	-	-	-	-	-	-
No	2048	(75.8%)	1	-	-	-	-	-	-	-	-	-	-	-
Stigma and discrimination (in the community)														
Yes	395	(78.2%)	1.18	0.93-1.50	-	-	-	-	-	-	-	-	-	-
No	1669	(75.2%)	1	-	-	-	-	-	-	-	-	-	-	-

AOR- age-adjusted odds ratios; 95% CI- 95% confidence intervals

* Includes women not aware of HIV testing and ART services to prevent exclusion of these participants from the multi-variable analysis. Odds ratios were not interpreted for this group as they are not comparable with the reference category

For peer review only

Table 3: Factors contributing to differences in uptake of antiretroviral treatment between FSW and NSW, Manicaland, Zimbabwe, 2009-2011

		Bivariate Analysis				Socio-demographic		Socio-demographic Sexwork		Intermediate Determinants		Intermediate Determinants Sexwork		Full Model	
		n	%	AOR	95% CI	AOR	95% CI	AOR	95% CI	AOR	95% CI	AOR	95% CI	AOR	95% CI
**Female Sex Work**															
**Sex Work**	NSW	138	(64.2%)	1	-	-	-	1	-	-	-	1	-	1	-
	FSW	45	(84.9%)	2.64	1.16-6.00	-	-	2.28	0.97-5.39	-	-	3.46	0.91-13.16	3.51	0.79-15.47
**Socio-demographic**															
**Age**															
	Age (continuous)	-	-	1.53	1.14-2.06	1.62	1.19-2.22	1.57	1.14-2.15	-	-	-	-	1.63	1.03-2.57
	Age2	-	-	1	0.99-1.00	0.99	0.99-1.00	1	0.99-1.00	-	-	-	-	1	0.99-1.00
**Marital status**															
	Never Married	2	(50.%)	0.33	0.04-2.57	-	-	-	-	-	-	-	-	-	-
	Married	72	(59.%)	1	-	-	-	-	-	-	-	-	-	-	-
	Divorced/Separated	29	(74.4%)	1.56	0.66-3.65	-	-	-	-	-	-	-	-	-	-
	Widowed	80	(77.7%)	1.41	0.73-2.74	-	-	-	-	-	-	-	-	-	-
**Church Denomination**															
	Christian churches	100	(73.5%)	1	-	-	-	-	-	-	-	-	-	-	-
	Spiritual churches	59	(59.6%)	0.62	0.35-1.12	-	-	-	-	-	-	-	-	-	-
	Other religion	19	(73.1%)	1.29	0.46-3.58	-	-	-	-	-	-	-	-	-	-
	No religion	5	(71.4%)	2	0.30-13.48	-	-	-	-	-	-	-	-	-	-
**Socio-economic status**															
	1 (poorest)	113	(68.1%)	1	-	-	-	-	-	-	-	-	-	-	-
	2	47	(69.1%)	1.21	0.63-2.34	-	-	-	-	-	-	-	-	-	-
	3	20	(74.1%)	2.3	0.82-6.44	-	-	-	-	-	-	-	-	-	-
**Residential area**															
	Town	62	(70.5%)	1	-	1	-	1	-	-	-	-	-	1	-
	Agricultural estate	54	(76.1%)	1.1	0.52-2.35	1.27	0.58-2.76	1.37	0.62-3.01	-	-	-	-	2.53	0.77-8.29
	Roadside settlement	35	(58.3%)	0.48	0.23-1.01	0.54	0.26-1.15	0.6	0.28-1.28	-	-	-	-	0.87	0.29-2.61
	Subsistence farming village	32	(65.3%)	0.82	0.37-1.82	0.96	0.42-2.16	1.01	0.44-2.30	-	-	-	-	1.78	0.53-5.95
**Education**															
	Primary or less	86	(71.7%)	1	-	-	-	-	-	-	-	-	-	-	-
	Secondary or higher	97	(65.5%)	1.09	0.58-2.07	-	-	-	-	-	-	-	-	-	-
**Children alive**															
	0	50	(84.8%)	1	-	1.00	-	1	-	-	-	-	-	1	-
	1	52	(66.7%)	0.44	0.18-1.08	0.44	0.18-1.10	0.49	0.19-1.24	-	-	-	-	0.2	0.04-1.12
	2	40	(63.5%)	0.37	0.15-0.95	0.39	0.15-1.02	0.46	0.18-1.22	-	-	-	-	0.13	0.02-0.76
	3	24	(58.5%)	0.27	0.10-0.75	0.27	0.10-0.74	0.31	0.11-0.87	-	-	-	-	0.13	0.02-0.80
	4	17	(63.%)	0.24	0.08-0.72	0.24	0.08-0.75	0.25	0.08-0.78	-	-	-	-	0.05	0.01-0.36
**Intermediate Determinants**															
**Knowledge about HIV risks**															
	Good	162	(69.8%)	1.18	0.53-2.63	-	-	-	-	-	-	-	-	-	-

For peer review only

For peer review only - http://bmjopen.bmj.com/site/about/guidelines.xhtml

	Poor	21	(58.3%)	1	-	-	-	-	-	-	-	-	-	-	-
**Risk perception for HIV infection**															
	Own high-risk behaviour	37	(72.6%)	1.1	0.38-3.16	-	-	-	-	-	-	-	-	-	-
	Partner(s)' high-risk behaviour	92	(69.2%)	1.06	0.42-2.68	-	-	-	-	-	-	-	-	-	-
	Other reasons	36	(63.2%)	0.81	0.29-2.26	-	-	-	-	-	-	-	-	-	-
	None	18	(66.7%)	1	-	-	-	-	-	-	-	-	-	-	-
**Peer influence**															
	Relative(s) on ART	72	(75.8%)	9.12	4.25-19.58	-	-	-	-	1.95	0.70-5.42	1.83	0.66-5.06	1.79	0.61-5.20
	Friend(s) on ART	91	(87.5%)	16.38	7.22-37.20	-	-	-	-	3.04	1.03-8.93	2.52	0.85-7.46	2.19	0.69-6.97
	None	20	(29.%)	1	-	-	-	-	-	1	-	1	-	1	-
**Sickness in last 12 months**															
	HIV-related illness	69	(72.6%)	1.09	0.57-2.09	-	-	-	-	-	-	-	-	-	-
	Other illness	42	(61.8%)	0.69	0.35-1.35	-	-	-	-	-	-	-	-	-	-
	None	72	(68.6%)	1	-	-	-	-	-	-	-	-	-	-	-
**STD symptoms in last 12 months**															
	Yes	51	(68.%)	1.03	0.56-1.89	-	-	-	-	-	-	-	-	-	-
	No	132	(68.4%)	1	-	-	-	-	-	-	-	-	-	-	-
**Psychological distress**															
	Yes	36	(58.1%)	0.46	0.24-0.86	-	-	-	-	0.43	0.19-0.99	0.41	0.18-0.96	0.48	0.20-1.18
	No	147	(71.4%)	1	-	-	-	-	-	1	-	1	-	1	-
**Pregnancies in last 3 years**															
	One or more	28	(50.9%)	0.86	0.41-1.80	-	-	-	-	-	-	-	-	-	-
	None	155	(72.8%)	1	-	-	-	-	-	-	-	-	-	-	-
**Travel time to ART service***															
	<30 mins	43	(87.8%)	1	-	-	-	-	-	1	-	1	-	1	-
	30 - 59 mins	42	(73.7%)	0.27	0.08-0.86	-	-	-	-	0.27	0.08-0.89	0.34	0.10-1.15	0.41	0.11-1.58
	60 - 89 mins	34	(81.%)	0.32	0.09-1.18	-	-	-	-	0.37	0.10-1.40	0.42	0.11-1.62	0.34	0.07-1.67
	90 mins	63	(80.8%)	0.37	0.12-1.16	-	-	-	-	0.46	0.14-1.46	0.51	0.15-1.65	0.65	0.17-2.49
	Uncertain	1	(2.4%)	-	-	-	-	-	-	-	-	-	-	-	-
**Stigma and discrimination (in the community)**															
	Yes	38	(71.7%)	1.3	0.64-2.64	-	-	-	-	-	-	-	-	-	-
	No	145	(67.8%)	1	-	-	-	-	-	-	-	-	-	-	-

AOR- age-adjusted odds ratios; 95% CI- 95% confidence intervals

* Includes women not aware of HIV testing and ART services to prevent exclusion of these participants from the multi-variable analysis. Odds ratios were not interpreted for this group as they are not comparable with the reference category

For peer review only

For peer review only - http://bmjopen.bmj.com/site/about/guidelines.xhtml

Fig. 1: Theoretical framework illustrating how engagement in sex work (or not) may influence use of HIV testing and treatment services (dashed line A). This framework hypothesises that individuals' uptake of services may be influenced by various socio-demographic characteristics (dashed line B), and that these factors may be mediated by involvement in sex work which, in turn, alters uptake of services. Involvement in sex work is not considered to alter uptake of services *per se*; rather, engagement in sex work is associated with different social, structural and psychosocial experiences compared to non-sex workers which, in turn, may drive differential uptake of services by sex work status.

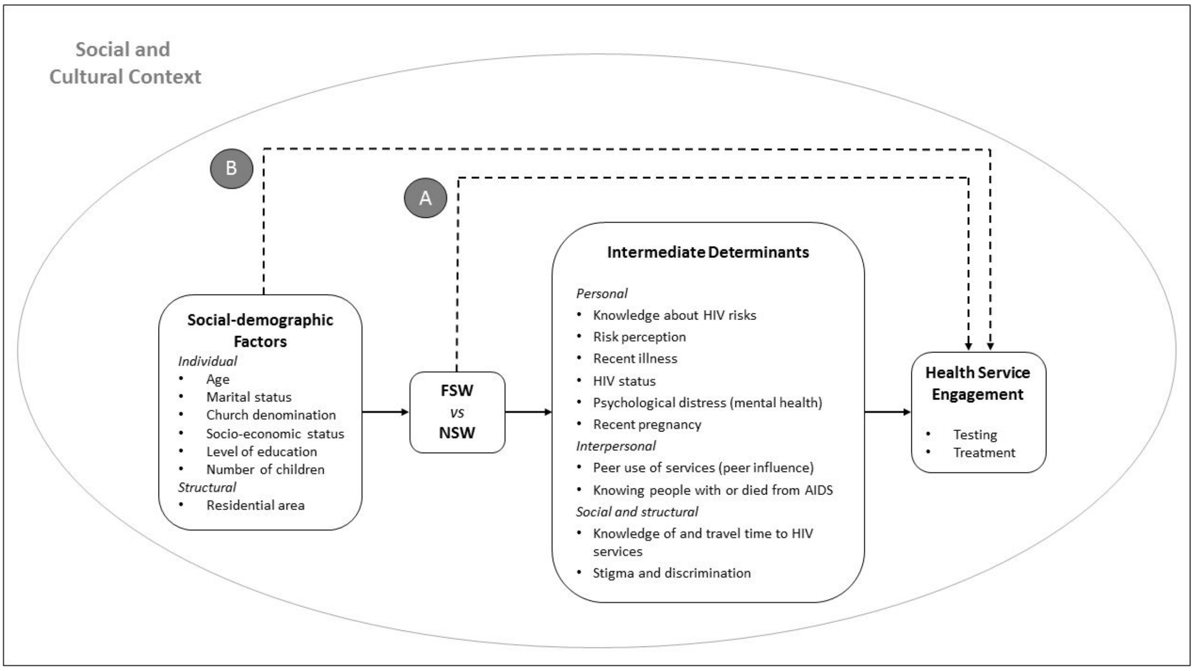

Fig 2a: Comparison of HIV treatment cascades for female sex workers and non-sex workers in Manicaland, Zimbabwe, 2009-2011.

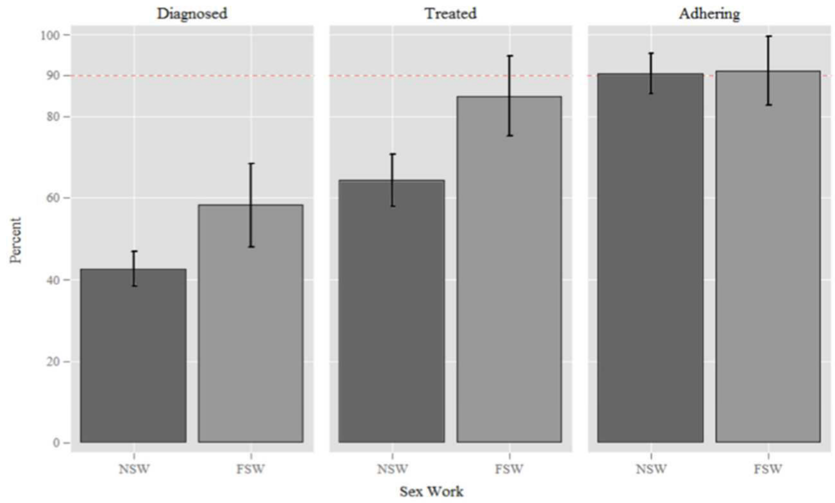

Fig 2b: Comparison of cumulative HIV treatment cascades for female sex workers and non-sex workers in Manicaland, Zimbabwe, 2009-2011.

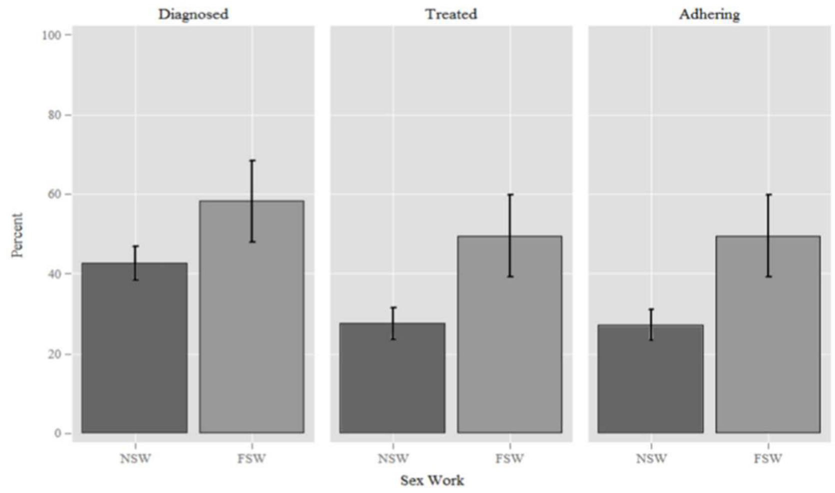

Figure 2a & 2b: Comparisons of HIV treatment cascades. Figures illustrating the proportion of FSW and NSW who achieve optimal outcomes at each stage of the cascade. Fig 2a shows the proportions of HIV-positive women who have been diagnosed, the proportions of treated amongst those who have been diagnosed, and the proportions adhering to their medication amongst those who have been treated. A 90% reference line is included to illustrate UNAIDS targets. For Fig 2b, the denominator is all HIV-positive women at each stage of the cascade.

Supplementary Material

A. Theoretical framework for factors mediating uptake of HIV services among female sex workers vs non-sex workers

Several theoretical models of the mechanisms for behaviour change have been successfully applied to reduce risky sexual behaviour by individuals [1]. However, recognition of the limitations of individual-level approaches to HIV prevention (such as to what extent condom use is solely related to self-efficacy without consideration of gendered power dynamics) has led to a growth in structural models for HIV risk [2]. Multilevel theoretical models draw on the strengths of both individual-level focused models and structural models, but in delineating the links between these levels have great potential power for theory-driven approaches to combination prevention [3]. For example, the Network-Individual-Resource Model (NIRM) for HIV Prevention posits that membership of distinct social networks can attenuate or enhance individual-level factors driving HIV risk [4]. For example, intense stigma and discrimination frequently marginalises female sex workers (FSW) from wider society such that FSW occupy distinct social networks from non-sex workers (NSW). Therefore, various factors relating these distinct social networks may mean that FSWs' use of healthcare services may be dissimilar to that of NSW. These factors comprise individual-level preferences and behaviours as well as relations with peers, relatives and the community and the legal and socio-cultural context. Drawing on a rich literature of multi-level theoretical approaches to behaviour change and HIV transmission prevention [3–5], we describe a new framework (Figure S1) to explain how intermediate factors at different levels may be

For peer review only - http://bmjopen.bmj.com/site/about/guidelines.xhtml

associated with HIV service uptake in testable relations in a Zimbabwean context, a subset of which are explored in this paper (Figure 1).

Supplementary Figure 1: Generalised theoretical framework for intermediate factors mediating differential uptake of HIV services by female sex workers (FSW) compared to non-sex workers (NSW).

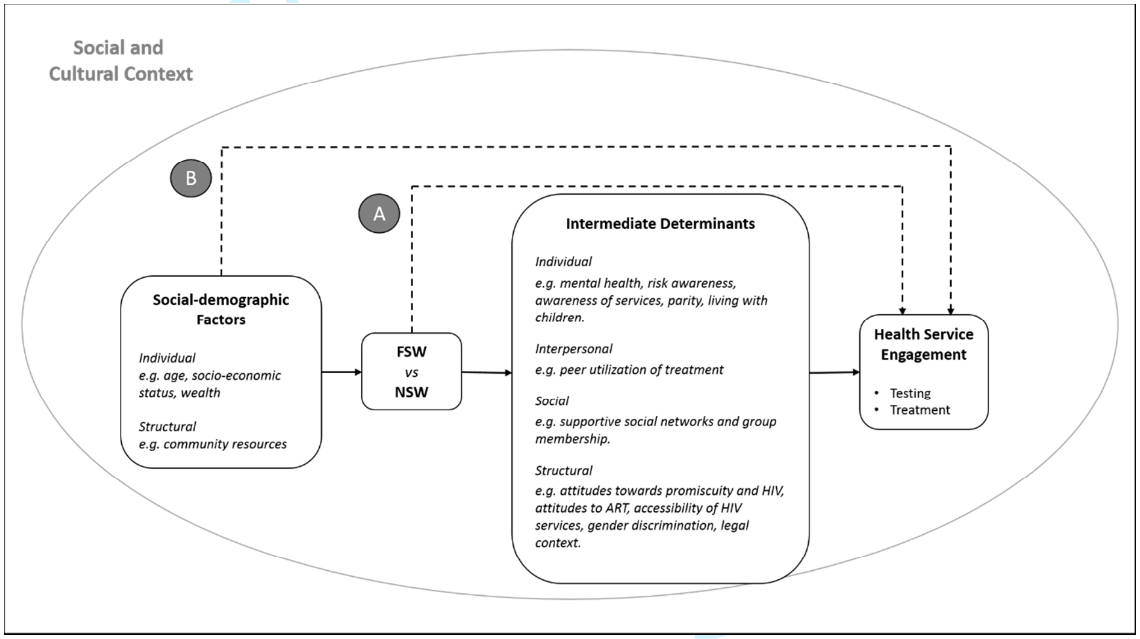

For peer review only - http://bmjopen.bmj.com/site/about/guidelines.xhtml

Influence of structural factors on uptake of health services

Structural factors include social, cultural, economic, legal and political contexts which shape and frame behaviours, actions and norms of communities and agents [2]. In meta-analyses evaluating barriers to retention and linkage to care, distance from testing facilities and costs of transport have been identified as the most important obstacles [6], yet to what extent this is true in FSW as well as NSW is unclear. Since FSW tend to most commonly live in more urban areas than NSW where facilities are most concentrated [6], we might expect distances and costs of transport to be different between FSW and NSW. However, travel also incurs an opportunity cost for FSW who experience loss of earnings during the time taken to travel [7] so this may attenuate their uptake relative to NSW. In addition, FSW often migrate both internally and across national borders [8,9], often away from families even including children. Mobility and migration affect uptake of services in complex ways that are dependent on a variety of contextual factors (e.g. relative availability of services in source and sink destinations, whether migration is internal, circular or international). Migration from high to low prevalence settings is associated with lower HIV risk [10] but migrants are more likely to be unaware of local services which can reduce access. In addition, circular migration can interrupt treatment or cause delays in treatment [11].

Fear of social rejection and discrimination from positive HIV diagnosis often deters individuals from seeking testing [7,12,13]. For FSW, this fear may be more intense because of higher rates of HIV among FSW than NSW (see individual-factors below) and because they already

experience intense stigma and discrimination as a result of selling sex. Laws criminalising sex work mean that sex workers are often subject to arrest and violence perpetrated by police [2,14,15]. Such laws often intersect with gendered attitudes towards acceptable behaviour for

For peer review only - http://bmjopen.bmj.com/site/about/guidelines.xhtml

women, often compounding long-lived taboos around female promiscuity [16,17]. Such stigma frequently results in harassment [16] and can cause unnecessary delays to treatment from healthcare workers [18] or deter FSW from accessing care altogether [19].

Influence of social factors on uptake of health services

Evidence for social factors influencing uptake is based on trials of various interventions to encourage treatment initiation and adherence. In the wider community, mobilisation, group membership and empowerment (either informal or formal) have been successful in encouraging HIV testing and treatment through enhancing social capital (networks of inter-group relationships that are socially enhancing) and self-efficacy [20,21]. Similar approaches have been targeted towards mobilising sex worker communities (e.g. by uniting sex workers in a common cause for health improvement, creating spaces for debate of new health information and tackling powerful actors that actively disenfranchise sex workers through violence, stigma or discrimination [22] have demonstrated substantial effectiveness in reducing HIV infection and other STIs and increasing condom use [23]. An important component of community mobilisation is the development and strengthening of social capital and facilitating "transformative social spaces". One approach to this is encouraging participation in community groups. Such groups can have powerful positive impacts on risk behaviours and healthcare seeking, by providing a critical dialogue of harmful social norms, providing emotional and material support and by forming positive action plans and solidarity to mobilise them [21]. Conversely, they can also entrench negative norms and facilitate dissemination of false information. It is unclear how community membership may have differential impact on FSW and NSW in enhancing/attenuating service uptake.

For peer review only - http://bmjopen.bmj.com/site/about/guidelines.xhtml

Influence of individual and interpersonal level factors on uptake of health services

A complex interplay of biological and behavioural factors drive differences in HIV risk in FSW compared to NSW which in turn will influence their respective need for and exposure to HIV services. Unsurprisingly, awareness and knowledge of HIV services have been identified as a critical component to encouraging service uptake. Batona et al found FSW who had previously undergone HIV counselling and testing (HTC) were more likely to become engaged with services a second time and displayed less resistance to testing and initiation in the treatment cascade [24]. A synergistic and reciprocal relationship exists between STIs (such as HSV-2 and bacterial vaginosis) and HIV such that acquisition of one can facilitate acquisition and transmission of the other [25–27]. Unprotected sex with multiple sexual partners puts FSWs at greater risk than NSW of symptomatic STIs and HIV. Consequently, FSW may be more likely to access services than NSW to resolve these health concerns, not least because ill-health may cause loss of earnings. Relatedly, greater perceived risk among FSWs may drive higher rates of health service uptake [1,8].

For many women worldwide, initial exposure to HIV testing is via antenatal care services (ANC). We might expect lower exposure to HIV testing through ANC for FSW for a couple of reasons. First, since FSW have higher prevalence of HIV than NSW and HIV reduces fertility [12] , we might expect incidence of pregnancy among FSW to be lower. Second, pregnancy represents an opportunity cost for FSW (loss of earnings) and so they may be more likely to take steps to avoid it (e.g. hormonal contraception).

Differences in wealth of FSW compared to NSW may mean they have different capacities to pay for healthcare-related costs [28]; conversely by not living with children (either because they have no children or have travelled to work), FSW may have lower childcare related

For peer review only - http://bmjopen.bmj.com/site/about/guidelines.xhtml

expenditures than NSW which may mean greater disposable income for healthcare-related expenditures [29].

High rates of mental health disorders in FSW occurs because of discrimination and social rejection, abusive acts of violence and economic pressures to support dependents [5]. Data from Zimbabwe suggest FSW have higher levels of mental ill-health than NSW and that mental ill-health is linked to poorer adherence to ART [30]. In addition to the fear of HIV positive diagnosis, disclosure as HIV positive connotes additional negative consequences for FSW, it being undesirable for potential clients and potentially resulting in a loss of earnings.

In frameworks for HIV risk, interpersonal factors include frequency and type of sexual relationships and the negotiation of condom use therein [2,5]. Intimate male partners can effectively control their female spouse's access to HIV treatment, causing substantial treatment delays [6,13] intimate partner violence has been linked to lower ART use and viral load suppression [31] and for FSW having an intimate partnership can present a significant obstacle to achieving viral suppression [32]. If NSW are more likely to have an intimate male partners than FSW, we might therefore expect uptake among NSW to be more affected by the influence of partners.

The impact of interpersonal factors on health-service uptake need not relate solely to sexual relationships but may also be driven through social relationships. In HIV prevention, use of peers has had important beneficial impact in enhancing knowledge of HIV risks, encouraging condom use and reducing HIV/STI infections [33,34]. Use of peers to encourage uptake of HIV care is less well studied. In India, a requirement to take a "buddy" or family member before treatment was issued prevented FSW and MSM from accessing services [7] and peer-

For peer review only - http://bmjopen.bmj.com/site/about/guidelines.xhtml

led interventions may be limited if the social environment is not health-enabling [35]. Nevertheless, peers have been used with some success in preventing mother-to-child transmission of HIV [36] and near-peers (health workers with shared cultural background with clients) have been used in the US to significantly increase viral load suppression by helping patients navigate non-integrated HIV care systems [37]. We hypothesis peer use of HIV care as a potential factor to encourage service access.

B. Shona Symptom Questionnaire

Mental health was assessed using the Shona Symptom Questionnaire (SSQ), a 14-item questionnaire of 'yes or 'no' questions, developed and validated in Zimbabwe in 1997 with the aid of mental healthcare providers [38]. The SSQ quantifies psychological distress as a function of somatic and psychological experiences over the week prior to interview. Using validated cut-points indicating levels of psychological distress [38], a dichotomous variable (0/1) was created with individuals with an SSQ score ≥7 (coded 1) as currently experiencing psychological distress [30].

For peer review only - http://bmjopen.bmj.com/site/about/guidelines.xhtml

C. HIV testing amongst HIV+ women

Table S1: Factors contributing to the difference in uptake of HIV testing between HIV-positive FSW and NSW, Manicaland Zimbabwe, 2009-2011

				Bivariate		Socio-demographic		Socio-demographic Sexwork		Intermediate Determinants		Intermediate Determinants Sexwork		Full Model	
				AOR	95% CI	AOR	95% CI	AOR	95% CI	AOR	95% CI	AOR	95% CI	AOR	95% CI
**Female Sex Work**															
Sex Work	NSW	379	(75.1%)	1	-	-	-	1	-	-	-	1	-	1	-
	FSW	75	(82.4%)	1.51	0.85-2.70	-	-	1.83	1.00-3.37	-	-	1.02	0.51,2.05	1.14	0.56,2.35
**Socio-demographic**															
Age-group															
	19-29	96	(75.%)	1	-	-	-	-	-	-	-	-	-	-	-
	30-39	178	(76.1%)	1.06	0.64-1.75	-	-	-	-	-	-	-	-	-	-
	40-49	126	(79.8%)	1.31	0.75-2.29	-	-	-	-	-	-	-	-	-	-
	50-58	54	(71.1%)	0.82	0.43-1.55	-	-	-	-	-	-	-	-	-	-
Marital status															
	Never married	139	(80.8%)	0.52	0.18-1.52	-	-	-	-	-	-	-	-	-	-
	Married	9	(60.%)	1	-	-	-	-	-	-	-	-	-	-	-
	Divorced or separated	237	(75.2%)	0.90	0.53-1.54	-	-	-	-	-	-	-	-	-	-
	Widowed	69	(73.4%)	1.45	0.89-2.38	-	-	-	-	-	-	-	-	-	-
Religion															
	Christian	238	(78.%)	1	-	1	-	1	-	-	-	-	-	1	-
	Spiritual	156	(76.9%)	0.93	0.60-1.42	1.00	0.65-1.55	1.02	0.66-1.58	-	-	-	-	1.09	0.64,1.85
	Other	48	(70.6%)	0.69	0.38-1.24	0.77	0.42-1.42	0.76	0.41-1.39	-	-	-	-	0.79	0.38,1.63
	None	12	(60.%)	0.42	0.16-1.08	0.50	0.19-1.33	0.41	0.15-1.12	-	-	-	-	0.33+	0.10,1.07
Socio-economic status															

For peer review only

For peer review only - http://bmjopen.bmj.com/site/about/guidelines.xhtml

First (poorest) tercile	284	(74.9%)	1	-	-	-	-	-	-	-	-	-	-	-
Second tercile	114	(79.7%)	1.31	0.82-2.10	-	-	-	-	-	-	-	-	-	-
Third tercile	48	(76.2%)	1.08	0.58-2.02	-	-	-	-	-	-	-	-	-	-
Residential area														
Town	143	(73.7%)	1	-	1	-	1	-	-	-	-	-	1	-
Agricultural estate	125	(74.4%)	1.02	0.64-1.64	1.02	0.63-1.65	1.06	0.65-1.73	-	-	-	-	1.53	0.84,2.79
Roadside settlement	79	(76.7%)	1.61	0.93-2.79	1.46	0.83-2.54	1.52	0.87-2.67	-	-	-	-	2.25*	1.14,4.42
Subsistence farming village	107	(81.7%)	1.20	0.69-2.10	1.09	0.61-1.93	1.13	0.64-2.01	-	-	-	-	1.47	0.70,3.10
Education														
Primary or none	178	(71.2%)	1	-	1	-	1	-	-	-	-	-	1	-
Secondary or higher	276	(79.8%)	1.75	1.14-2.71	1.63	1.04-2.53	1.66	1.06-2.58	-	-	-	-	1.47	0.85,2.55
Children alive														
None	102	(75.%)	1	-	-	-	-	-	-	-	-	-	-	-
1	140	(77.4%)	1.15	0.68-1.96	-	-	-	-	-	-	-	-	-	-
2	106	(73.6%)	0.91	0.53-1.57	-	-	-	-	-	-	-	-	-	-
3	60	(75.%)	0.95	0.50-1.82	-	-	-	-	-	-	-	-	-	-
4+	46	(83.6%)	1.65	0.73-3.73	-	-	-	-	-	-	-	-	-	-
**Intermediate Determinants**														
Knowledge about HIV risks														
Good	395	(76.9%)	1.28	0.75,2.18	-	-	-	-	-	-	-	-	-	-
Poor	59	(72.%)	1	-	-	-	-	-	-	-	-	-	-	-
Risk perception for HIV infection														
Own high-risk behaviour	55	(87.3%)	4.53***	2.06,9.93	-	-	-	-	2.49*	1.06,5.84	2.47*	1.01,6.02	3.13*	1.22,8.08
Partner(s)' high-risk behaviour	136	(97.1%)	22.46***	8.05,62.68	-	-	-	-	16.57***	5.28,51.93	16.58***	5.29,51.98	18.51***	5.77,59.35
Other reasons	66	(88.%)	4.78***	2.29,10.01	-	-	-	-	3.22**	1.47,7.07	3.22**	1.46,7.07	3.57**	1.59,7.99

For peer review only

	None	197	(62.%)	1	-	-	-	-	-	1	-	1	-	1	-
Knowing PLHIV / died from HIV															
	0	53	(58.9%)	1	-	-	-	-	-	1	-	1	-	1	-
	1 - 2	85	(75.9%)	2.23**	1.21,4.09	-	-	-	-	1.70	0.82,3.52	1.7	0.82,3.52	1.78	0.84,3.75
	3 - 4	74	(79.6%)	2.69**	1.39,5.22	-	-	-	-	1.79	0.82,3.88	1.79	0.82,3.88	1.73	0.79,3.82
	5 - 6	85	(81.7%)	3.15***	1.63,6.08	-	-	-	-	2.01+	0.93,4.34	2.01+	0.93,4.34	2.17+	0.98,4.80
	7	157	(79.7%)	2.72***	1.56,4.73	-	-	-	-	1.18	0.60,2.33	1.18	0.60,2.33	1.18	0.59,2.37
STD symptoms in last 12 months															
	Yes	86	(84.3%)	1.80*	1.02,3.20	-	-	-	-	0.63	0.31,1.25	0.63	0.31,1.26	0.67	0.33,1.36
	No	368	(74.5%)	1	-	-	-	-	-	1	-	1	-	1	-
Sickness in last 12 months															
	HIV-related illness	95	(96.9%)	0.06***	0.02,0.21	-	-	-	-	0.14**	0.04,0.49	0.14**	0.04,0.49	0.12**	0.03,0.45
	Other illness	165	(67.6%)	0.10***	0.03,0.33	-	-	-	-	0.22*	0.06,0.78	0.22*	0.06,0.79	0.20*	0.06,0.74
	None	193	(76.6%)	1	-	-	-	-	-	1	-	1	-	1	-
Psychological distress															
	Yes	366	(75.5%)	1.23	0.74,2.05	-	-	-	-	-	-	-	-	-	-
	No	88	(79.3%)	1	-	-	-	-	-	-	-	-	-	-	-
Pregnancies in last 3 years															
	One or more	335	(75.5%)	1.29	0.78,2.13	-	-	-	-	-	-	-	-	-	-
	None	119	(78.3%)	1	-	-	-	-	-	-	-	-	-	-	-
Travel time to HIV testing facility															
	<30 mins	106	(83.5%)	1	1.00,1.00	-	-	-	-	1	-	1	-	1	-
	30 - 59 mins	113	(76.4%)	0.64	0.35,1.16	-	-	-	-	0.58	0.29,1.12	0.58	0.29,1.13	0.50+	0.25,1.01
	60 - 89 mins	91	(74.%)	0.57	0.30,1.05	-	-	-	-	0.53+	0.26,1.06	0.53+	0.26,1.06	0.39*	0.18,0.84
	90 mins	142	(82.1%)	0.9	0.49,1.66	-	-	-	-	0.81	0.41,1.61	0.82	0.41,1.62	0.65	0.31,1.33
	Uncertain	2	(8.%)	0.02***	0.00,0.08	-	-	-	-	0.03***	0.01,0.13	0.03***	0.01,0.13	0.02***	0.00,0.10
Knowledge of ART															
	Yes	345	(83.5%)	3.47***	2.33,5.16	-	-	-	-	1.21	0.74,1.98	1.21	0.74,1.98	1.07	0.63,1.82

For peer review only

No	108	(59.7%)	1	-	-	-	-	-	1	-	1	-	1	-
Stigma and discrimination (in the community)														
Yes	90	(79.7%)	1.27	0.77,2.11	-	-	-	-	-	-	-	-	-	-
No	363	(75.3%)	1	-	-	-	-	-	-	-	-	-	-	-

11

For peer review only

Supplementary References

1. Albarracín D, Gillette JC, Earl AN, Glasman LR, Durantini MR, Ho M-H. A Test of Major Assumptions About Behavior Change: A Comprehensive Look at the Effects of Passive and Active HIV-Prevention Interventions Since the Beginning of the Epidemic. Psychol Bull. 2005;131: 856–897. doi:10.1037/0033-2909.131.6.856

2. Shannon K, Goldenberg SM, Deering KN, Strathdee SA. HIV infection among female sex workers in concentrated and high prevalence epidemics: why a structural determinants framework is needed. Curr Opin HIV AIDS. 2014;9: 174–182. doi:10.1097/COH.0000000000000042

3. Kaufman MR, Cornish F, Zimmerman RS, Johnson BT. Health Behavior Change Models for HIV Prevention and AIDS Care: Practical Recommendations for a Multi-Level Approach. J Acquir Immune Defic Syndr 1999. 2014;66: S250–S258. doi:10.1097/QAI.0000000000000236

4. Johnson BT, Redding CA, DiClemente RJ, Mustanski BS, Dodge BM, Sheeran P, et al. A Network-Individual-Resource Model for HIV Prevention. AIDS Behav. 2010;14: 204–221. doi:10.1007/s10461-010-9803-z

5. Shannon K, Strathdee SA, Goldenberg SM, Duff P, Mwangi P, Rusakova M, et al. Global epidemiology of HIV among female sex workers: influence of structural determinants. The Lancet. 2015;385: 55–71. doi:10.1016/S0140-6736(14)60931-4

6. Govindasamy D, Ford N, Kranzer K. Risk factors, barriers and facilitators for linkage to antiretroviral therapy care: a systematic review. AIDS Lond Engl. 2012;26: 2059–2067. doi:10.1097/QAD.0b013e3283578b9b

7. Beattie TSH, Bhattacharjee P, Suresh M, Isac S, Ramesh BM, Moses S. Personal, interpersonal and structural challenges to accessing HIV testing, treatment and care services among female sex workers, men who have sex with men and transgenders in Karnataka state, South India. J Epidemiol Community Health. 2012;66 Suppl 2: ii42-48. doi:10.1136/jech-2011-200475

8. Desmond N, Allen CF, Clift S, Justine B, Mzugu J, Plummer ML, et al. A typology of groups at risk of HIV/STI in a gold mining town in north-western Tanzania. Soc Sci Med 1982. 2005;60: 1739–1749. doi:10.1016/j.socscimed.2004.08.027

9. Camlin CS, Kwena ZA, Dworkin SL, Cohen CR, Bukusi EA. “She mixes her business”: HIV transmission and acquisition risks among female migrants in western Kenya. Soc Sci Med 1982. 2014;102: 146–156. doi:10.1016/j.socscimed.2013.11.004

10. McGrath N, Eaton JW, Newell M-L, Hosegood V. Migration, sexual behaviour, and HIV risk: a general population cohort in rural South Africa. Lancet HIV. 2015;2: e252–e259. doi:10.1016/S2352-3018(15)00045-4

11. Rai T, Lambert HS, Ward H. Complex routes into HIV care for migrant workers: a qualitative study from north India. AIDS Care. 2015;27: 1418–1423. doi:10.1080/09540121.2015.1114988

For peer review only

12. Baral S, Beyrer C, Muessig K, Poteat T, Wirtz AL, Decker MR, et al. Burden of HIV among female sex workers in low-income and middle-income countries: a systematic review and meta-analysis. Lancet Infect Dis. 2012;12: 538–549. doi:10.1016/S1473-3099(12)70066-X

13. NSWP. Stigma and Discrimination Experienced by Sex Workers Living with HIV [Internet]. 2015 Dec. Available: http://www.nswp.org/resource/stigma-and-discrimination-experienced-sex-workers-living-hiv

14. Lim S, Peitzmeier S, Cange C, Papworth E, LeBreton M, Tamoufe U, et al. Violence against female sex workers in Cameroon: accounts of violence, harm reduction, and potential solutions. J Acquir Immune Defic Syndr 1999. 2015;68 Suppl 2: S241-247. doi:10.1097/QAI.0000000000000440

15. Tounkara FK, Diabaté S, Guédou FA, Ahoussinou C, Kintin F, Zannou DM, et al. Violence, condom breakage and HIV infection among female sex workers in Benin, West Africa. Sex Transm Dis. 2014;41: 312–318. doi:10.1097/OLQ.0000000000000114

16. O'Brien S, Broom A. Gender, culture and changing attitudes: experiences of HIV in Zimbabwe. Cult Health Sex. 2013;15: 583–597. doi:10.1080/13691058.2013.776111

17. Elmes J, Skovdal M. A reconfiguration of the sex trade: How social and structural changes in eastern Zimbabwe left women involved in sex work and transactional sex more vulnerable. PLOS ONE. (accepted).

18. Mtetwa S, Busza J, Chidiya S, Mungofa S, Cowan F. "You are wasting our drugs": health service barriers to HIV treatment for sex workers in Zimbabwe. BMC Public Health. 2013;13: 698. doi:10.1186/1471-2458-13-698

19. Scorgie F, Nakato D, Harper E, Richter M, Maseko S, Nare P, et al. "We are despised in the hospitals": sex workers' experiences of accessing health care in four African countries. Cult Health Sex. 2013;15: 450–465. doi:10.1080/13691058.2012.763187

20. Govindasamy D, Meghij J, Negussi EK, Baggaley RC, Ford N, Kranzer K. Interventions to improve or facilitate linkage to or retention in pre-ART (HIV) care and initiation of ART in low- and middle-income settings – a systematic review. J Int AIDS Soc. 2014;17. doi:10.7448/IAS.17.1.19032

21. Campbell C, Scott K, Nhamo M, Nyamukapa C, Madanhire C, Skovdal M, et al. Social capital and HIV Competent Communities: The role of community groups in managing HIV/AIDS in rural Zimbabwe. AIDS Care. 2013;25: S114–S122. doi:10.1080/09540121.2012.748170

22. Campbell C, Cornish F. How Can Community Health Programmes Build Enabling Environments for Transformative Communication? Experiences from India and South Africa. AIDS Behav. 2012;16: 847–857. doi:10.1007/s10461-011-9966-2

23. Kerrigan DL, Fonner VA, Stromdahl S, Kennedy CE. Community empowerment among female sex workers is an effective HIV prevention intervention: a systematic review of the peer-reviewed evidence from low- and middle-income countries. AIDS Behav. 2013;17: 1926–1940. doi:10.1007/s10461-013-0458-4

For peer review only

For peer review only - http://bmjopen.bmj.com/site/about/guidelines.xhtml

24. Batona G, Gagnon M-P, Simonyan DA, Guedou FA, Alary M. Understanding the intention to undergo regular HIV testing among female sex workers in Benin: a key issue for entry into HIV care. J Acquir Immune Defic Syndr 1999. 2015;68 Suppl 2: S206-212. doi:10.1097/QAI.0000000000000452

25. Siziya S, Marowa E, Mbengeranwa L, Latiff A. Sexually transmitted diseases in Zimbabwe: A qualitative analysis of factors associated with choice of a health care facility. Afr Health Sci. 2005;5: 114–118.

26. Freeman EE, Weiss HA, Glynn JR, Cross PL, Whitworth JA, Hayes RJ. Herpes simplex virus 2 infection increases HIV acquisition in men and women: systematic review and meta-analysis of longitudinal studies. AIDS Lond Engl. 2006;20: 73–83.

27. Atashili J, Poole C, Ndumbe PM, Adimora AA, Smith JS. Bacterial vaginosis and HIV acquisition: A meta-analysis of published studies. AIDS Lond Engl. 2008;22: 1493–1501. doi:10.1097/QAD.0b013e3283021a37

28. Elmes J, Nhongo K, Ward H, Hallett T, Nyamukapa C, White PJ, et al. The Price of Sex: Condom Use and the Determinants of the Price of Sex Among Female Sex Workers in Eastern Zimbabwe. J Infect Dis. 2014;210: S569–S578. doi:10.1093/infdis/jiu493

29. Goh CC, Kang SJ, Sawada Y. How did Korean households cope with negative shocks from the financial crisis? J Asian Econ. 2005;16: 239–254. doi:10.1016/j.asieco.2005.01.006

30. Tlhajoane M, Eaton JW, Takaruza A, Rhead R, Maswera R, Schur N, et al. Prevalence and Associations of Psychological Distress, HIV Infection and HIV Care Service Utilization in East Zimbabwe. AIDS Behav. 2017; 1–11. doi:10.1007/s10461-017-1705-x

31. Hatcher A, Smout E, Turan J, Christofides N, Stockl H. Intimate partner violence and engagement in HIV care and treatment among women: a systematic review and meta-analysis. AIDS. 2015;

32. Duff P, Goldenberg S, Deering K, Montaner J, Nguyen P, Dobrer S, et al. Barriers to Viral Suppression Among Female Sex Workers: Role of Structural and Intimate Partner Dynamics. JAIDS J Acquir Immune Defic Syndr. 2016;73. Available: http://journals.lww.com/jaids/Fulltext/2016/09010/Barriers_to_Viral_Suppression_Among_Female_Sex.12.aspx

33. Shahmanesh M, Patel V, Mabey D, Cowan F. Effectiveness of interventions for the prevention of HIV and other sexually transmitted infections in female sex workers in resource poor setting: a systematic review. Trop Med Int Health. 2008;13: 659–679. doi:10.1111/j.1365-3156.2008.02040.x

34. Medley A, Kennedy C, O'Reilly K, Sweat M. Effectiveness of Peer Education Interventions for HIV Prevention in Developing Countries: A Systematic Review and Meta-Analysis. AIDS Educ Prev. 2009;21: 181–206. doi:10.1521/aeap.2009.21.3.181

For peer review only - http://bmjopen.bmj.com/site/about/guidelines.xhtml

35. Campbell C, Cornish F. Towards a "fourth generation" of approaches to HIV/AIDS management: creating contexts for effective community mobilisation. AIDS Care. 2010;22: 1569–1579. doi:10.1080/09540121.2010.525812

36. Busza J, Walker D, Hairston A, Gable A, Pitter C, Lee S, et al. Community-based approaches for prevention of mother to child transmission in resource-poor settings: a social ecological review. J Int AIDS Soc Vol 15 Suppl 2 2012. 2012; Available: http://www.jiasociety.org/jias/index.php/jias/article/view/17373/688

37. Mugavero MJ, Amico KR, Horn T, Thompson MA. The State of Engagement in HIV Care in the United States: From Cascade to Continuum to Control. Clin Infect Dis. 2013;57: 1164–1171. doi:10.1093/cid/cit420

38. Patel V, Simunyu E, Gwanzura F, Lewis G, Mann A. The Shona Symptom Questionnaire: the development of an indigenous measure of common mental disorders in Harare. Acta Psychiatr Scand. 1997;95: 469–475.

For peer review only

STROBE Statement—checklist of items that should be included in reports of observational studies

	Item No.	Recommendation	Page No.	Relevant line no. from manuscript
**Title and abstract**	1	(*a*) Indicate the study's design with a commonly used term in the title or the abstract	2	40-41
		(*b*) Provide in the abstract an informative and balanced summary of what was done and what was found	2	40-55
**Introduction**				
Background/rationale	2	Explain the scientific background and rationale for the investigation being reported	4-5	77-102
Objectives	3	State specific objectives, including any prespecified hypotheses	5	104-112
**Methods**				
Study design	4	Present key elements of study design early in the paper	6	117-126
Setting	5	Describe the setting, locations, and relevant dates, including periods of recruitment, exposure, follow-up, and data collection	6-8	131-169
Participants	6	(*a*) *Cohort study*—Give the eligibility criteria, and the sources and methods of selection of participants. Describe methods of follow-up		
		Case-control study—Give the eligibility criteria, and the sources and methods of case ascertainment and control selection. Give the rationale for the choice of cases and controls		
		Cross-sectional study—Give the eligibility criteria, and the sources and methods of selection of participants	7	155-161
		(*b*) *Cohort study*—For matched studies, give matching criteria and number of exposed and unexposed	NA	
		Case-control study—For matched studies, give matching criteria and the number of controls per case		
Variables	7	Clearly define all outcomes, exposures, predictors, potential confounders, and effect modifiers. Give diagnostic criteria, if applicable	8-10	171-227
Data sources/ measurement	8*	For each variable of interest, give sources of data and details of methods of assessment (measurement). Describe comparability of assessment methods if there is more than one group	8-10	171-227
Bias	9	Describe any efforts to address potential sources of bias	7,8	146-148,172-179
Study size	10	Explain how the study size was arrived at	Reference to	132-135, 157-158

For peer review only - http://bmjopen.bmj.com/site/about/guidelines.xhtml

			other papers detailing methods on p 6-7	
Quantitative variables	11	Explain how quantitative variables were handled in the analyses. If applicable, describe which groupings were chosen and why	8-10	171-227
Statistical methods	12	(*a*) Describe all statistical methods, including those used to control for confounding	10-11	230-240
		(*b*) Describe any methods used to examine subgroups and interactions	10-11	230-240
		(*c*) Explain how missing data were addressed	8	178-179 (Missingness was low [<2%] and we were able to conduct a complete case analysis)
		(*d*) *Cohort study*—If applicable, explain how loss to follow-up was addressed<br>*Case-control study*—If applicable, explain how matching of cases and controls was addressed<br>*Cross-sectional study*—If applicable, describe analytical methods taking account of sampling strategy		Probabilistic matching of targeted survey respondents with records in general population survey (165-166, 176-179)
		(*e*) Describe any sensitivity analyses	NA	NA
**Results**				
Participants	13*	(a) Report numbers of individuals at each stage of study—eg numbers potentially eligible, examined for eligibility, confirmed eligible, included in the study, completing follow-up, and analysed	12	243-249
		(b) Give reasons for non-participation at each stage	12	243-249
		(c) Consider use of a flow diagram	NA	NA
Descriptive data	14*	(a) Give characteristics of study participants (eg demographic, clinical, social) and information on exposures and potential confounders	13 (see also table 1)	275-284 and table 1
		(b) Indicate number of participants with missing data for each variable of interest	Table 1	
		(c) *Cohort study*—Summarise follow-up time (eg, average and total amount)		
Outcome data	15*	*Cohort study*—Report numbers of outcome events or summary measures over time		
		Case-control study—Report numbers in each exposure category, or summary measures of		

For peer review only - http://bmjopen.bmj.com/site/about/guidelines.xhtml

		exposure		
		Cross-sectional study—Report numbers of outcome events or summary measures	12-13	258-269
Main results	16	(*a*) Give unadjusted estimates and, if applicable, confounder-adjusted estimates and their precision (eg, 95% confidence interval). Make clear which confounders were adjusted for and why they were included	13-14	290-304, 309-316 and table 2 & 3
		(*b*) Report category boundaries when continuous variables were categorized	Table 1 p25	Tables 1,2,3
		(*c*) If relevant, consider translating estimates of relative risk into absolute risk for a meaningful time period	NA	
Other analyses	17	Report other analyses done—eg analyses of subgroups and interactions, and sensitivity analyses	13-15	290-304, 309-316
**Discussion**				
Key results	18	Summarise key results with reference to study objectives	16	322-330
Limitations	19	Discuss limitations of the study, taking into account sources of potential bias or imprecision. Discuss both direction and magnitude of any potential bias	18	370-381
Interpretation	20	Give a cautious overall interpretation of results considering objectives, limitations, multiplicity of analyses, results from similar studies, and other relevant evidence	17-18	330-368,383-393
Generalisability	21	Discuss the generalisability (external validity) of the study results	18	383-393
**Other information**				
Funding	22	Give the source of funding and the role of the funders for the present study and, if applicable, for the original study on which the present article is based	19	411-420

*Give information separately for cases and controls in case-control studies and, if applicable, for exposed and unexposed groups in cohort and cross-sectional studies.

Note: An Explanation and Elaboration article discusses each checklist item and gives methodological background and published examples of transparent reporting. The STROBE checklist is best used in conjunction with this article (freely available on the Web sites of PLoS Medicine at http://www.plosmedicine.org/, Annals of Internal Medicine at http://www.annals.org/, and Epidemiology at http://www.epidem.com/). Information on the STROBE Initiative is available at www.strobe-statement.org.

For peer review only

For peer review only - http://bmjopen.bmj.com/site/about/guidelines.xhtml

BMJ Open

Do Female Sex Workers have Lower Uptake of HIV Treatment Services than Non-Sex-Workers? A Cross-sectional Study from East Zimbabwe

Journal:	*BMJ Open*
Manuscript ID	bmjopen-2017-018751.R1
Article Type:	Research
Date Submitted by the Author:	01-Nov-2017

Complete List of Authors:	Rhead, Rebecca; Imperial College London Department of Infectious Disease Epidemiology<br>Elmes, Jocelyn; Imperial College London Department of Infectious Disease Epidemiology<br>Otobo, Eloghene; Imperial College London Department of Infectious Disease Epidemiology<br>Nhongo, Kundai; Biomedical Research and Training Institute<br>Takaruza, Albert; Biomedical Research and Training Institute<br>White, Peter; Imperial College London, MRC Centre for Outbreak Analysis and Modelling and NIHR Health Protection Research Unit in Modelling Methodology, Department of Infectious Disease Epidemiology; Public Health England, Modelling & Economics Unit, National Infection Service, Public Health England<br>Nyamukapa, Constance; Biomedical Research and Training Institute; Imperial College London Department of Infectious Disease Epidemiology<br>Gregson, Simon; Imperial College London Department of Infectious Disease Epidemiology

**Primary Subject Heading**:	HIV/AIDS
Secondary Subject Heading:	Research methods, Public health, Infectious diseases, Epidemiology
Keywords:	HIV & AIDS < INFECTIOUS DISEASES, Epidemiology < INFECTIOUS DISEASES, Public health < INFECTIOUS DISEASES

SCHOLARONE™
Manuscripts

Do Female Sex Workers have Lower Uptake of HIV Treatment Services than Non-Sex-Workers? A Cross-sectional Study from East Zimbabwe

Rebecca RHEAD[a*] and Jocelyn ELMES[a*], Eloghene OTOBO[a], Kundai NHONGO[b], Albert TAKARUZA[b], Peter J WHITE[a,c,d], Constance NYAMUKAPA[a,b], Simon GREGSON[a]

[a]Department of Infectious Disease Epidemiology, Imperial College London School of Public Health, London, United Kingdom,
[b]Biomedical Research and Training Institute, Harare, Zimbabwe.
[c]MRC Centre for Outbreak Analysis and Modelling and NIHR Health Protection Research Unit in Modelling Methodology, Imperial College London School of Public Health, London, United Kingdom
[d]Modelling and Economics Unit, National Infection Service, Public Health England, London, United Kingdom

These authors have contributed equally to the work.

Author for correspondence and reprint requests: Rebecca Rhead, Department of Infectious Disease Epidemiology, Imperial College London School of Public Health, London, United Kingdom. E-mail: r.rhead@imperial.ac.uk

Source of financial support: Wellcome Trust, MRC, NIHR

Short title: HTC and ART uptake in Zimbabwean sex workers

Key words: Female sex workers, HIV prevalence, HIV treatment cascade, determinants, Zimbabwe.

Word Count: 3512

For peer review only

Abstract

Objective: Globally, HIV disproportionately affects female sex workers (FSW) yet HIV treatment coverage is suboptimal. To improve uptake of HIV services by FSW, it is important to identify potential inequalities in access and utilisation of care and their determinants. Our aim is to investigate HIV treatment cascades for FSW and non-sex-workers (NSW) in Manicaland province, Zimbabwe, and to examine the socio-demographic characteristics and intermediate determinants that might explain differences in service uptake.

Methods: Data from a household survey conducted in 2009-2011 and a parallel snowball sample survey of FSW were matched using probability methods to reduce under-reporting of FSWs. HIV treatment cascades were constructed and compared for FSW (n=174) and NSW (n=2,555). Determinants of service uptake were identified *a priori* in a theoretical framework and tested using logistic regression.

Results: HIV prevalence was higher in FSW than in NSW (52.6% *versus* 19.8%; age-adjusted odds ratio [AOR] 4.0; 95% CI 2.9-5.5). In HIV-positive women, FSW were more likely to have been diagnosed (58.2% *versus* 42.6%; AOR=1.62; 1.02-2.59) and HIV diagnosed FSW were more likely to initiate ART (84.9% *versus* 64.0%; AOR=2.33; 1.03-5.28). No difference was found for ART adherence (91.1% *versus* 90.5%; p=0.9). FSW's greater uptake of HIV treatment services became non-significant after adjusting for intermediate factors including HIV knowledge and risk perception, travel time to services, physical and mental health, and recent pregnancy.

Conclusion: FSW are more likely to take up testing and treatment services and were closer to achieving optimal outcomes along the cascade compared to NSW. However, ART coverage was low in all women at the time of the survey. FSW's need for, knowledge of, and proximity to HIV testing and treatment facilities appears to increase uptake.

For peer review only - http://bmjopen.bmj.com/site/about/guidelines.xhtml

Strengths and Limitations of this Study

1. We provide novel insight into differential uptake of HIV treatment services for FSW and NSW in Manicaland province, Zimbabwe, and the personal, social and structural factors associated with these inequalities.
2. We use data taken from a Manicaland household survey and a parallel snowball sample survey of FSW, thus drawing on the strengths of population surveys and targeted approaches for hard-to-reach populations.
3. Our study is unique in that it compares uptake of HIV testing and ART in representative samples of FSW and NSW from the same population - we are unaware of previous studies which have done this.
4. A limitation of our study is that our data was gathered between 2009-2011.

For peer review only - http://bmjopen.bmj.com/site/about/guidelines.xhtml

Introduction

Achieving high antiretroviral treatment (ART) uptake for PLHIV is key to ending the HIV epidemic worldwide (1–3). Though UNAIDS has set ambitious "90-90-90" targets for the HIV care cascade (i.e. HIV diagnosis, ART initiation and ART adherence – as a proxy for viral load suppression) (4), these are national-level targets, and it is necessary to consider how they can be implemented for key populations such as female sex workers (FSW) – women who engage in commercial sex work or who exchange sex for goods or services(5). HIV prevalence among FSW in sub-Saharan Africa is estimated to be 10–20 times higher than in women in the general population (6). Adequate access to HIV treatment for FSW has the potential to improve the survival and health of FSW, to reduce the risk of transmission to their partners, and to potentially alter population-level HIV incidence(7). Therefore reaching and exceeding UNAIDS targets amongst FSW should be a primary objective for all national HIV control programmes (8).

Such large disparities in health between FSW and non-sex worker (NSW) women support the need for specialist sex worker services, yet treatment coverage for FSW remains poorly characterised (9)(10). It is also unclear whether inequalities for service access exist and at what stage of the HIV treatment cascade to focus more effort in driving uptake. Whilst stigma, marginalization, and abuse of human rights have all been highlighted as significant barriers that can prevent FSW from accessing HIV testing and treatment services (11), relatively few studies exist on HIV treatment cascades amongst representative samples of FSW. These include a study by Cowan and colleagues (2013) in three urban sites in Zimbabwe (Victoria Falls, Hwange and Mutare) where 50-70% were seropositive, of whom only 50% had been diagnosed. Of those diagnosed, 50-70% had been initiated onto

For peer review only - http://bmjopen.bmj.com/site/about/guidelines.xhtml

treatment, but due to the low rate of diagnosis, only 25-35% of *all* seropositive FSW in the study had received ART (12). Still, very little is known about FSW in more rural settings, or about how FSW's use of HIV services compares with that of non-sex-workers (NSW) living in the same areas. A further unknown is the extent to which differences in heath service uptake between FSW and NSW reflect largely psychosocial factors resulting from involvement in sex work (e.g. personal risk perception) as distinct from background socio-demographic factors associated with being involved in sex work in the first place (e.g. marriage breakdown).

This study has the following aims: 1) to construct and compare HIV treatment cascades for FSW and NSW in a common, rural population; 2) to identify the background socio-demographic characteristics associated with involvement in commercial sex work in this population; and 3) to identify the intermediate factors that might explain differences in health service uptake (testing and treatment) between FSW and NSW. To achieve these aims, we develop a new theoretical framework and test hypothesised determinants based on this framework using a unique data set which combines data from a general population household survey in four locations in Manicaland province, east Zimbabwe, with data from a parallel study of local FSW conducted in the same locations using snowball sampling.

For peer review only - http://bmjopen.bmj.com/site/about/guidelines.xhtml

Methods

Theoretical framework

Influenced by Boerma and Weir's proximate determinants model of HIV infection and mortality (13,14) and structural determinants frameworks of HIV among sex workers (15), we developed a theoretical framework to explain the roles that involvement in sex work and its consequences can play in mediating associations between underlying socio-demographic characteristics and use of HIV testing and treatment services (Figure 1 and Supplementary Figure 1). It is hypothesized that, within any given socio-cultural context, underlying socio-demographic characteristics contribute to whether or not a woman engages in sex work which may, in turn, alter her pattern of use of HIV services. In the framework, sex work is hypothesized to influence use of HIV services primarily through its effects on intermediate determinants that exist in four domains: personal, interpersonal, social and structural.

Figure 1: Theoretical framework

Data

Data for this study were taken from the Manicaland HIV/STD Prevention Project (Manicaland study) (16) and the Manicaland Women at Risk Study (WR study) (17). The Manicaland study is an open-cohort general-population survey which examines the dynamics of HIV transmission and its impact in 12 sites in Manicaland province in eastern Zimbabwe (*http://www.manicalandhivproject.org/*). These sites represent four of the main socio-economic strata in Manicaland: small towns, agricultural estates, roadside trading centres, and subsistence farming villages. Topics covered in individual interviews included

For peer review only - http://bmjopen.bmj.com/site/about/guidelines.xhtml

socio-economic characteristics, sexual behaviour, psychosocial characteristics, and use of HIV testing and treatment services. Participants were also requested to provide a dried blood sample (DBS) for HIV sero-testing. The data used in this analysis were taken from the 5th round of the Manicaland survey (October 2009 - July 2011) and were restricted to the four sites (one in each socio-economic stratum) also covered by the WR study.

The data from the Manicaland study were linked with data from the WR study, a parallel targeted cohort study conducted to identify women at heightened risk of HIV infection through exchange of sex (including sex work), to enhance detection of FSW and to permit comparison of HIV treatment cascades between FSW and NSW within a common wider population. The WR study is a research project, conducted in four of the same sites covered in the Manicaland study, which aimed to explore the sexual behaviours of women at heightened risk of HIV infection (*http://www.manicalandhivproject.org/women-at-risk.html*). Data for the WR study were collected between March 2010 and July 2011 using a combination of PLACE (Priorities for Local AIDS Control Effort, a form of location based sampling) (18) and snowball sampling (19) methods. Data collection procedures have been described in detail elsewhere (20) but are summarised here. PLACE involves sampling locations of known sex work activity. An inventory of locations was created based on discussions with community members. Since only a small number of venues were identified, all venues were sampled. To capture exchange sex outside of specific venues, the population was sampled using a modified respondent-driven sampling approach (21). Seeds were selected to represent the diversity of those involved in exchange sex. These seeds then recruited up to three peers that met broad eligibility criteria (women aged 18+ who had ever exchanged sex for money, goods, or favours) and were compensated with one bar of laundry soap per respondent referred and invited to

For peer review only - http://bmjopen.bmj.com/site/about/guidelines.xhtml

interview. We mitigated duplication and impersonation by cross-referencing names of nominated individuals with the names of women appearing to interview and by close monitoring by key informants (women with personal experience of sex work or who worked closely with women selling sex in the communities as health and support workers).

The FSW who participated in both the Manicaland and WR study were requested to provide permission to link their data across both projects. Data for consenting participants were linked via probabilistic matching based on participant name, date of birth, and village name. Prior ethical approval for the Manicaland study (with the WR study included as a sub-study) was obtained from the Medical Research Council of Zimbabwe (MRCZ/A/681) and the Imperial College Research Ethics Committee (ICREC_9_3_13).

Study variables

Female sex worker: The Manicaland and WR studies contained identical indicators of sex work. Informed by prior qualitative work within study communities (22), and in line with UNAIDS definitions (23), participants in the Manicaland study were considered to be FSW if they: a) self-identified as a sex worker or prostitute; b) had ever gone to bars/beer halls to meet clients; or c) had exchanged sex for money/goods, in at least one of the two studies

Non-sex-workers: NSWs in the study were taken to be all women interviewed in the Manicaland study who reported having ever had sex and who did not meet the definition of a FSW given above based on their self-reports in the Manicaland study and/or in the WR study.

For peer review only - http://bmjopen.bmj.com/site/about/guidelines.xhtml

FSW and NSW as defined above were included in the current analysis if they were aged 15-58 years (the age-range covered by the WR study) and were resident in one of the 4 Manicaland study areas also covered by the WR study.

HIV treatment cascade: HIV diagnosis was defined as the percentage of all HIV-positive participants (based on HIV tests done in the Manicaland study) who reported ever having been tested and having collected their results and received a positive result at their most recent HIV test. ART initiation was defined as the percentage of HIV-positive participants who knew their status (denominator) and also reported taking drugs "that stop HIV from causing AIDS" (numerator). ART adherence was used as a further indicator of HIV service use and as a proxy for viral load suppression. HIV-positive participants who reported ever having started ART were included in the denominator; those who reported never having stopped or forgotten to take their medication and who reported taking ARVs regularly were included in the numerator.

Health service uptake: Two measures of health service engagement were considered as dependent variables in our regression analyses: 1) uptake of HIV testing; and 2) uptake of ART. Uptake of HIV testing was defined as ever having had an HIV test and collected the result. Uptake of treatment was measured in seropositive participants and based on reports of having taking drugs "that stop HIV from causing AIDS".

Socio-demographic characteristics: Age (24), marital status [35], socio-economic status, religion (24)(25), area of residence (24) (26), education level [15], and number of living children were considered as potential underlying determinants of involvement in sex work

For peer review only

For peer review only - http://bmjopen.bmj.com/site/about/guidelines.xhtml

and use of HIV services (Figure 1, see also more detailed explanation in Supplementary Material). For socio-economic status (SES), we used a continuous combined measure of sellable and non-sellable assets (27), divided into terciles (1=poorest → 3=richest). For religious denomination, we used Manzou's four category grouping of Manicaland churches (25).

<u>Intermediate determinants of HIV service uptake:</u> Personal factors potentially mediating HIV service uptake in the theoretical framework included: recent ill-health (self-reported experience of recent ill-health and whether or not this was believed to be HIV-related), self-reported symptoms of STDs, self-reported recent pregnancies (that could translate to HIV testing through uptake of PMTCT services), HIV knowledge (number of correct responses to four questions: 0-2 correct answers=poor knowledge, 3-4 correct answers=good knowledge), HIV risk perception (whether participants perceived they had ever been at risk of becoming infected with HIV, and if so, was it through their own risky behaviour, their partner's risky behaviour or for other reasons), awareness of treatment for HIV, and an objective mental health assessment using a locally-validated questionnaire (Shona Symptom Questionnaire, SSQ) (28) (29). Interpersonal factors included HIV salience (number of people known by the participant who are living with HIV or who had died from AIDS) and awareness of other people using ART (individuals who were unaware of ART were combined with those unaware of anyone using ART because of small numbers). Potential social and structural influences included accessibility of HTC (or ART) services; participant's awareness of a health facility offering HTC (or ART), and the estimated the travel time to the nearest health facility. Stigma was measured using two dichotomous variables: whether the participant was ever deterred from getting a test due to

For peer review only - http://bmjopen.bmj.com/site/about/guidelines.xhtml

stigma or discrimination, and whether the participant felt that PLHIV faced stigma and discrimination within the community.

Travel time and stigma relating to HTC and awareness of ART were used only in the analysis of uptake of testing (i.e. not for ART uptake). Travel time to ART services was used only in the analysis of ART uptake (i.e. not for HIV testing).

Statistical analyses

The analysis consisted of several stages. First, HIV prevalence and HIV treatment cascade outcomes were calculated and compared between FSW and NSW. Second, bivariate (age-adjusted) regression models were used to explore associations between socio-demographic characteristics and involvement in sex work, and associations between sex work and intermediate determinants of uptake hypothesised in the theoretical framework. Third, age-adjusted bivariate examination of associations between both socio-demographic characteristics and intermediate determinants and HIV testing and treatment was conducted to detect significant associations at $p<0.1$. Fourth, age-adjusted multivariable regression models were used to compare uptake of health services in FSW versus NSW – before and after inclusion of socio-demographic factors and intermediate determinants of uptake (significant at $p<0.1$). All analyses were done using Stata 14.

For peer review only

For peer review only - http://bmjopen.bmj.com/site/about/guidelines.xhtml

Results

Identification of FSW

174 participants were identified as FSW in at least one of the two studies; 132 were included from the WR study (111 were identified based on their responses to the WR study questionnaire alone and 31 also self-identified as sex workers in the Manicaland questionnaire), and 32 were identified based on their answers to the Manicaland study questionnaire alone.

A total of 3402 women aged 15-59 years participated in the Manicaland study in round five in the four sites also covered by the WR study and were not identified as FSWs in either study. These participants were all treated as being NSWs; however, 135 were excluded from the study as they were outside the age-range of the FSW in the study (19-58 years), and a further 538 were excluded because they had not started sex. This produced a total sample of 2729 (FSW=174, 6.4%; NSW=2555, 93.6%).

HIV prevalence

HIV prevalence was significantly higher in FSW (52.6%, 95%CI 45.1%-60.0%; n/N=91/173) compared to NSW (19.8%, 18.3%-21.4%; 502/2535) (age-adjusted odds ratio [AOR] 4.0; 2.9-5.5). Study HIV laboratory test results were inconclusive for 1 FSW and 2 NSWs.

HIV treatment cascades in FSW and NSW

In HIV-positive women, diagnosis (i.e. women who were aware of their HIV-positive status) was higher in FSW (58.2%, 95%CI 47.7%-68.1%; 53/91) than in NSW (42.6%,

For peer review only - http://bmjopen.bmj.com/site/about/guidelines.xhtml

38.3%-47.9%; 214/502) (AOR, 1.62; 1.02-2.59; p=0.042. In HIV-negative women, there was no significant difference between FSW and NSW in uptake of HIV testing (81.7% in FSW; 75.3% in NSW; AOR, 1.40; 0.78-2.51; p=0.259).

In women diagnosed with HIV infection, initiation onto ART was higher in FSW (84.9%, 72.1%-92.4%; 45/53) than in NSW (64.2%, 57.5%-70.4%; 138/214) (AOR, 2.33; 1.03-5.28; p=0.043). No significant difference was found between FSW (91.1%, 77.9%-96.7%; 41/45) and NSW (90.5%, 84.2%-94.4%; 124/137) in self-reported adherence to ART (AOR, 1.08; 0.33-3.52; p=0.901). Overall, 49.4% (45/91) of ***all*** HIV-positive FSW reported being on and adhering to ART compared to 27.5% (139/505) of infected NSW (as shown in Figure 2a & 2b)

Figure 2a & 2b: Comparisons of HIV treatment cascades for FSW versus NSW

Socio-demographic characteristics and intermediate determinants of HIV service use associated with sex work

Table 1 shows the age-adjusted bivariate associations between sociodemographic factors and sex work, and between sex work and intermediate determinants - i.e. the first two pathways in the theoretical framework (Figure 1). Sex work was most common in women aged 30-49; single, divorced and widowed women; women with no religious affiliation; women in the two poorest terciles; women living in small towns; and women with no living children. For the intermediate determinants, sex work was associated with greater risk perception for HIV infection (particularly through personal risky behaviours); knowing at least three people with HIV; experiencing recent HIV-related illness and STD symptoms; poor mental health; no pregnancies in the past three years; short travel times to

For peer review only - http://bmjopen.bmj.com/site/about/guidelines.xhtml

HTC and ART facilities; having heard of ART; and reporting that HIV stigma and discrimination exist in the community. We also found a non-significant difference between FSW and NSW for knowledge of HIV risks.

Table 1: Associations between FSW and socio-demographic and intermediate determinants of uptake of HIV services

Socio-demographic characteristics and intermediate determinants of HIV testing

In HIV-positive and HIV-negative women combined, FSW were more likely than NSW to have ever been tested for HIV (FSW: 81.6%, NSW: 75.3%; AOR 1.50; 95%CI 1.00-2.24). Table 2 shows the bivariate and multivariable associations of socio-demographic factors and intermediate determinants on testing. In bivariate analysis, sex work was associated with ever having been tested, as are all socio-demographic factors included in this analysis. All intermediate determinants with the exception of psychological distress and stigma (after testing and in the community) are associated with testing at p<0.1. In multivariable analysis, the association between sex work and testing is strengthened after adjusting for socio-demographic factors, with sex work being associated with 75% increased odds of testing (AOR 1.75, 1.14-1.69). However, this association between sex work and testing disappears after also accounting for intermediate determinants (AOR 1.11, 0.69-1.81).

When the analysis was restricted to HIV-positive women (Table S1), the association between FSW and diagnosis approached statistical significance after adjusting for socio-demographic factors (AOR 1.83, 1.00-3.37; p=0.052).

For peer review only - http://bmjopen.bmj.com/site/about/guidelines.xhtml

Table 2: Factors contributing to the difference in uptake of HIV testing between FSW and NSW

Socio-demographic characteristics and intermediate determinants of HIV treatment

Table 3 shows the bivariate and multivariable associations between socio-demographic factors and intermediate determinants and ART initiation. In bivariate analysis, sex work is associated with 164% increased odds of ART initiation (AOR 2.64, 1.16-6.00). Older age, more urban site types, having no living children, knowing people who have / had HIV, not having psychological distress, and shorter travel times to ART were also associated with treatment initiation at p<0.1. In multivariable analysis, FSW still tended to have higher odds of ART initiation than NSW, but this association was no longer statistically significant when socio-demographic factors were accounted for (AOR 2.28, 0.97-5.39).

Table 3: Factors contributing to the difference in uptake of ART between FSW and NSW

For peer review only - http://bmjopen.bmj.com/site/about/guidelines.xhtml

Discussion

FSW had higher uptake of HIV testing and ART services than other sexually-experienced women in our study areas in east Zimbabwe. For HIV testing, this advantage strengthened after accounting for differences in background socio-demographic characteristics but disappeared after further adjustment for intermediate determinants, confirming a process of mediation hypothesised in the theoretical framework. FSW's greater knowledge about HIV and greater personal perceived risk of being HIV-positive, their better knowledge of and proximity to testing services, and their greater likelihood of perceiving HIV-related symptoms (i.e. the intermediate determinants outlined in our framework) may have contributed to their higher levels of HIV testing. Greater ART uptake in FSW compared to NSW was explained not by intermediate factors relating to sex work status but by their older ages (i.e. fewer aged 19-29 years) and lower numbers of living children. The reason for the link with small numbers of living children is not clear but, in *Shona* culture (30), subfertility/infertility can lead to divorce which, in turn, is associated with greater likelihood of involvement in sex work. Widowhood at young ages may be associated with early HIV infection, reduced fertility, high early child mortality and involvement in sex work. Also, low fertility and early child mortality can be markers for more advanced HIV infection (31); thereby increasing the likelihood of meeting the eligibility criteria for ART that pertained at the time of the study (CD4<350 or World Health Organisation phases III or IV in 2009-2011). ART adherence was similar in FSW and NSW.

HIV prevalence in FSW in east Zimbabwe (52.6%) was comparable with prevalence in FSW in other southern African countries (range: 59.6-70.7%) (6). The proportion of infected FSW who had been diagnosed (58.2%) was slightly higher than estimates for

For peer review only - http://bmjopen.bmj.com/site/about/guidelines.xhtml

FSW in urban Zimbabwean locations (50% in 2013) (12); whilst the proportion of those diagnosed who had been started on treatment (87% *versus* 0-73%) and the proportion of those on treatment who reported adhering to ART (91% *versus* 67-100% (10) (32) (33)) were also high. We are unaware of any previous studies that have compared uptake of HIV testing and ART in representative samples of FSW and NSW from the same population. However, similar levels of ART adherence have been found in Mozambique and Benin (34) (35).

The results suggest that several of the structural, interpersonal and personal factors outlined in our framework may contribute to differences in uptake of HIV testing and ART services between and amongst FSW and NSW. As noted previously by Paulin and colleagues in a rural setting in Mozambique (36), knowledge of HTC services can affect it's uptake amongst women (42%). In Manicaland, FSW had better knowledge of ART and, because they lived largely in towns, were structurally advantaged over NSW, who more often lived in areas more remote from testing and treatment facilities. In terms of uptake of HIV care, these factors appear to have offset the disadvantages that FSW face from poorer mental health and greater stigma and discrimination. Poor mental health, in the form of greater psychological distress, is associated with lower ART uptake in east Zimbabwe (29). As in many previous studies (37), we found that psychological distress was more common in sex workers but we did not find this was important factor mediating uptake. FSW in this study also reported higher levels of stigma linked to HIV than NSW; however, unlike studies elsewhere in Zimbabwe (38), we did not find stigma to be a significant deterrent to accessing healthcare. One reason may be that our study questionnaire did not include a measure of stigma specifically related to sex work. FSW were more likely to report HIV illness and be HIV positive, but perception of HIV-related

For peer review only

For peer review only - http://bmjopen.bmj.com/site/about/guidelines.xhtml

symptoms, not HIV serostatus, was associated with HIV testing. This suggests women in Manicaland, and particularly FSW, are likely to be diagnosed and prescribed treatment late, which can mean reduced survival (39) and greater HIV-related comorbidities. It could be that HIV positive NSW may have more pregnancies and therefore often get diagnosed early when still healthy, HIV positive FSW have fewer pregnancies and therefore often only get diagnosed late after becoming sick

This study utilises a unique data source that draws from the combined strengths of population surveys and chain-referral methods and allowed us to analyse a representative sample with reduced under-reporting of locally-resident FSW, and provided a rare opportunity to compare the characteristics and determinants of HIV service use for FSW and NSW from the same study areas. However, the data used were cross-sectional so we have been unable to determine the causal nature of the relationships explored in the study. Also, in comparing the HIV care cascades for FSW and NSW, we have used self-reported ART adherence as a proxy for viral suppression as biomarkers for viral load were not available (40). Finally, our study sites were not covered by FSW intervention programmes in Zimbabwe such as 'Sisters with a Voice' or the SAPPHIRE trial (http://www.ceshhar.org.zw/). This has allowed us to compare the experience of FSW and NSW in the absence of targeted interventions; however, it would also be valuable for researchers with data from areas where these interventions are being implemented to perform a comparable analysis.

In east Zimbabwe, between 2009 and 2011, FSW were more likely than NSW to have been tested for HIV infection and to have taken up ART. These findings challenge the common perception that HIV-infected FSW are marginalised from HIV treatment in the absence of

For peer review only - http://bmjopen.bmj.com/site/about/guidelines.xhtml

targeted services. However, high ART coverage in FSW is critical both for their own health and survival (with many FSW appearing to access treatment only at advanced stages of infection) and to reduce the rate of new HIV infections in the general population. Furthermore, the results of this study show that different factors influence uptake of HIV services in FSW compared to NSW. For example, whilst decentralised services (including use of recently developed sensitive and specific rapid tests to "task-shift" HIV testing to community health workers) and intensified efforts to improve personal risk perception and may increase uptake of ART in NSW, targeted services that address the stigma and discrimination associated with sex work (not measured here but shown to be an important factor in other studies (37)(41)) may be more effective for FSW. NSW would also additionally benefit from measures to improve treatment uptake once diagnosed. One possible approach for this could be to use couples' HIV testing and counselling to address the dominant inter-personal role of male spouses in determining women's HIV care that has been described in previous qualitative research in Manicaland [35]. The effect of such unmeasured influences could be reflected in the residual effect of sex work after adjusting for sociodemographic factors and intermediate determinants. Further research is needed as the situation may be changing – particularly since the introduction of universal eligibility for ART; nevertheless, recent data show that a third of HIV-infected women in Zimbabwe are not yet virally suppressed [36] so continued and enhanced efforts such as these are probably still needed to increase coverage of treatment services in both FSW and NSW.

For peer review only - http://bmjopen.bmj.com/site/about/guidelines.xhtml

Acknowledgements

Authors' contribution

RR, JE, PJW, SG, CN and KN were involved in study concept and design. CN, JE, KN and AT acquired and curated the data. JE, RR, SG and EO were involved in the design of the analysis. RR conducted the statistical analysis supervised by SG and JE. RR, SG, JE and EO interpreted the results and drafted the article.

Data Access

Data produced by the Manicaland Project can be obtained from the project website: http://www.manicalandhivproject.org/data-access.html. Here we provide a core dataset which contains a sample of socio-demographic, sexual behaviour and HIV testing variables from all 6 rounds of the main survey, as well as data used in the production of recent academic publications. If further data is required, a data request form must be completed (available to download from our website) and submitted to s.gregson@imperial.ac.uk. If the proposal is approved, we will send a data sharing agreement which must be agreed upon before we release the requested data.

Funding Sources

SG thanks the Wellcome T rust for funding (grants: 084401/Z/07/B and 090285MA). JE thanks the Medical Research Council for her PhD funding (grant number http://www.mrc.ac.uk/index.htm) and the Wellcome Trust for postdoctoral funding (grant number: 090285/Z/09/Z; http://www.wellcome.ac.uk/). PJW thanks the MRC for Centre funding (MR/K010174/1), and the UK NIHR Health Protection Research Unit in Modelling Methodology at Imperial College London in partnership with Public Health

For peer review only - http://bmjopen.bmj.com/site/about/guidelines.xhtml

483 England for funding (HPRU-2012-10080). The funders had no role in study design, data
484 collection and analysis, decision to publish, or preparation of the manuscript. The views
485 expressed are those of the authors and not necessarily those of the Department of Health,
486 MRC, NHS, NIHR, Public Health England, or the Wellcome Trust.

487

488 **Competing Interests**

489 The authors have no competing interests.

For peer review only

For peer review only - http://bmjopen.bmj.com/site/about/guidelines.xhtml

References

1. Cohen MS, Smith MK, Muessig KE, Hallett TB, Powers KA, Kashuba AD. Antiretroviral treatment of HIV-1 prevents transmission of HIV-1: where do we go from here? The Lancet. 2013 Nov 8;382(9903):1515–24.

2. Hontelez JAC, Lurie MN, Bärnighausen T, Bakker R, Baltussen R, Tanser F, et al. Elimination of HIV in South Africa through Expanded Access to Antiretroviral Therapy: A Model Comparison Study. PLOS Med. 2013 Oct 22;10(10):e1001534.

3. Collaboration TAT (ART) C. Prognostic importance of initial response in HIV-1 infected patients starting potent antiretroviral therapy: analysis of prospective studies. The Lancet. 2003 Aug 30;362(9385):679–86.

4. HIV/AIDS JUNPo. 90–90–90 - An ambitious treatment target to help end the AIDS epidemic | UNAIDS [Internet]. 2014. Available from: http://www.unaids.org/en/resources/documents/2014/90-90-90

5. Joint United Nations Programme on HIV/AIDS. UNAIDS terminology guidelines. Geneva: Joint United Nations Programme on HIV/AIDS.; 2015.

6. Baral S, Beyrer C, Muessig K, Poteat T, Wirtz AL, Decker MR, et al. Burden of HIV among female sex workers in low-income and middle-income countries: a systematic review and meta-analysis. Lancet Infect Dis. 2012 Jul;12(7):538–49.

7. Delva W, Eaton JW, Meng F, Fraser C, White RG, Vickerman P, et al. HIV Treatment as Prevention: Optimising the Impact of Expanded HIV Treatment Programmes. PLOS Med. 2012 Jul 10;9(7):e1001258.

8. Alary M, Lowndes CM, Van de Perre P, Béhanzin L, Batona G, Guédou FA, et al. Scale-up of combination prevention and antiretroviral therapy for female sex workers in West Africa: time for action. AIDS Lond Engl. 2013 Jun 1;27(9):1369–74.

9. Risher K, Mayer K, Beyrer C. The HIV treatment cascade in men who have sex with men, people who inject drugs and sex workers. Curr Opin HIV AIDS. 2015 Nov;10(6):420–9.

10. Mountain E, Mishra S, Vickerman P, Pickles M, Gilks C, Boily M-C. Antiretroviral Therapy Uptake, Attrition, Adherence and Outcomes among HIV-Infected Female Sex Workers: A Systematic Review and Meta-Analysis. PLOS ONE. 2014 Sep 29;9(9):e105645.

11. Arnott J, Crago A. Rights Not Rescue: A Report On Female, Trans and Male Sex Workers' Human Rights in Botswana, Namibia and South Africa. [Internet]. New York City; 2009. Available from: https://www.opensocietyfoundations.org/sites/default/files/summary_20081114.pdf

12. Cowan FM, Mtetwa S, Davey C, Fearon E, Dirawo J, Wong-Gruenwald R, et al. Engagement with HIV Prevention Treatment and Care among Female Sex Workers

For peer review only - http://bmjopen.bmj.com/site/about/guidelines.xhtml

in Zimbabwe: a Respondent Driven Sampling Survey. PLOS ONE. 2013 Oct 15;8(10):e77080.

13. Boerma JT, Weir SS. Integrating Demographic and Epidemiological Approaches to Research on HIV/AIDS: The Proximate-Determinants Framework. J Infect Dis. 2005 Feb 1;191(Supplement 1):S61–7.

14. Lopman B, Nyamukapa C, Mushati P, Mupambireyi Z, Mason P, Garnett GP, et al. HIV incidence in 3 years of follow-up of a Zimbabwe cohort—1998–2000 to 2001–03: contributions of proximate and underlying determinants to transmission. Int J Epidemiol. 2008 Feb 1;37(1):88–105.

15. Shannon K, Strathdee SA, Goldenberg SM, Duff P, Mwangi P, Rusakova M, et al. Global epidemiology of HIV among female sex workers: influence of structural determinants. The Lancet. 2015 Jan 3;385(9962):55–71.

16. Gregson S, Garnett GP, Nyamukapa CA, Hallett TB, Lewis JJC, Mason PR, et al. HIV decline associated with behavior change in eastern Zimbabwe. Science. 2006 Feb 3;311(5761):664–6.

17. Elmes J, Nhongo K, Ward H, Hallett T, Nyamukapa C, White PJ, et al. The Price of Sex: Condom Use and the Determinants of the Price of Sex Among Female Sex Workers in Eastern Zimbabwe. J Infect Dis. 2014 Dec 1;210(suppl 2):S569–78.

18. Weir SS, Merli MG, Li J, Gandhi AD, Neely WW, Edwards JK, et al. A comparison of respondent-driven and venue-based sampling of female sex workers in Liuzhou, China. Sex Transm Infect. 2012 Dec;88(Suppl_2):i95–101.

19. Kendall C, Kerr LRFS, Gondim RC, Werneck GL, Macena RHM, Pontes MK, et al. An empirical comparison of respondent-driven sampling, time location sampling, and snowball sampling for behavioral surveillance in men who have sex with men, Fortaleza, Brazil. AIDS Behav. 2008 Jul;12(4 Suppl):S97–104.

20. Elmes J. Women at Risk [Internet]. Women at Risk Study - Manicaland Centre for Public Health. Available from: http://www.manicalandhivproject.org/women-at-risk.html

21. Heckathorn DD. Respondent-Driven Sampling: A New Approach to the Study of Hidden Populations. Soc Probl. 1997 May 1;44(2):174–99.

22. Elmes J, Skovdal M, Nhongo K, Ward H, Campbell C, Hallett TB, et al. A reconfiguration of the sex trade: How social and structural changes in eastern Zimbabwe left women involved in sex work and transactional sex more vulnerable. PLOS ONE. 2017 Feb 22;12(2):e0171916.

23. UNAIDS. Sex work and HIV/AIDS, technical update. [Internet]. Geneva; 2002. Available from: http://data.unaids.org/publications/IRC-pub02/jc705-sexwork-tu_en.pdf

For peer review only

For peer review only - http://bmjopen.bmj.com/site/about/guidelines.xhtml

24. Govindasamy D, Ford N, Kranzer K. Risk factors, barriers and facilitators for linkage to antiretroviral therapy care: a systematic review. AIDS Lond Engl. 2012 Oct 23;26(16):2059–67.

25. Manzou R, Schumacher C, Gregson S. Temporal Dynamics of Religion as a Determinant of HIV Infection in East Zimbabwe: A Serial Cross-Sectional Analysis. PLOS ONE. 2014 Jan 20;9(1):e86060.

26. Schaefer R, Gregson S, Takaruza A, Rhead R, Masoka T, Schur N, et al. Spatial patterns of HIV prevalence and service use in East Zimbabwe: implications for future targeting of interventions. J Int AIDS Soc [Internet]. 2017 [cited 2017 May 23];20(1). Available from: http://www.jiasociety.org/index.php/jias/article/view/21409

27. Schur N, Mylne A, Mushati P, Takaruza A, Ward H, Nyamukapa C, et al. The effects of household wealth on HIV prevalence in Manicaland, Zimbabwe – a prospective household census and population-based open cohort study. J Int AIDS Soc [Internet]. 2015 Nov 20 [cited 2016 Nov 10];18(1). Available from: http://www.ncbi.nlm.nih.gov/pmc/articles/PMC4655223/

28. Patel V, Simunyu E, Gwanzura F, Lewis G, Mann A. The Shona Symptom Questionnaire: the development of an indigenous measure of common mental disorders in Harare. Acta Psychiatr Scand. 1997 Jun;95(6):469–75.

29. Tlhajoane M, Eaton JW, Takaruza A, Rhead R, Maswera R, Schur N, et al. Prevalence and Associations of Psychological Distress, HIV Infection and HIV Care Service Utilization in East Zimbabwe. AIDS Behav. 2017 Feb 13;1–11.

30. Bourdillon M. The Shona peoples: An ethnography of the contemporary Shona, with special reference to their religion. 3rd ed. Gweru: Mambo Press; 1987.

31. Ross A, Van der Paal L, Lubega R, Mayanja BN, Shafer LA, Whitworth J. HIV-1 disease progression and fertility: the incidence of recognized pregnancy and pregnancy outcome in Uganda. AIDS Lond Engl. 2004 Mar 26;18(5):799–804.

32. Braunstein SL, Umulisa M-M, Veldhuijzen NJ, Kestelyn E, Ingabire CM, Nyinawabega J, et al. HIV diagnosis, linkage to HIV care, and HIV risk behaviors among newly diagnosed HIV-positive female sex workers in Kigali, Rwanda. J Acquir Immune Defic Syndr 1999. 2011 Aug 1;57(4):e70–6.

33. Bassett IV, Regan S, Chetty S, Giddy J, Uhler LM, Holst H, et al. Who starts antiretroviral therapy in Durban, South Africa?… not everyone who should. AIDS Lond Engl. 2010 Jan;24(Suppl 1):S37–44.

34. Huet C, Ouedraogo A, Konaté I, Traore I, Rouet F, Kaboré A, et al. Long term virological, immunological and mortality outcomes in a cohort of HIV-infected female sex workers treated with highly active antiretroviral therapy in Africa. BMC Public Health. 2011;11:700.

35. Diabaté S, Zannou DM, Geraldo N, Chamberland A, Akakpo J, Ahouada C, et al. Antiretroviral Therapy among HIV-1 Infected Female Sex Workers in Benin: A

For peer review only

For peer review only - http://bmjopen.bmj.com/site/about/guidelines.xhtml

Comparative Study with Patients from the General Population. World J AIDS. 2011 Sep 29;01(03):94.

36. Paulin HN, Blevins M, Koethe JR, Hinton N, Vaz LM, Vergara AE, et al. HIV testing service awareness and service uptake among female heads of household in rural Mozambique: results from a province-wide survey. BMC Public Health [Internet]. 2015 Feb 12 [cited 2017 Feb 8];15. Available from: http://www.ncbi.nlm.nih.gov/pmc/articles/PMC4339241/

37. NSWP. Stigma and Discrimination Experienced by Sex Workers Living with HIV [Internet]. 2015 Dec. Available from: http://www.nswp.org/resource/stigma-and-discrimination-experienced-sex-workers-living-hiv

38. Mtetwa S, Busza J, Chidiya S, Mungofa S, Cowan F. 'You are wasting our drugs': health service barriers to HIV treatment for sex workers in Zimbabwe. BMC Public Health. 2013 Jul 31;13:698.

39. Kitahata MM, Gange SJ, Abraham AG, Merriman B, Saag MS, Justice AC, et al. Effect of Early versus Deferred Antiretroviral Therapy for HIV on Survival. N Engl J Med. 2009 Apr 30;360(18):1815–26.

40. Rhead R, Masimirembwa C, Cooke G, Takaruza A, Nyamukapa C, Mutsimhi C, et al. Might ART Adherence Estimates Be Improved by Combining Biomarker and Self-Report Data? PloS One. 2016;11(12):e0167852.

41. Getnet M, Damen H. Level of stigma among female sex workers: comparison of two surveys of HIV behavioral data, Ethiopia. Afr Health Sci. 2011 Dec;11(4):543–9.

For peer review only

For peer review only - http://bmjopen.bmj.com/site/about/guidelines.xhtml

Figures

624 **Figure 1: Theoretical framework illustrating how engaging in sex work (or not) may influence use of HIV testing and treatment services.**

627 **Figures 2a and 2b: Comparison of HIV treatment cascades (non-cumulative and cumulative) for female sex workers and non-sex workers in Manicaland, Zimbabwe, 2009-2011.**

For peer review only

For peer review only - http://bmjopen.bmj.com/site/about/guidelines.xhtml

Tables

Table 1: Socio-demographic characteristics associated with female involvement in sex work, and associations between sex work and intermediate determinants of HIV testing and treatment, Manicaland Zimbabwe, 2009-2011

	FSW		NSW				
Socio-demographic characteristic	n	%	n	%	N	AOR	95% CI
Age-group							
19-29	36	(20.7%)	950	(37.2%)	986	1	-
30-39	74	(42.5%)	710	(27.8%)	784	2.75	1.83-4.14
40-49	46	(26.4%)	527	(20.6%)	573	2.3	1.47-3.61
50-58	18	(10.3%)	368	(14.4%)	386	1.29	0.72-2.30
Marital status							
Never married	8	(4.6%)	68	(2.7%)	76	3.18	1.46-6.92
Married	87	(50.0%)	1912	(74.8%)	1999	1	-
Divorced or separated	41	(23.6%)	225	(8.8%)	266	3.76	2.52-5.62
Widowed	37	(21.3%)	350	(13.7%)	387	2.25	1.47-3.45
Church Denomination							
Christian	89	(51.1%)	1385	(54.2%)	1474	1	-
Spiritual	53	(30.5%)	882	(34.5%)	935	0.91	0.64-1.30
Other	22	(12.6%)	253	(9.9%)	275	1.35	0.83-2.21
None	10	(5.7%)	33	(1.3%)	43	4.47	2.10-9.52
Socio-economic status							
First (poorest) tercile	121	(69.5%)	1635	(64.0%)	1756	1	-
Second tercile	42	(24.1%)	572	(22.4%)	614	0.97	0.67-1.40
Third tercile	7	(4.0%)	263	(10.3%)	270	0.37	0.17-0.80
Residential area							
Town	64	(36.8%)	597	(23.4%)	661	1	-
Agricultural estate	40	(23.0%)	610	(23.9%)	650	0.59	0.39-0.89
Roadside settlement	45	(25.9%)	702	(27.5%)	747	0.59	0.40-0.89
Subsistence farming village	25	(14.4%)	646	(25.3%)	671	0.36	0.22-0.58
Education							
Primary or none	74	(42.5%)	944	(36.9%)	1018	1	-
Secondary or higher	100	(57.5%)	1611	(63.1%)	1711	0.78	0.54-1.11
Children alive							
None	44	(25.3%)	365	(14.3%)	409	1	-
1	45	(25.9%)	750	(29.4%)	795	0.54	0.34-0.83
2	43	(24.7%)	701	(27.4%)	744	0.46	0.29-0.72
3	20	(11.5%)	436	(17.1%)	456	0.27	0.15-0.47
4	22	(12.6%)	303	(11.9%)	325	0.39	0.22-0.67
Intermediate determinants							
**HIV testing**							
HIV Result							
Positive	91	(52.3%)	505	(19.8%)	596	4.00	2.90-5.50
Negative	82	(47.1%)	2048	(80.2%)	2130	1	-
Knowledge about HIV risks							
Good	158	(90.8%)	2167	(84.8%)	2325	1.63	0.96-2.76
Poor	16	(9.2%)	388	(15.2%)	404	1	-
Knowing persons living with or who PLHIV / died from HIV							
0	14	(8.0%)	506	(19.8%)	520	1	-
1 - 2	22	(12.6%)	455	(17.8%)	477	1.69	0.85-3.36
3 - 4	29	(16.7%)	449	(17.6%)	478	2.13	1.11-4.09
5 - 6	33	(19.0%)	458	(17.9%)	491	2.43	1.28-4.61
7	76	(43.7%)	687	(26.9%)	763	3.56	1.98-6.38
Risk perception for HIV infection							
Own high-risk behaviour	39	(22.4%)	41	(1.6%)	80	18.82	11.49-30.81
Partner(s)' high-risk behaviour	18	(10.3%)	166	(6.5%)	184	2.18	1.28-3.73
Other reasons	21	(12.1%)	138	(5.4%)	159	3.39	2.05-5.63
None	96	(55.2%)	2210	(86.5%)	2306	1.00	-
STD symptoms in last 12 months							
Yes	29	(16.7%)	215	(8.4%)	244	2.05	1.34-3.13
No	145	(83.3%)	2340	(91.6%)	2485	1	-
Sickness in last 12 months							
HIV-related illness	23	(13.2%)	92	(3.6%)	115	4.09	2.41-6.93
Other illness	81	(46.6%)	1125	(44.0%)	1206	1.37	0.98-1.91
None	69	(39.7%)	1335	(52.3%)	1404	1	-
Psychological distress							
Yes	43	(24.7%)	298	(11.7%)	341	1.31	1.60-3.34

For peer review only

No	131	(75.3%)	2257	(88.3%)	2388	1	-
Pregnancies in last 3 years							
One or more	49	(28.2%)	1077	(42.2%)	1126	0.59	0.40-0.87
None	125	(71.8%)	1478	(57.8%)	1603	2	-
Stigma and discrimination (affecting testing)							
Yes	2	(1.1%)	26	(1.0%)	28	0.05	0.25-4.49
No	172	(98.9%)	2529	(99.0%)	2701	1	-
Travel time to HIV testing facility							
<30 mins	61	(35.1%)	390	(15.3%)	451	1	-
30-59 mins	39	(22.4%)	585	(22.9%)	624	0.42	0.27-0.64
60-89 mins	23	(13.2%)	587	(23.0%)	610	0.24	0.15-0.40
90 mins	48	(27.6%)	849	(33.2%)	897	0.35	0.23-0.52
Uncertain	3	(1.7%)	144	(5.6%)	147	-	-
**Antiretroviral treatment**							
Knowledge of ART							
Yes	126	(72.4%)	1341	(52.5%)	1467	1.35	1.67-3.31
No	48	(27.6%)	1203	(47.1%)	1251	1	-
Stigma and discrimination (in the community)							
Yes	43	(24.7%)	462	(18.1%)	505	0.46	1.01-2.08
No	131	(75.3%)	2090	(81.8%)	2221	1	-
Peer influence							
Relative(s) on ART	41	(23.6%)	469	(18.4%)	510	2.14	1.41-3.24
Friend(s) on ART	55	(31.6%)	333	(13.0%)	388	3.98	2.69-5.89
None	58	(33.3%)	1496	(58.6%)	1554	1	-
Travel time to ART service *							
<30 mins	34	(19.5%)	172	(6.7%)	206	1	-
30-59 mins	17	(9.8%)	261	(10.2%)	278	0.31	0.17-0.57
60-89 mins	17	(9.8%)	224	(8.8%)	241	0.38	0.20-0.70
90 mins	33	(19.0%)	341	(13.3%)	374	0.46	0.28-0.78
Uncertain	73	(42.0%)	1557	(60.9%)	1630	-	-

AOR- age-adjusted odds ratios; 95% CI- 95% confidence intervals
* Includes women not aware of HIV testing and ART services to prevent exclusion of these participants from the multi-variable analysis. Odds ratios were not interpreted for this group as they are not comparable with the reference category

For peer review only

Table 2: Factors contributing to differences in uptake of HIV testing ever in lifetime between FSW and NSW, Manicaland, 2009-2011

		Bivariate Analysis				Socio-demographic		Socio-demographic + Sexwork		Intermediate Determinants		Intermediate Determinants + Sexwork		Full Model	
		n	%	AOR	95% CI	AOR	95% CI	AOR	95% CI	AOR	95% CI	AOR	95% CI	AOR	95% CI
**Female Sex Work**															
Sex Work	NSW	1925	(93.1%)	1	-	-	-	1	-	-	-	1	-	1	-
	FSW	142	(6.9%)	1.5	1.00-2.24	-	-	1.75	1.14-2.69	-	-	0.99	0.63-1.57	1.11	0.69-1.81
**Socio-demographic**															
Age-group															
	19-29	822	(39.8%)	1	-	1	-	1	-	-	-	-	-	1	-
	30-39	622	(30.1%)	0.75	0.59-0.95	0.69	0.53-0.90	0.67	0.51-0.87	-	-	-	-	0.65	0.48-0.89
	40-49	402	(19.4%)	0.46	0.36-0.59	0.46	0.34-0.61	0.45	0.33-0.60	-	-	-	-	0.54	0.37-0.78
	50-58	221	(10.7%)	0.27	0.20-0.35	0.29	0.21-0.41	0.29	0.21-0.41	-	-	-	-	0.39	0.26-0.59
Marital status															
	Never Married	283	(13.7%)	0.28	0.17-0.45	0.31	0.18-0.50	0.3	0.18-0.49	-	-	-	-	0.43	0.24-0.78
	Married	42	(2.0%)	1	-	1	-	1	-	-	-	-	-	1	-
	Divorced or separated	1551	(75.0%)	0.78	0.58-1.05	0.8	0.59-1.09	0.77	0.56-1.05	-	-	-	-	0.83	0.58-1.17
	Widowed	191	(9.2%)	1.3	0.99-1.70	1.4	1.06-1.85	1.38	1.04-1.82	-	-	-	-	1.26	0.92-1.72
Church Denomination															
	Christian	1131	(54.7%)	1	-	1	-	1	-	-	-	-	-	1	-
	Spiritual	701	(33.9%)	0.84	0.69-1.03	0.89	0.72-1.09	0.89	0.73-1.10	-	-	-	-	0.95	0.75-1.20
	Other	203	(9.8%)	0.78	0.58-1.06	0.87	0.63-1.20	0.87	0.63-1.20	-	-	-	-	0.94	0.65-1.34
	None	30	(1.5%)	0.56	0.28-1.09	0.56	0.28-1.13	0.52	0.26-1.06	-	-	-	-	0.43	0.20-0.93
Socio-economic status															
	First (poorest) tercile	1308	(63.3%)	1	-	1	-	1	-	-	-	-	-	1	-
	Second tercile	472	(22.8%)	1.13	0.90-1.41	1.04	0.83-1.30	1.04	0.83-1.31	-	-	-	-	0.94	0.73-1.22
	Third tercile	219	(10.6%)	1.35	0.97-1.87	1.06	0.74-1.53	1.1	0.77-1.59	-	-	-	-	0.9	0.60-1.34
Residential area															
	Town	542	(26.2%)	1	-	1	-	1	-	-	-	-	-	1	-
	Agricultural estate	460	(22.3%)	0.57	0.44-0.74	0.59	0.44-0.79	0.61	0.46-0.82	-	-	-	-	0.63	0.45-0.88
	Roadside settlement	569	(27.5%)	0.81	0.62-1.06	0.79	0.59-1.05	0.81	0.60-1.08	-	-	-	-	0.94	0.68-1.30
	Subsistence farming village	496	(24.0%)	0.71	0.54-0.92	0.7	0.52-0.95	0.73	0.55-0.99	-	-	-	-	0.93	0.67-1.31
Education															
	Primary or less	675	(32.7%)	1	-	1	-	1	-	-	-	-	-	1	-
	Secondary or higher	1392	(67.3%)	1.47	1.18-1.83	1.45	1.15-1.82	1.46	1.16-1.83	-	-	-	-	1.06	0.81-1.37
Children alive															
	None	257	(12.4%)	1	-	1	-	1	-	-	-	-	-	1	-
	1	632	(30.6%)	1.9	1.45-2.50	1.63	1.23-2.16	1.67	1.26-2.22	-	-	-	-	1.52	1.10-2.09
	2	586	(28.4%)	1.79	1.35-2.36	1.53	1.14-2.05	1.57	1.17-2.11	-	-	-	-	1.48	1.06-2.06
	3	343	(16.6%)	1.6	1.18-2.18	1.43	1.04-1.98	1.5	1.08-2.07	-	-	-	-	1.45	1.01-2.08
	4	249	(12.0%)	1.88	1.33-2.65	1.73	1.21-2.48	1.79	1.24-2.57	-	-	-	-	1.72	1.14-2.59
**Intermediate Determinants**															
HIV Result															
	Positive	454	(22.0%)	1.09	0.88-1.36	-	-	-	-	-	-	-	-	-	-

For peer review only

Negative	1611	(77.9%)	1	-	-	-	-	-	-	-	-	-	-	-
Knowledge about HIV risks														
Good	1787	(86.5%)	1.5	1.18-1.90	-	-	-	-	1.42	1.08-1.86	1.42	1.08-1.86	1.35	1.02-1.80
Poor	280	(13.5%)	1	-	-	-	-	-	1	-	1	-	1	-
Risk perception for HIV infection														
Own high-risk behaviour	68	(3.3%)	2.36	1.26-4.43	-	-	-	-	1.21	0.62-2.36	1.21	0.60-2.43	1.3	0.63-2.70
Partner(s)' high-risk behaviour	172	(8.3%)	6.21	3.41-11.29	-	-	-	-	4.53	2.30-8.94	4.53	2.30-8.94	4.61	2.29-9.29
Other reasons	133	(6.4%)	2.12	1.37-3.29	-	-	-	-	1.47	0.92-2.35	1.47	0.92-2.35	1.41	0.87-2.29
None	1694	(82.0%)	1	-	-	-	-	-	1	-	1	-	1	-
Knowing persons living with or who PLHIV / died from HIV														
0	344	(16.6%)	1	-	-	-	-	-	1	-	1	-	1	-
1 - 2	350	(16.9%)	1.51	1.14-2.00	-	-	-	-	1.14	0.83-1.57	1.14	0.83-1.57	1.12	0.80-1.57
3 - 4	392	(19.0%)	2.56	1.89-3.47	-	-	-	-	2.09	1.48-2.95	2.09	1.48-2.95	2.04	1.43-2.91
5 - 6	389	(18.8%)	2.26	1.69-3.04	-	-	-	-	1.53	1.11-2.12	1.53	1.11-2.12	1.47	1.05-2.06
7	592	(28.6%)	2.07	1.60-2.69	-	-	-	-	1.35	1.01-1.82	1.35	1.01-1.82	1.27	0.93-1.72
STD symptoms in last 12 months														
Yes	195	(9.4%)	1.5	1.00-2.24	-	-	-	-	0.83	0.57-1.20	0.83	0.57-1.20	0.83	0.56-1.22
No	1872	(90.6%)	1	-	-	-	-	-	1	-	1	-	1	-
Sickness in last 12 months														
HIV-related illness	109	(5.3%)	8.08	3.50-18.67	-	-	-	-	3.78	1.55-9.18	3.78	1.55-9.18	4.35	1.76-10.71
Other illness	904	(43.7%)	1.04	0.87-1.25	-	-	-	-	0.97	0.80-1.19	0.97	0.80-1.19	0.97	0.78-1.20
None	1052	(50.9%)	1	-	-	-	-	-	1	-	1	-	1	-
Psychological distress														
Yes	259	(12.5%)	1.12	0.85-1.47	-	-	-	-	-	-	-	-	-	-
No	1808	(87.5%)	1	-	-	-	-	-	-	-	-	-	-	-
Pregnancies in last 3 years														
One or more	974	(47.1%)	2.32	1.82-2.96	-	-	-	-	2.51	1.91-3.28	2.5	1.91-3.28	2.42	1.82-3.22
None	1093	(52.9%)	1	-	-	-	-	-	1	-	1.00	-	1	-
Travel time to HIV testing facility*														
<30 mins	385	(18.6%)	1	-	-	-	-	-	1	-	1	-	1	-
30 - 59 mins	508	(24.6%)	0.78	0.56-1.09	-	-	-	-	0.73	0.51-1.03	0.73	0.51-1.03	0.73	0.51-1.06
60 - 89 mins	470	(22.7%)	0.63	0.45-0.87	-	-	-	-	0.58	0.41-0.82	0.58	0.41-0.82	0.5	0.35-0.73
90 mins	690	(33.4%)	0.63	0.46-0.86	-	-	-	-	0.58	0.42-0.80	0.58	0.42-0.80	0.47	0.33-0.67
Uncertain	14	(.7%)	-	-	-	-	-	-	-	-	-	-	-	-
Knowledge of ART														
Yes	1224	(59.2%)	2.38	1.98-2.86	-	-	-	-	1.51	1.23-1.87	1.51	1.23-1.87	1.48	1.19-1.85
No	833	(40.3%)	1	-	-	-	-	-	1	-	1	-	1	-
Stigma and discrimination (affecting testing)														
Yes	19	(.9%)	0.76	0.34-1.73	-	-	-	-	-	-	-	-	-	-
No	2048	(99.1%)	1	-	-	-	-	-	-	-	-	-	-	-
Stigma and discrimination (in the community)														
Yes	395	(19.1%)	1.18	0.93-1.50	-	-	-	-	-	-	-	-	-	-
No	1669	(80.7%)	1	-	-	-	-	-	-	-	-	-	-	-

AOR- age-adjusted odds ratios; 95% CI- 95% confidence intervals
* Includes women not aware of HIV testing and ART services to prevent exclusion of these participants from the multi-variable analysis. Odds ratios were not interpreted for this group as they are not comparable with the reference category

For peer review only
For peer review only - http://bmjopen.bmj.com/site/about/guidelines.xhtml

Table 3: Factors contributing to differences in uptake of antiretroviral treatment between FSW and NSW, Manicaland, Zimbabwe, 2009-2011

		Bivariate Analysis				Socio-demographic		Socio-demographic Sexwork		Intermediate Determinants		Intermediate Determinants Sexwork		Full Model	
		n	%	AOR	95% CI	AOR	95% CI	AOR	95% CI	AOR	95% CI	AOR	95% CI	AOR	95% CI
**Female Sex Work**															
**Sex Work**	NSW	138	(75.4%)	1	-	-	-	1	-	-	-	1	-	1	-
	FSW	45	(24.6%)	2.64	1.16-6.00	-	-	2.28	0.97-5.39	-	-	3.46	0.91-13.16	3.51	0.79-15.47
**Socio-demographic**															
**Age**															
	Age (continuous)	-	-	1.53	1.14-2.06	1.62	1.19-2.22	1.57	1.14-2.15	-	-	-	-	1.63	1.03-2.57
	Age2	-	-	1	0.99-1.00	0.99	0.99-1.00	1	0.99-1.00	-	-	-	-	1	0.99-1.00
**Marital status**															
	Never Married	2	(1.1%)	0.33	0.04-2.57	-	-	-	-	-	-	-	-	-	-
	Married	72	(39.3%)	1	-	-	-	-	-	-	-	-	-	-	-
	Divorced/Separated	29	(15.8%)	1.56	0.66-3.65	-	-	-	-	-	-	-	-	-	-
	Widowed	80	(43.7%)	1.41	0.73-2.74	-	-	-	-	-	-	-	-	-	-
**Church Denomination**															
	Christian churches	100	(54.6%)	1	-	-	-	-	-	-	-	-	-	-	-
	Spiritual churches	59	(32.2%)	0.62	0.35-1.12	-	-	-	-	-	-	-	-	-	-
	Other religion	19	(10.4%)	1.29	0.46-3.58	-	-	-	-	-	-	-	-	-	-
	No religion	5	(2.7%)	2	0.30-13.48	-	-	-	-	-	-	-	-	-	-
**Socio-economic status**															
	1 (poorest)	113	(61.7%)	1	-	-	-	-	-	-	-	-	-	-	-
	2	47	(25.7%)	1.21	0.63-2.34	-	-	-	-	-	-	-	-	-	-
	3	20	(10.9%)	2.3	0.82-6.44	-	-	-	-	-	-	-	-	-	-
**Residential area**															
	Town	62	(33.9%)	1	-	1	-	1	-	-	-	-	-	1	-
	Agricultural estate	54	(29.5%)	1.1	0.52-2.35	1.27	0.58-2.76	1.37	0.62-3.01	-	-	-	-	2.53	0.77-8.29
	Roadside settlement	35	(19.1%)	0.48	0.23-1.01	0.54	0.26-1.15	0.6	0.28-1.28	-	-	-	-	0.87	0.29-2.61
	Subsistence farming village	32	(17.5%)	0.82	0.37-1.82	0.96	0.42-2.16	1.01	0.44-2.30	-	-	-	-	1.78	0.53-5.95
**Education**															
	Primary or less	86	(47.0%)	1	-	-	-	-	-	-	-	-	-	-	-
	Secondary or higher	97	(53.0%)	1.09	0.58-2.07	-	-	-	-	-	-	-	-	-	-
**Children alive**															
	0	50	(27.3%)	1	-	1.00	-	1	-	-	-	-	-	1	-
	1	52	(28.4%)	0.44	0.18-1.08	0.44	0.18-1.10	0.49	0.19-1.24	-	-	-	-	0.2	0.04-1.12
	2	40	(21.9%)	0.37	0.15-0.95	0.39	0.15-1.02	0.46	0.18-1.22	-	-	-	-	0.13	0.02-0.76
	3	24	(13.1%)	0.27	0.10-0.75	0.27	0.10-0.74	0.31	0.11-0.87	-	-	-	-	0.13	0.02-0.80
	4	17	(9.3%)	0.24	0.08-0.72	0.24	0.08-0.75	0.25	0.08-0.78	-	-	-	-	0.05	0.01-0.36
**Intermediate Determinants**															
**Knowledge about HIV risks**															
	Good	162	(88.5%)	1.18	0.53-2.63	-	-	-	-	-	-	-	-	-	-

For peer review only

	Poor	21	(11.5%)	1	-	-	-	-	-	-	-	-	-	-	-
**Risk perception for HIV infection**															
	Own high-risk behaviour	37	(20.2%)	1.1	0.38-3.16	-	-	-	-	-	-	-	-	-	-
	Partner(s)' high-risk behaviour	92	(50.3%)	1.06	0.42-2.68	-	-	-	-	-	-	-	-	-	-
	Other reasons	36	(19.7%)	0.81	0.29-2.26	-	-	-	-	-	-	-	-	-	-
	None	18	(9.8%)	1	-	-	-	-	-	-	-	-	-	-	-
**Peer influence**															
	Relative(s) on ART	72	(39.3%)	9.12	4.25-19.58	-	-	-	-	1.95	0.70-5.42	1.83	0.66-5.06	1.79	0.61-5.20
	Friend(s) on ART	91	(49.7%)	16.38	7.22-37.20	-	-	-	-	3.04	1.03-8.93	2.52	0.85-7.46	2.19	0.69-6.97
	None	20	(10.9%)	1	-	-	-	-	-	1	-	1	-	1	-
**Sickness in last 12 months**															
	HIV-related illness	69	(37.7%)	1.09	0.57-2.09	-	-	-	-	-	-	-	-	-	-
	Other illness	42	(23.0%)	0.69	0.35-1.35	-	-	-	-	-	-	-	-	-	-
	None	72	(39.3%)	1	-	-	-	-	-	-	-	-	-	-	-
**STD symptoms in last 12 months**															
	Yes	51	(27.9%)	1.03	0.56-1.89	-	-	-	-	-	-	-	-	-	-
	No	132	(72.1%)	1	-	-	-	-	-	-	-	-	-	-	-
**Psychological distress**															
	Yes	36	(19.7%)	0.46	0.24-0.86	-	-	-	-	0.43	0.19-0.99	0.41	0.18-0.96	0.48	0.20-1.18
	No	147	(80.3%)	1	-	-	-	-	-	1	-	1	-	1	-
**Pregnancies in last 3 years**															
	One or more	28	(15.3%)	0.86	0.41-1.80	-	-	-	-	-	-	-	-	-	-
	None	155	(84.7%)	1	-	-	-	-	-	-	-	-	-	-	-
**Travel time to ART service***															
	<30 mins	43	(23.5%)	1	-	-	-	-	-	1	-	1	-	1	-
	30 - 59 mins	42	(23.0%)	0.27	0.08-0.86	-	-	-	-	0.27	0.08-0.89	0.34	0.10-1.15	0.41	0.11-1.58
	60 - 89 mins	34	(18.6%)	0.32	0.09-1.18	-	-	-	-	0.37	0.10-1.40	0.42	0.11-1.62	0.34	0.07-1.67
	90 mins	63	(34.4%)	0.37	0.12-1.16	-	-	-	-	0.46	0.14-1.46	0.51	0.15-1.65	0.65	0.17-2.49
	Uncertain	1	(.5%)	-	-	-	-	-	-	-	-	-	-	-	-
**Stigma and discrimination (in the community)**															
	Yes	38	(20.8%)	1.3	0.64-2.64	-	-	-	-	-	-	-	-	-	-
	No	145	(79.2%)	1	-	-	-	-	-	-	-	-	-	-	-

AOR- age-adjusted odds ratios; 95% CI- 95% confidence intervals

* Includes women not aware of HIV testing and ART services to prevent exclusion of these participants from the multi-variable analysis. Odds ratios were not interpreted for this group as they are not comparable with the reference category

For peer review only

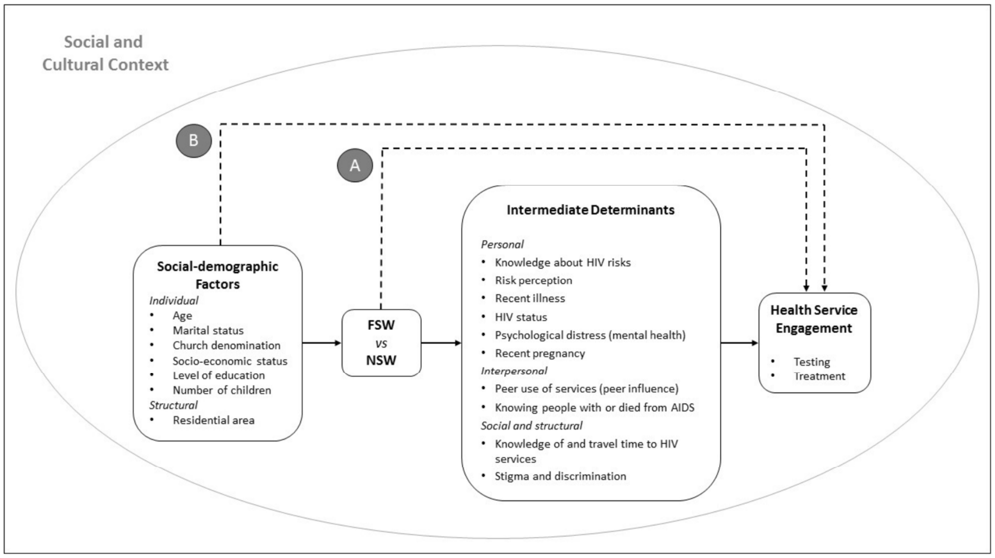

Figure 1: Theoretical framework illustrating how engagement in sex work (or not) may influence use of HIV testing and treatment services (dashed line A). This framework hypothesises that individuals' uptake of services may be influenced by various socio-demographic characteristics (dashed line B), and that these factors may be mediated by involvement in sex work which, in turn, alters uptake of services. Involvement in sex work is not considered to alter uptake of services per se; rather, engagement in sex work is associated with different social, structural and psychosocial experiences compared to non-sex workers which, in turn, may drive differential uptake of services by sex work status.

Fig 2a: Comparison of HIV treatment cascades for female sex workers and non-sex workers in Manicaland, Zimbabwe, 2009-2011.

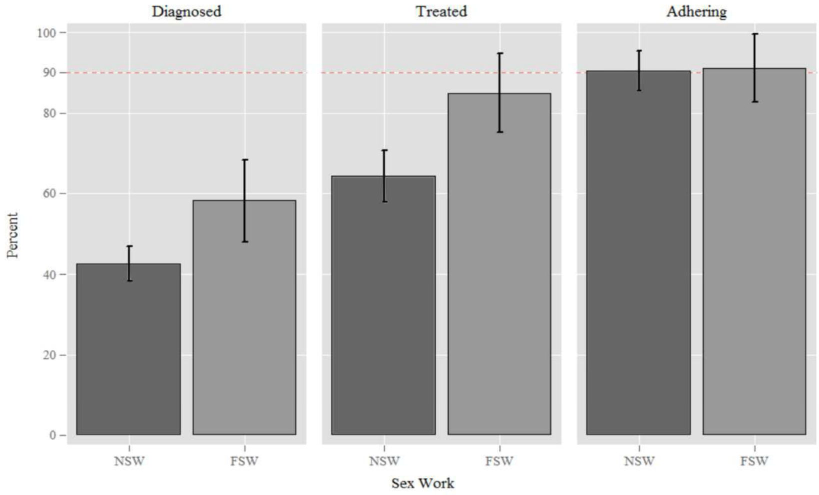

Fig 2b: Comparison of cumulative HIV treatment cascades for female sex workers and non-sex workers in Manicaland, Zimbabwe, 2009-2011.

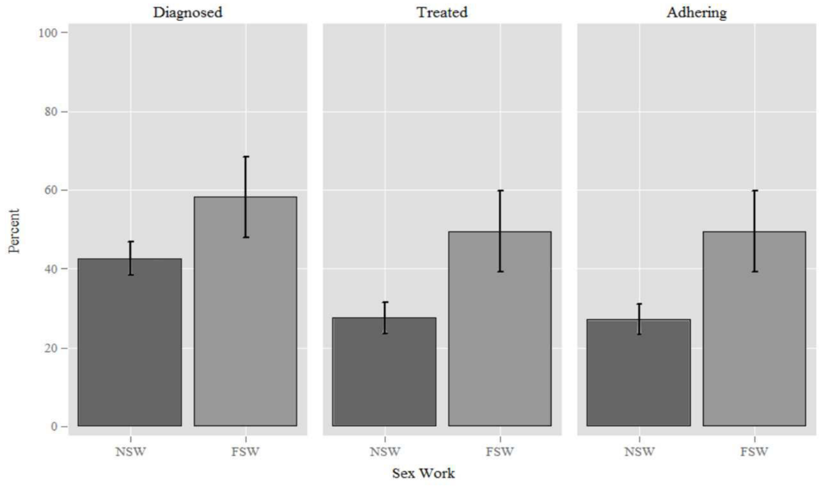

Figure 2a & 2b: Comparisons of HIV treatment cascades. Figures illustrating the proportion of FSW and NSW who achieve optimal outcomes at each stage of the cascade. Fig 2a shows the proportions of HIV-positive women who have been diagnosed, the proportions of treated amongst those who have been diagnosed, and the proportions adhering to their medication amongst those who have been treated. A 90% reference line is included to illustrate UNAIDS targets. For Fig 2b, the denominator is all HIV-positive women at each stage of the cascade.

Supplementary Material

A. Theoretical framework for factors mediating uptake of HIV services among female sex workers vs non-sex workers

Several theoretical models of the mechanisms for behaviour change have been successfully applied to reduce risky sexual behaviour by individuals [1]. However, recognition of the limitations of individual-level approaches to HIV prevention (such as to what extent condom use is solely related to self-efficacy without consideration of gendered power dynamics) has led to a growth in structural models for HIV risk [2]. Multilevel theoretical models draw on the strengths of both individual-level focused models and structural models, but in delineating the links between these levels have great potential power for theory-driven approaches to combination prevention [3]. The Network-Individual-Resource Model (NIRM) for HIV Prevention posits that membership of distinct social networks can attenuate or enhance individual-level factors driving HIV risk [4]. For example, intense stigma and discrimination frequently marginalises female sex workers (FSW) from wider society such that FSW may occupy distinct social networks from non-sex workers (NSW). Therefore, various factors relating these distinct social networks may mean that FSWs' use of healthcare services may be dissimilar to that of NSW. These factors comprise individual-level preferences and behaviours as well as relations with peers, relatives and the community and the legal and socio-cultural context. Drawing on a rich literature of multi-level theoretical approaches to behaviour change and HIV transmission prevention [3–5], we describe a new framework (Figure S1) to explain how intermediate factors at different levels may be associated with HIV service uptake in

For peer review only

For peer review only - http://bmjopen.bmj.com/site/about/guidelines.xhtml

testable relations in a Zimbabwean context, a subset of which are explored in this paper (Figure 1).

Supplementary Figure 1: Generalised theoretical framework for intermediate factors mediating differential uptake of HIV services by female sex workers (FSW) compared to non-sex workers (NSW).

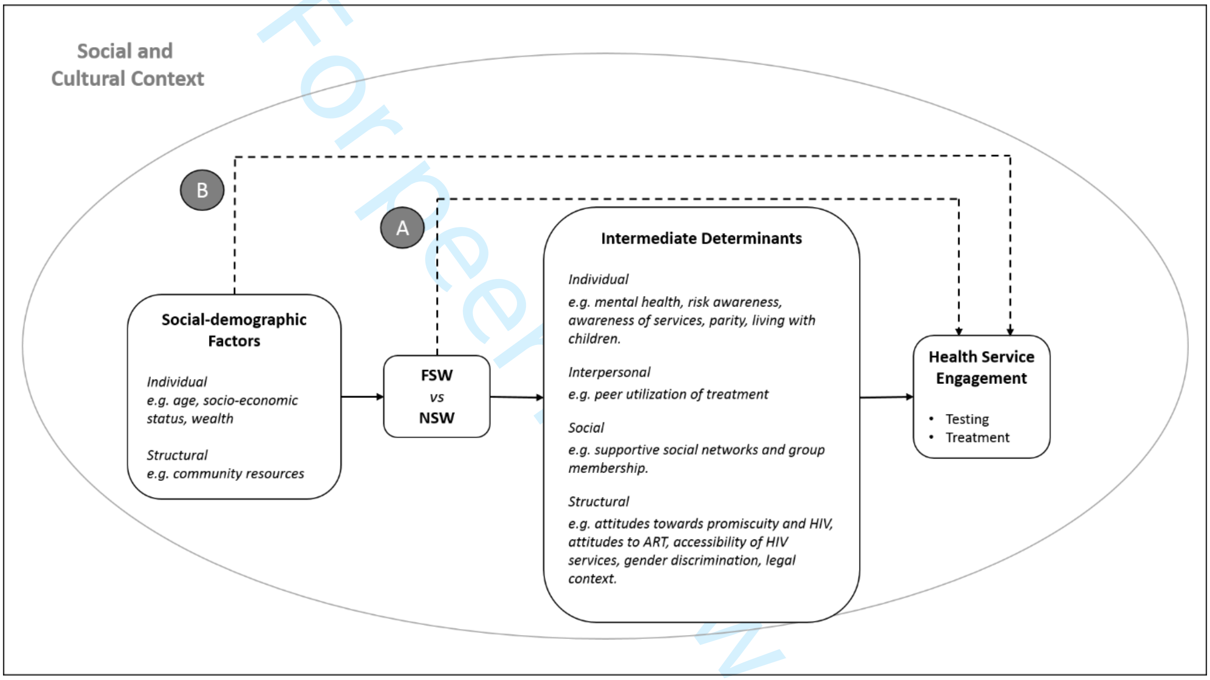

For peer review only

For peer review only - http://bmjopen.bmj.com/site/about/guidelines.xhtml

Influence of structural factors on uptake of health services

Structural factors include social, cultural, economic, legal and political contexts which shape and frame behaviours, actions and norms of communities and agents [2]. In meta-analyses evaluating barriers to retention and linkage to care, distance from testing facilities and costs of transport have been identified as the most important obstacles [6], yet to what extent this is true in FSW as well as NSW is unclear. Since FSW tend to most commonly live in more urban areas than NSW [17] where facilities are most concentrated and more closely available, we might expect distances and costs of transport to be different between FSW and NSW. However, travel also incurs an opportunity cost for FSW who experience loss of earnings during the time taken to travel [7] so this may attenuate their uptake relative to NSW. In addition, FSW often migrate both internally and across national borders [8,9], often away from families even including children. Mobility and migration affect uptake of services in complex ways that are dependent on a variety of contextual factors (e.g. relative availability of services in source and sink destinations, whether migration is internal, circular or international). Migration from high to low prevalence settings is associated with lower HIV risk [10] but migrants are more likely to be unaware of local services which can reduce access. In addition, circular migration can interrupt treatment or cause delays in treatment [11].

Fear of social rejection and discrimination from positive HIV diagnosis often deters individuals from seeking testing [7,12,13]. For FSW, this fear may be more intense because of higher rates of HIV among FSW than NSW (see individual-factors below) and because they already experience intense stigma and discrimination as a result of selling sex. Laws criminalising sex work mean that sex workers are often subject to arrest and violence perpetrated by police [2,14,15]. Such laws often intersect with gendered attitudes towards acceptable behaviour for women, often compounding long-lived taboos around female promiscuity [16,17]. Such stigma

For peer review only - http://bmjopen.bmj.com/site/about/guidelines.xhtml

frequently results in harassment [16] and can cause unnecessary delays to treatment from healthcare workers [18] or deter FSW from accessing care altogether [19].

Influence of social factors on uptake of health services

Evidence for social factors influencing uptake is based on trials of various interventions to encourage treatment initiation and adherence. In the wider community, mobilisation, group membership and empowerment (either informal or formal) have been successful in encouraging HIV testing and treatment through enhancing social capital (networks of inter-group relationships that are socially enhancing) and self-efficacy [20,21]. Similar approaches have been targeted towards mobilising sex worker communities (e.g. by uniting sex workers in a common cause for health improvement, creating spaces for debate of new health information and tackling powerful actors that actively disenfranchise sex workers through violence, stigma or discrimination [22] have demonstrated substantial effectiveness in reducing HIV infection and other STIs and increasing condom use [23]. An important component of community mobilisation is the development and strengthening of social capital and facilitating "transformative social spaces". One approach to this is encouraging participation in community groups. Such groups can have powerful positive impacts on risk behaviours and healthcare seeking, by providing a critical dialogue of harmful social norms, providing emotional and material support and by forming positive action plans and solidarity to mobilise them [21]. Conversely, they can also entrench negative norms and facilitate dissemination of false information. It is unclear how community membership may have differential impact on FSW and NSW in enhancing/attenuating service uptake.

Influence of individual and interpersonal level factors on uptake of health services

For peer review only - http://bmjopen.bmj.com/site/about/guidelines.xhtml

A complex interplay of biological and behavioural factors drive differences in HIV risk in FSW compared to NSW which in turn will influence their respective need for and exposure to HIV services. Unsurprisingly, awareness and knowledge of HIV services have been identified as a critical component to encouraging service uptake. Batona et al found FSW who had previously undergone HIV counselling and testing (HTC) were more likely to become engaged with services a second time and displayed less resistance to testing and initiation in the treatment cascade [24]. A synergistic and reciprocal relationship exists between STIs (such as HSV-2 and bacterial vaginosis) and HIV such that acquisition of one can facilitate acquisition and transmission of the other [25–27]. Unprotected sex with multiple sexual partners puts FSWs at greater risk than NSW of symptomatic STIs and HIV. Consequently, FSW may be more likely to access services than NSW to resolve these health concerns, not least because ill-health may cause loss of earnings. Relatedly, greater perceived risk among FSWs may drive higher rates of health service uptake [1,8].

For many women worldwide, initial exposure to HIV testing is via antenatal care services (ANC). We might expect lower exposure to HIV testing through ANC for FSW for a couple of reasons. First, since FSW have higher prevalence of HIV than NSW and HIV reduces fertility [12] , we might expect incidence of pregnancy among FSW to be lower. Second, pregnancy represents an opportunity cost for FSW (loss of earnings) and so they may be more likely to take steps to avoid it (e.g. hormonal contraception).

A systematic reivew of barriers and faciliators to accessing ART care globally found a number of individual-level barriers were influential including younger age, lower education level, longer distances from clinics, higher transport costs, as well as inability to take time off work and other time constraints [6]. In a previous study of sex workers in Zimbabwe, we found FSW

For peer review only - http://bmjopen.bmj.com/site/about/guidelines.xhtml

were significantly higher educated, older and were more likely to live in urban areas where facilities are more closely available. Differences in wealth of FSW compared to NSW may mean they have different capacities to pay for healthcare-related costs [28]. Conversely, if sex workers are less likely to live with children (either because they have no children or have travelled to work), FSW may have lower childcare related expenditures than NSW which may mean greater disposable income for healthcare-related expenditures [29].

High rates of mental health disorders in FSW have been attributed to discrimination and social rejection as a result of their work, higher rates of violence (physical, sexual and emotional) from clients, non-paying intimate partners, police and economic pressures to support dependents [5]. Data from Zimbabwe suggest FSW have higher levels of mental ill-health than NSW and that mental ill-health is linked to poorer adherence to ART [30]. In addition to the fear of HIV positive diagnosis, disclosure as HIV positive connotes additional negative consequences for FSW, it being undesirable for potential clients and potentially resulting in a loss of earnings.

In frameworks for HIV risk, interpersonal factors include frequency and type of sexual relationships and the negotiation of condom use therein [2,5]. Intimate male partners can effectively control their female spouse's access to HIV treatment, causing substantial treatment delays [6,13] intimate partner violence has been linked to lower ART use and viral load suppression [31] and for FSW having an intimate partnership can present a significant obstacle to achieving viral suppression [32]. If NSW are more likely to have an intimate male partners than FSW, we might therefore expect uptake among NSW to be more affected by the influence of partners.

For peer review only - http://bmjopen.bmj.com/site/about/guidelines.xhtml

The impact of interpersonal factors on health-service uptake need not relate solely to sexual relationships but may also be driven through social relationships. In HIV prevention, use of peers has had important beneficial impact in enhancing knowledge of HIV risks, encouraging condom use and reducing HIV/STI infections [33,34]. Use of peers to encourage uptake of HIV care is less well studied. In India, a requirement to take a "buddy" or family member before treatment was issued prevented FSW and MSM from accessing services [7] and peer-led interventions may be limited if the social environment is not health-enabling [35]. Nevertheless, peers have been used with some success in preventing mother-to-child transmission of HIV [36] and near-peers (health workers with shared cultural background with clients) have been used in the US to significantly increase viral load suppression by helping patients navigate non-integrated HIV care systems [37]. We hypothesis peer use of HIV care as a potential factor to encourage service access.

B. Shona Symptom Questionnaire

Mental health was assessed using the Shona Symptom Questionnaire (SSQ), a 14-item questionnaire of 'yes or 'no' questions, developed and validated in Zimbabwe in 1997 with the aid of mental healthcare providers [38]. The SSQ quantifies psychological distress as a function of somatic and psychological experiences over the week prior to interview. Using validated cut-points indicating levels of psychological distress [38], a dichotomous variable (0/1) was created with individuals with an SSQ score ≥7 (coded 1) as currently experiencing psychological distress [30].

For peer review only

C. HIV testing amongst HIV+ women

Table S1: Factors contributing to the difference in uptake of HIV testing between HIV-positive FSW and NSW, Manicaland Zimbabwe, 2009-2011

				Bivariate		Socio-demographic		Socio-demographic Sexwork		Intermediate Determinants		Intermediate Determinants Sexwork		Full Model	
				AOR	95% CI	AOR	95% CI	AOR	95% CI	AOR	95% CI	AOR	95% CI	AOR	95% CI
**Female Sex Work**															
Sex Work	NSW	379	(83.48%)	1	-	-	-	1	-	-	-	1	-	1	-
	FSW	75	(16.52%)	1.51	0.85-2.70	-	-	1.83	1.00-3.37	-	-	1.02	0.51,2.05	1.14	0.56,2.35
**Socio-demographic**															
Age-group															
	19-29	96	(21.15%)	1	-	-	-	-	-	-	-	-	-	-	-
	30-39	178	(39.21%)	1.06	0.64-1.75	-	-	-	-	-	-	-	-	-	-
	40-49	126	(27.75%)	1.31	0.75-2.29	-	-	-	-	-	-	-	-	-	-
	50-58	54	(11.89%)	0.82	0.43-1.55	-	-	-	-	-	-	-	-	-	-
Marital status															
	Never married	139	(30.62%)	0.52	0.18-1.52	-	-	-	-	-	-	-	-	-	-
	Married	9	(1.98%)	1	-	-	-	-	-	-	-	-	-	-	-
	Divorced or separated	237	(52.2%)	0.90	0.53-1.54	-	-	-	-	-	-	-	-	-	-
	Widowed	69	(15.2%)	1.45	0.89-2.38	-	-	-	-	-	-	-	-	-	-
Religion															
	Christian	238	(52.42%)	1	-	1	-	1	-	-	-	-	-	1	-
	Spiritual	156	(34.36%)	0.93	0.60-1.42	1.00	0.65-1.55	1.02	0.66-1.58	-	-	-	-	1.09	0.64,1.85
	Other	48	(10.57%)	0.69	0.38-1.24	0.77	0.42-1.42	0.76	0.41-1.39	-	-	-	-	0.79	0.38,1.63
	None	12	(2.64%)	0.42	0.16-1.08	0.50	0.19-1.33	0.41	0.15-1.12	-	-	-	-	0.33+	0.10,1.07

For peer review only

For peer review only - http://bmjopen.bmj.com/site/about/guidelines.xhtml

Socio-economic status															
	First (poorest) tercile	284	(62.56%)	1	-	-	-	-	-	-	-	-	-	-	-
	Second tercile	114	(25.11%)	1.31	0.82-2.10	-	-	-	-	-	-	-	-	-	-
	Third tercile	48	(10.57%)	1.08	0.58-2.02	-	-	-	-	-	-	-	-	-	-
Residential area															
	Town	143	(31.5%)	1	-	1	-	1	-	-	-	-	-	1	-
	Agricultural estate	125	(27.53%)	1.02	0.64-1.64	1.02	0.63-1.65	1.06	0.65-1.73	-	-	-	-	1.53	0.84,2.79
	Roadside settlement	79	(17.4%)	1.61	0.93-2.79	1.46	0.83-2.54	1.52	0.87-2.67	-	-	-	-	2.25*	1.14,4.42
	Subsistence farming village	107	(23.57%)	1.20	0.69-2.10	1.09	0.61-1.93	1.13	0.64-2.01	-	-	-	-	1.47	0.70,3.10
Education															
	Primary or none	178	(39.21%)	1	-	1	-	1	-	-	-	-	-	1	-
	Secondary or higher	276	(60.79%)	1.75	1.14-2.71	1.63	1.04-2.53	1.66	1.06-2.58	-	-	-	-	1.47	0.85,2.55
Children alive															
	None	102	(22.47%)	1	-	-	-	-	-	-	-	-	-	-	-
	1	140	(30.84%)	1.15	0.68-1.96	-	-	-	-	-	-	-	-	-	-
	2	106	(23.35%)	0.91	0.53-1.57	-	-	-	-	-	-	-	-	-	-
	3	60	(13.22%)	0.95	0.50-1.82	-	-	-	-	-	-	-	-	-	-
	4+	46	(10.13%)	1.65	0.73-3.73	-	-	-	-	-	-	-	-	-	-
**Intermediate Determinants**															
Knowledge about HIV risks															
	Good	395	(87.%)	1.28	0.75,2.18	-	-	-	-	-	-	-	-	-	-
	Poor	59	(13.%)	1	-	-	-	-	-	-	-	-	-	-	-
Risk perception for HIV infection															
	Own high-risk behaviour	55	(12.11%)	4.53***	2.06,9.93	-	-	-	-	2.49*	1.06,5.84	2.47*	1.01,6.02	3.13*	1.22,8.08
	Partner(s)' high-risk behaviour	136	(29.96%)	22.46***	8.05,62.68	-	-	-	-	16.57***	5.28,51.93	16.58***	5.29,51.98	18.51***	5.77,59.35
	Other reasons	66	(14.54%)	4.78***	2.29,10.01	-	-	-	-	3.22**	1.47,7.07	3.22**	1.46,7.07	3.57**	1.59,7.99

For peer review only

For peer review only - http://bmjopen.bmj.com/site/about/guidelines.xhtml

	None	197	(43.39%)	1	-	-	-	-	-	1	-	1	-	1	-
Knowing PLHIV / died from HIV															
	0	53	(11.67%)	1	-	-	-	-	-	1	-	1	-	1	-
	1 - 2	85	(18.72%)	2.23**	1.21,4.09	-	-	-	-	1.70	0.82,3.52	1.7	0.82,3.52	1.78	0.84,3.75
	3 - 4	74	(16.3%)	2.69**	1.39,5.22	-	-	-	-	1.79	0.82,3.88	1.79	0.82,3.88	1.73	0.79,3.82
	5 - 6	85	(18.72%)	3.15***	1.63,6.08	-	-	-	-	2.01+	0.93,4.34	2.01+	0.93,4.34	2.17+	0.98,4.80
	7	157	(34.58%)	2.72***	1.56,4.73	-	-	-	-	1.18	0.60,2.33	1.18	0.60,2.33	1.18	0.59,2.37
STD symptoms in last 12 months															
	Yes	86	(18.94%)	1.80*	1.02,3.20	-	-	-	-	0.63	0.31,1.25	0.63	0.31,1.26	0.67	0.33,1.36
	No	368	(81.06%)	1	-	-	-	-	-	1	-	1	-	1	-
Sickness in last 12 months															
	HIV-related illness	95	(20.93%)	0.06***	0.02,0.21	-	-	-	-	0.14**	0.04,0.49	0.14**	0.04,0.49	0.12**	0.03,0.45
	Other illness	165	(36.34%)	0.10***	0.03,0.33	-	-	-	-	0.22*	0.06,0.78	0.22*	0.06,0.79	0.20*	0.06,0.74
	None	193	(42.51%)	1	-	-	-	-	-	1	-	1	-	1	-
Psychological distress															
	Yes	366	(80.62%)	1.23	0.74,2.05	-	-	-	-	-	-	-	-	-	-
	No	88	(19.38%)	1	-	-	-	-	-	-	-	-	-	-	-
Pregnancies in last 3 years															
	One or more	335	(73.79%)	1.29	0.78,2.13	-	-	-	-	-	-	-	-	-	-
	None	119	(26.21%)	1	-	-	-	-	-	-	-	-	-	-	-
Travel time to HIV testing facility															
	<30 mins	106	(23.35%)	1	1.00,1.00	-	-	-	-	1	-	1	-	1	-
	30 - 59 mins	113	(24.89%)	0.64	0.35,1.16	-	-	-	-	0.58	0.29,1.12	0.58	0.29,1.13	0.50+	0.25,1.01
	60 - 89 mins	91	(20.04%)	0.57	0.30,1.05	-	-	-	-	0.53+	0.26,1.06	0.53+	0.26,1.06	0.39*	0.18,0.84
	90 mins	142	(31.28%)	0.9	0.49,1.66	-	-	-	-	0.81	0.41,1.61	0.82	0.41,1.62	0.65	0.31,1.33
	Uncertain	2	(.44%)	0.02***	0.00,0.08	-	-	-	-	0.03***	0.01,0.13	0.03***	0.01,0.13	0.02***	0.00,0.10
Knowledge of ART															
	Yes	345	(75.99%)	3.47***	2.33,5.16	-	-	-	-	1.21	0.74,1.98	1.21	0.74,1.98	1.07	0.63,1.82

For peer review only

	No	108	(23.79%)	1	-	-	-	-	-	1	-	1	-	1	-
Stigma and discrimination (in the community)															
	Yes	90	(19.82%)	1.27	0.77,2.11	-	-	-	-	-	-	-	-	-	-
	No	363	(79.96%)	1	-	-	-	-	-	-	-	-	-	-	-

For peer review only

For peer review only - http://bmjopen.bmj.com/site/about/guidelines.xhtml

Supplementary References

1. Albarracín D, Gillette JC, Earl AN, Glasman LR, Durantini MR, Ho M-H. A Test of Major Assumptions About Behavior Change: A Comprehensive Look at the Effects of Passive and Active HIV-Prevention Interventions Since the Beginning of the Epidemic. Psychol Bull. 2005;131: 856–897. doi:10.1037/0033-2909.131.6.856

2. Shannon K, Goldenberg SM, Deering KN, Strathdee SA. HIV infection among female sex workers in concentrated and high prevalence epidemics: why a structural determinants framework is needed. Curr Opin HIV AIDS. 2014;9: 174–182. doi:10.1097/COH.0000000000000042

3. Kaufman MR, Cornish F, Zimmerman RS, Johnson BT. Health Behavior Change Models for HIV Prevention and AIDS Care: Practical Recommendations for a Multi-Level Approach. J Acquir Immune Defic Syndr 1999. 2014;66: S250–S258. doi:10.1097/QAI.0000000000000236

4. Johnson BT, Redding CA, DiClemente RJ, Mustanski BS, Dodge BM, Sheeran P, et al. A Network-Individual-Resource Model for HIV Prevention. AIDS Behav. 2010;14: 204–221. doi:10.1007/s10461-010-9803-z

5. Shannon K, Strathdee SA, Goldenberg SM, Duff P, Mwangi P, Rusakova M, et al. Global epidemiology of HIV among female sex workers: influence of structural determinants. The Lancet. 2015;385: 55–71. doi:10.1016/S0140-6736(14)60931-4

6. Govindasamy D, Ford N, Kranzer K. Risk factors, barriers and facilitators for linkage to antiretroviral therapy care: a systematic review. AIDS Lond Engl. 2012;26: 2059–2067. doi:10.1097/QAD.0b013e3283578b9b

7. Beattie TSH, Bhattacharjee P, Suresh M, Isac S, Ramesh BM, Moses S. Personal, interpersonal and structural challenges to accessing HIV testing, treatment and care services among female sex workers, men who have sex with men and transgenders in Karnataka state, South India. J Epidemiol Community Health. 2012;66 Suppl 2: ii42-48. doi:10.1136/jech-2011-200475

8. Desmond N, Allen CF, Clift S, Justine B, Mzugu J, Plummer ML, et al. A typology of groups at risk of HIV/STI in a gold mining town in north-western Tanzania. Soc Sci Med 1982. 2005;60: 1739–1749. doi:10.1016/j.socscimed.2004.08.027

9. Camlin CS, Kwena ZA, Dworkin SL, Cohen CR, Bukusi EA. "She mixes her business": HIV transmission and acquisition risks among female migrants in western Kenya. Soc Sci Med 1982. 2014;102: 146–156. doi:10.1016/j.socscimed.2013.11.004

10. McGrath N, Eaton JW, Newell M-L, Hosegood V. Migration, sexual behaviour, and HIV risk: a general population cohort in rural South Africa. Lancet HIV. 2015;2: e252–e259. doi:10.1016/S2352-3018(15)00045-4

11. Rai T, Lambert HS, Ward H. Complex routes into HIV care for migrant workers: a qualitative study from north India. AIDS Care. 2015;27: 1418–1423. doi:10.1080/09540121.2015.1114988

For peer review only - http://bmjopen.bmj.com/site/about/guidelines.xhtml

12. Baral S, Beyrer C, Muessig K, Poteat T, Wirtz AL, Decker MR, et al. Burden of HIV among female sex workers in low-income and middle-income countries: a systematic review and meta-analysis. Lancet Infect Dis. 2012;12: 538–549. doi:10.1016/S1473-3099(12)70066-X

13. NSWP. Stigma and Discrimination Experienced by Sex Workers Living with HIV [Internet]. 2015 Dec. Available: http://www.nswp.org/resource/stigma-and-discrimination-experienced-sex-workers-living-hiv

14. Lim S, Peitzmeier S, Cange C, Papworth E, LeBreton M, Tamoufe U, et al. Violence against female sex workers in Cameroon: accounts of violence, harm reduction, and potential solutions. J Acquir Immune Defic Syndr 1999. 2015;68 Suppl 2: S241-247. doi:10.1097/QAI.0000000000000440

15. Tounkara FK, Diabaté S, Guédou FA, Ahoussinou C, Kintin F, Zannou DM, et al. Violence, condom breakage and HIV infection among female sex workers in Benin, West Africa. Sex Transm Dis. 2014;41: 312–318. doi:10.1097/OLQ.0000000000000114

16. O'Brien S, Broom A. Gender, culture and changing attitudes: experiences of HIV in Zimbabwe. Cult Health Sex. 2013;15: 583–597. doi:10.1080/13691058.2013.776111

17. Elmes J, Skovdal M. A reconfiguration of the sex trade: How social and structural changes in eastern Zimbabwe left women involved in sex work and transactional sex more vulnerable. PLOS ONE. (accepted).

18. Mtetwa S, Busza J, Chidiya S, Mungofa S, Cowan F. "You are wasting our drugs": health service barriers to HIV treatment for sex workers in Zimbabwe. BMC Public Health. 2013;13: 698. doi:10.1186/1471-2458-13-698

19. Scorgie F, Nakato D, Harper E, Richter M, Maseko S, Nare P, et al. "We are despised in the hospitals": sex workers' experiences of accessing health care in four African countries. Cult Health Sex. 2013;15: 450–465. doi:10.1080/13691058.2012.763187

20. Govindasamy D, Meghij J, Negussi EK, Baggaley RC, Ford N, Kranzer K. Interventions to improve or facilitate linkage to or retention in pre-ART (HIV) care and initiation of ART in low- and middle-income settings – a systematic review. J Int AIDS Soc. 2014;17. doi:10.7448/IAS.17.1.19032

21. Campbell C, Scott K, Nhamo M, Nyamukapa C, Madanhire C, Skovdal M, et al. Social capital and HIV Competent Communities: The role of community groups in managing HIV/AIDS in rural Zimbabwe. AIDS Care. 2013;25: S114–S122. doi:10.1080/09540121.2012.748170

22. Campbell C, Cornish F. How Can Community Health Programmes Build Enabling Environments for Transformative Communication? Experiences from India and South Africa. AIDS Behav. 2012;16: 847–857. doi:10.1007/s10461-011-9966-2

23. Kerrigan DL, Fonner VA, Stromdahl S, Kennedy CE. Community empowerment among female sex workers is an effective HIV prevention intervention: a systematic review of the peer-reviewed evidence from low- and middle-income countries. AIDS Behav. 2013;17: 1926–1940. doi:10.1007/s10461-013-0458-4

For peer review only

For peer review only - http://bmjopen.bmj.com/site/about/guidelines.xhtml

24. Batona G, Gagnon M-P, Simonyan DA, Guedou FA, Alary M. Understanding the intention to undergo regular HIV testing among female sex workers in Benin: a key issue for entry into HIV care. J Acquir Immune Defic Syndr 1999. 2015;68 Suppl 2: S206-212. doi:10.1097/QAI.0000000000000452

25. Siziya S, Marowa E, Mbengeranwa L, Latiff A. Sexually transmitted diseases in Zimbabwe: A qualitative analysis of factors associated with choice of a health care facility. Afr Health Sci. 2005;5: 114–118.

26. Freeman EE, Weiss HA, Glynn JR, Cross PL, Whitworth JA, Hayes RJ. Herpes simplex virus 2 infection increases HIV acquisition in men and women: systematic review and meta-analysis of longitudinal studies. AIDS Lond Engl. 2006;20: 73–83.

27. Atashili J, Poole C, Ndumbe PM, Adimora AA, Smith JS. Bacterial vaginosis and HIV acquisition: A meta-analysis of published studies. AIDS Lond Engl. 2008;22: 1493–1501. doi:10.1097/QAD.0b013e3283021a37

28. Elmes J, Nhongo K, Ward H, Hallett T, Nyamukapa C, White PJ, et al. The Price of Sex: Condom Use and the Determinants of the Price of Sex Among Female Sex Workers in Eastern Zimbabwe. J Infect Dis. 2014;210: S569–S578. doi:10.1093/infdis/jiu493

29. Goh CC, Kang SJ, Sawada Y. How did Korean households cope with negative shocks from the financial crisis? J Asian Econ. 2005;16: 239–254. doi:10.1016/j.asieco.2005.01.006

30. Tlhajoane M, Eaton JW, Takaruza A, Rhead R, Maswera R, Schur N, et al. Prevalence and Associations of Psychological Distress, HIV Infection and HIV Care Service Utilization in East Zimbabwe. AIDS Behav. 2017; 1–11. doi:10.1007/s10461-017-1705-x

31. Hatcher A, Smout E, Turan J, Christofides N, Stockl H. Intimate partner violence and engagement in HIV care and treatment among women: a systematic review and meta-analysis. AIDS. 2015;

32. Duff P, Goldenberg S, Deering K, Montaner J, Nguyen P, Dobrer S, et al. Barriers to Viral Suppression Among Female Sex Workers: Role of Structural and Intimate Partner Dynamics. JAIDS J Acquir Immune Defic Syndr. 2016;73. Available: http://journals.lww.com/jaids/Fulltext/2016/09010/Barriers_to_Viral_Suppression_Among_Female_Sex.12.aspx

33. Shahmanesh M, Patel V, Mabey D, Cowan F. Effectiveness of interventions for the prevention of HIV and other sexually transmitted infections in female sex workers in resource poor setting: a systematic review. Trop Med Int Health. 2008;13: 659–679. doi:10.1111/j.1365-3156.2008.02040.x

34. Medley A, Kennedy C, O'Reilly K, Sweat M. Effectiveness of Peer Education Interventions for HIV Prevention in Developing Countries: A Systematic Review and Meta-Analysis. AIDS Educ Prev. 2009;21: 181–206. doi:10.1521/aeap.2009.21.3.181

For peer review only - http://bmjopen.bmj.com/site/about/guidelines.xhtml

35. Campbell C, Cornish F. Towards a “fourth generation” of approaches to HIV/AIDS management: creating contexts for effective community mobilisation. AIDS Care. 2010;22: 1569–1579. doi:10.1080/09540121.2010.525812

36. Busza J, Walker D, Hairston A, Gable A, Pitter C, Lee S, et al. Community-based approaches for prevention of mother to child transmission in resource-poor settings: a social ecological review. J Int AIDS Soc Vol 15 Suppl 2 2012. 2012; Available: http://www.jiasociety.org/jias/index.php/jias/article/view/17373/688

37. Mugavero MJ, Amico KR, Horn T, Thompson MA. The State of Engagement in HIV Care in the United States: From Cascade to Continuum to Control. Clin Infect Dis. 2013;57: 1164–1171. doi:10.1093/cid/cit420

38. Patel V, Simunyu E, Gwanzura F, Lewis G, Mann A. The Shona Symptom Questionnaire: the development of an indigenous measure of common mental disorders in Harare. Acta Psychiatr Scand. 1997;95: 469–475.

For peer review only

For peer review only - http://bmjopen.bmj.com/site/about/guidelines.xhtml

STROBE Statement—checklist of items that should be included in reports of observational studies

	Item No.	Recommendation	Page No.	Relevant line no. from manuscript
**Title and abstract**	1	(*a*) Indicate the study's design with a commonly used term in the title or the abstract	2	40-41
		(*b*) Provide in the abstract an informative and balanced summary of what was done and what was found	2	40-55
**Introduction**				
Background/rationale	2	Explain the scientific background and rationale for the investigation being reported	4-5	77-102
Objectives	3	State specific objectives, including any prespecified hypotheses	5	104-112
**Methods**				
Study design	4	Present key elements of study design early in the paper	6	117-126
Setting	5	Describe the setting, locations, and relevant dates, including periods of recruitment, exposure, follow-up, and data collection	6-8	131-169
Participants	6	(*a*) *Cohort study*—Give the eligibility criteria, and the sources and methods of selection of participants. Describe methods of follow-up<br>*Case-control study*—Give the eligibility criteria, and the sources and methods of case ascertainment and control selection. Give the rationale for the choice of cases and controls<br>*Cross-sectional study*—Give the eligibility criteria, and the sources and methods of selection of participants	7	155-161
		(*b*) *Cohort study*—For matched studies, give matching criteria and number of exposed and unexposed<br>*Case-control study*—For matched studies, give matching criteria and the number of controls per case	NA	
Variables	7	Clearly define all outcomes, exposures, predictors, potential confounders, and effect modifiers. Give diagnostic criteria, if applicable	8-10	171-227
Data sources/ measurement	8*	For each variable of interest, give sources of data and details of methods of assessment (measurement). Describe comparability of assessment methods if there is more than one group	8-10	171-227
Bias	9	Describe any efforts to address potential sources of bias	7,8	146-148,172-179
Study size	10	Explain how the study size was arrived at	Reference to	132-135, 157-158

For peer review only - http://bmjopen.bmj.com/site/about/guidelines.xhtml

			other papers detailing methods on p 6-7	
Quantitative variables	11	Explain how quantitative variables were handled in the analyses. If applicable, describe which groupings were chosen and why	8-10	171-227
Statistical methods	12	(*a*) Describe all statistical methods, including those used to control for confounding	10-11	230-240
		(*b*) Describe any methods used to examine subgroups and interactions	10-11	230-240
		(*c*) Explain how missing data were addressed	8	178-179 (Missingness was low [<2%] and we were able to conduct a complete case analysis)
		(*d*) *Cohort study*—If applicable, explain how loss to follow-up was addressed<br>*Case-control study*—If applicable, explain how matching of cases and controls was addressed<br>*Cross-sectional study*—If applicable, describe analytical methods taking account of sampling strategy		Probabilistic matching of targeted survey respondents with records in general population survey (165-166, 176-179)
		(*e*) Describe any sensitivity analyses	NA	NA
**Results**				
Participants	13*	(a) Report numbers of individuals at each stage of study—eg numbers potentially eligible, examined for eligibility, confirmed eligible, included in the study, completing follow-up, and analysed	12	243-249
		(b) Give reasons for non-participation at each stage	12	243-249
		(c) Consider use of a flow diagram	NA	NA
Descriptive data	14*	(a) Give characteristics of study participants (eg demographic, clinical, social) and information on exposures and potential confounders	13 (see also table 1)	275-284 and table 1
		(b) Indicate number of participants with missing data for each variable of interest	Table 1	
		(c) *Cohort study*—Summarise follow-up time (eg, average and total amount)		
Outcome data	15*	*Cohort study*—Report numbers of outcome events or summary measures over time		
		Case-control study—Report numbers in each exposure category, or summary measures of		

For peer review only - http://bmjopen.bmj.com/site/about/guidelines.xhtml

		exposure		
		Cross-sectional study—Report numbers of outcome events or summary measures	12-13	258-269
Main results	16	(*a*) Give unadjusted estimates and, if applicable, confounder-adjusted estimates and their precision (eg, 95% confidence interval). Make clear which confounders were adjusted for and why they were included	13-14	290-304, 309-316 and table 2 & 3
		(*b*) Report category boundaries when continuous variables were categorized	Table 1 p25	Tables 1,2,3
		(*c*) If relevant, consider translating estimates of relative risk into absolute risk for a meaningful time period	NA	
Other analyses	17	Report other analyses done—eg analyses of subgroups and interactions, and sensitivity analyses	13-15	290-304, 309-316
**Discussion**				
Key results	18	Summarise key results with reference to study objectives	16	322-330
Limitations	19	Discuss limitations of the study, taking into account sources of potential bias or imprecision. Discuss both direction and magnitude of any potential bias	18	370-381
Interpretation	20	Give a cautious overall interpretation of results considering objectives, limitations, multiplicity of analyses, results from similar studies, and other relevant evidence	17-18	330-368,383-393
Generalisability	21	Discuss the generalisability (external validity) of the study results	18	383-393
**Other information**				
Funding	22	Give the source of funding and the role of the funders for the present study and, if applicable, for the original study on which the present article is based	19	411-420

*Give information separately for cases and controls in case-control studies and, if applicable, for exposed and unexposed groups in cohort and cross-sectional studies.

Note: An Explanation and Elaboration article discusses each checklist item and gives methodological background and published examples of transparent reporting. The STROBE checklist is best used in conjunction with this article (freely available on the Web sites of PLoS Medicine at http://www.plosmedicine.org/, Annals of Internal Medicine at http://www.annals.org/, and Epidemiology at http://www.epidem.com/). Information on the STROBE Initiative is available at www.strobe-statement.org.

For peer review only - http://bmjopen.bmj.com/site/about/guidelines.xhtml
